# Identifying molecules as biosignatures with assembly theory and mass spectrometry

**DOI:** 10.1038/s41467-021-23258-x

**Published:** 2021-05-24

**Authors:** Stuart M. Marshall, Cole Mathis, Emma Carrick, Graham Keenan, Geoffrey J. T. Cooper, Heather Graham, Matthew Craven, Piotr S. Gromski, Douglas G. Moore, Sara. I. Walker, Leroy Cronin

**Affiliations:** 1 School of Chemistry, University of Glasgow, Glasgow, UK; 2 Astrobiology Analytical Laboratory, NASA Goddard Space Flight Center, Greenbelt, MD USA; 3 Beyond Centre for Concepts in Fundamental Science, Arizona State University, Tempe, AZ USA

**Keywords:** Mass spectrometry, Astrobiology, Cheminformatics, Origin of life

## Abstract

The search for alien life is hard because we do not know what signatures are unique to life. We show why complex molecules found in high abundance are universal biosignatures and demonstrate the first intrinsic experimentally tractable measure of molecular complexity, called the molecular assembly index (MA). To do this we calculate the complexity of several million molecules and validate that their complexity can be experimentally determined by mass spectrometry. This approach allows us to identify molecular biosignatures from a set of diverse samples from around the world, outer space, and the laboratory, demonstrating it is possible to build a life detection experiment based on MA that could be deployed to extraterrestrial locations, and used as a complexity scale to quantify constraints needed to direct prebiotically plausible processes in the laboratory. Such an approach is vital for finding life elsewhere in the universe or creating de-novo life in the lab.

## Abstract (web summary)

The search for life in the universe is difficult due to issues with defining signatures of living systems. Here, the authors present an approach based on the molecular assembly number and tandem mass spectrometry that allows identification of molecules produced by biological systems, and use it to identify biosignatures from a range of samples, including ones from outer space.

## Introduction

The search for evidence of life elsewhere in the universe relies on data collected from probes in our solar system, or astronomical observations^1,2^. Knowing what signatures can be assigned to living systems is difficult as alien life has never been seen before^3^. A solution would be to identify a feature exclusively associated with all life^4^ and develop a detection system for that feature^5^. Here we postulate that living systems can be distinguished from non-living systems as they produce complex molecules in abundance which cannot form randomly^6^ so what is needed is a way to quantify this complexity^7^. The need for a new technique is important for our understanding of life in the universe. The recent discoveries of the ubiquity of exo-planets^8,9^ of which there are over four thousand reported^10^, raises the prospect that we will be able to observe planets that harbor life within a few decades^11^. However, we don’t yet have a scientifically rigorous way to distinguish planets that host life from those that do not, even in our solar system. The design of a remote, unambiguous, and practical extraterrestrial life detection experiment is difficult because we have no idea what alien biochemistry is possible beyond Earth^12^. This difficulty stems from the fact we have no universal definition of life because we only have the example of terrestrial life, and all known life forms are thought to have descended from a common ancestor. Many operational definitions of life emphasize the role of Darwinian evolution in living systems, but it is not clear how this criterion can be translated into an unambiguous remote experiment^13,14^.

It is possible to distinguish between living and non-living systems on Earth due to processes such as photosynthesis, carbon and nitrogen fixation, replication, chiral enrichment, and morphogenesis^15–17^. The artefacts of these processes have been proposed as possible biosignatures. There are proposals to search for such artefacts in minerals, and via isotopic and atmospheric analysis. The problem with looking for such processes in a universal manner is the lack of a rigorous definition outside the context of known terrestrial biochemistry, and therefore these cannot be deployed to design experiments. This has led to several ambiguous results from ‘metabolic’ experiments done by the Viking Lander^1^ on Mars, and investigations of potential meteorite ‘microfossils’^18^. The results of these experiments were ambiguous because they could not be understood in a quantitative theoretical framework, and therefore the interpretation depended on chemical unknowns. In the case of the Viking Lander’s ‘metabolic’ experiments, the properties of Martian soil were unknown, making it difficult to determine whether the observed responses were purely abiotic in nature, or driven by biological processes. In the case of the supposedly biogenic magnetite crystals in the ALH 84001 meteorite, the criteria used to demarcate biogenic activity from abiogenic activity was not a quantitative measure, meaning the interpretation was always going to be ambiguous^19^.

To circumvent these difficulties, we hypothesized that the very complex molecules made in any biochemical system could be distinguished from those produced in a non-biochemical system^6^. This is because living systems appear to be uniquely able to produce a large abundance of complex molecules that are inaccessible to abiotic systems^7^, where the number of small organic molecules, allowed by the rules of chemistry, is practically infinite. For instance, more than 10^60^ ‘drug-like’ organic molecules with a molecular weight below 500 Daltons are estimated to be possible^20^. Making any of these possible molecules from precursors requires constraints that may be naturally occurring, such as kinetic and thermodynamic properties of a reaction pathway^21^, or are synthetically imposed. Biochemical systems appear to be able to generate almost infinite complexity because they have information decoding and encoding processes that drive networks of complex reactions to impose the numerous, highly specific constraints needed to ensure reliable synthesis^22,23^. For example, the natural product Taxol^24^, is an example of a molecule that could be a biosignature–this is because it is so complicated, that the probability of its formation abiotically in any detectable abundance (>10,000 identical copies) would be very small. One reason for this is that there are at least more than 10^23^ different molecules possible^25^ with the same formula as Taxol, C_47_H_51_NO_14_ (molecular weight of 853.9), and this analysis excludes the fact that Taxol incorporates 11 chiral centers which means it has 2^11^ or 2048 possible diastereomers. The selection of one such possibility out of the combinatorically large number of possibilities is a process that requires information. In the absence of such information encoding and decoding processes, relatively few constraints can be placed on a chemical system–only those that are encoded in the laws of physics and the local environment–which cannot provide the specific set of biases needed to produce complex molecules such as Taxol in abundance.

Here we develop assembly theory and its application to molecular complexity. The concept of molecular complexity has been extensively explored theoretically^26^, with many metrics devised based upon structural, topological, or graph theoretical complexity. However, all these metrics have different algorithms, and none have an experimental measure^27^. To address this problem, we have devised a theory of molecular complexity that is experimentally verifiable. By mapping the complexity of molecular space it is possible to place molecules on a scale of complexity from those able to form abiotically to those so complex they require a vast amount of encoded information to produce their structures, which means that their abiotic formation is very unlikely. This mapping can be experimentally verified by building a model that correlates the theoretical complexities with spectroscopic data. By applying this model to a range of standard molecules as well as analog samples from the laboratory, terrestrial, and marine environmental samples, and an extraterrestrial sample, we show it is possible to unambiguously distinguish samples that contain molecules produced by life from those that do not.

## Results

### Defining molecular assembly

**Fig. 1:**
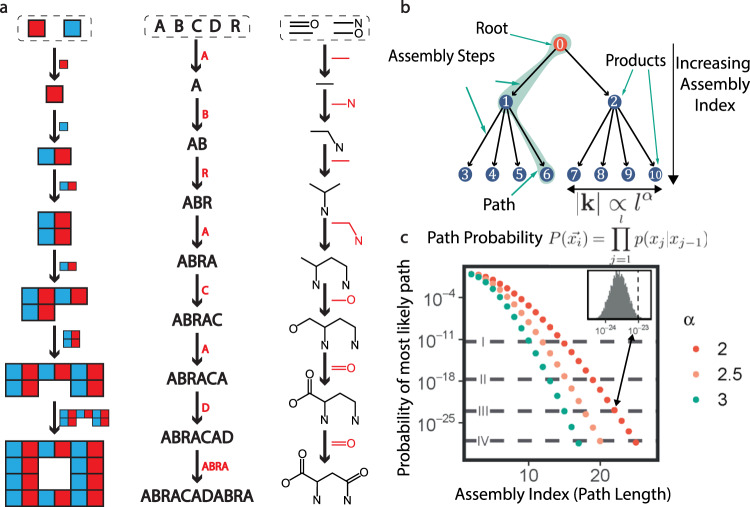
Assembly pathways. **a** In analyzing the assembly pathways of an object, we start with its basic building blocks, which are the shared set of objects that can construct our target object and any other object within the class of objects. The Assembly index of an object is defined as the smallest number of joining operations required to create the object using this model. **b** We can model the assembly process as a random walk on weighted trees where the number of outgoing edges (leaves) grows as a function of the depth of the tree, due to the addition of previously made sub-structures. By generating several million trees and calculating the likelihood of the most likely path through the tree, we can estimate the likelihood of an object forming by chance as a function of the number of joining operations required (path length). **c** The probability of the most likely path through the tree as a function of the path length decreases rapidly. The colors indicate different assumptions about the chemical space. For comparison, the dashed lines indicate the ratio of (I) one star in the entire milky way, 1:10^11^, (II) one gram out of all of Earth’s biomass, 1:10^17^, (III) one in a mole, 1:10^23^, and (IV) one gram out of Earth’s mass (1:10^29^). Note on this plot the path probability of the formation of Taxol would vary between 1:10^35^ to 1:10^60^ with a path length of 30 and the amount of chemical predisposition is varied with alpha biasing the effective selectivity between 50–99.9% at each step respectively.

Our approach to life detection is based on the molecular assembly number (MA) which is derived from the theory of assembly pathways^7^. Assembly pathways are sequences of joining operations, that start with basic building blocks (in this case bonds) and end with a final product. In these sequences, sub-units generated within the sequence can combine with other basic or compound sub-units later in the sequence, to recursively generate larger structures (see Fig. 1A). Assembly pathways have been formalized mathematically using directed multigraphs (graphs where multiple edges are permitted between two vertices) with objects as vertices and objects as edge labels^7^, however for the results here the formal details are unnecessary (see the Supplementary Information for more details). Generating many assembly pathways from a pool of basic units will result in a combinatorial explosion in the diversity of structures. The molecular assembly number (MA) therefore captures the specificity of the molecule in the face of this combinatorial explosion and provides an agnostic measure of the likelihood for any molecular structure to be produced more than once. There will normally be multiple assembly pathways to create a given molecule. The MA of an object is the length of the shortest of those pathways, i.e. the smallest number of joining operations requires to construct the object, where objects created in the process can subsequently be reused. Thus, it is a simple integer metric to indicate the number of steps required in this idealized case to construct the molecule.

The MA of a molecule represents a best-case construction pathway by accounting for valence rules but no other limits from chemistry, including no consideration of reaction conditions. Importantly, a molecule with a relatively low MA could be difficult to synthesize in the laboratory, and hence as a tool for life detection, MA is susceptible to false negatives. Critically, life detection based on MA measurements can be made robust against false positives, as the number of synthetic steps required to create the molecule is not likely to be lower than the steps within the assembly model^28^ (see Supplementary Information section 2 for details). Our central thesis is that molecules with high MA are very unlikely to form abiotically, and the probability of abiotic formation goes down as MA increases, and hence experimental determination of MA is a good candidate for a life detection system^29^. If our hypothesis is correct, then life detection experiments based on MA can indicate the presence of living systems, irrespective of their elemental composition, assuming those living systems are based on molecules.

### Bounding the MA probabilistically

To help determine how the probability of the spontaneous formation of detectable amounts of any given molecule changes with MA, we developed a computational model for the assembly of molecular graphs as unique steps on a path, where the length of this path represents the MA of a given molecule. The formation of molecules through the assembly process is then modeled using random walks on weighted trees^30^, for full details see SI. Briefly, in this model the root of the tree corresponds to bonds available, while the nodes correspond to possible combinations of those bonds. Each node in the tree corresponds to molecules that could be synthesized from the available bonds, while outgoing edges represent joining operations transforming one molecule into another with the addition of more bonds. The shortest number of steps on a path from the base of the tree to the end of the branch corresponds to the MA of that compound, see Fig. 1B. To map the probability of the formation of any given molecule as a function of MA, we generated 3 million trees with different properties and determined the highest probability of formation of molecules as a function of MA. The probabilities we calculated represent the likelihood of an unconstrained or random assembly process generating that specific compound, given that the abiotic precursors are so abundant that they do not limit the formation. These probabilities do not represent the absolute probability of a molecule ever forming, rather they represent the chance of the molecule forming in the unconstrained, undirected process described herein any detectable abundance.

Thermodynamic and kinetic considerations suggest that the relative rates of reactions vary by orders of magnitude, and we implemented this in our model by assigning edge weights (and therefore relative abiotic likelihoods of those reactions) that also span multiple orders of magnitude. The number of possible products for each node in the trees grows as a function of the depth and hence MA of the node. By modeling the rate of growth using a function of the form |k| $\propto {l}^{\alpha },$ where |*k*| is the number of possible molecules, *l* is the depth of the node (*l* is equal to MA for a given molecule) and *α* is a parameter that controls how quickly the number of joining operations growths with the depth of the tree. For the combination of any two molecules, the number of possible products formed from their combination grows at least linearly with the size of the compounds, since the bigger compounds have more atoms between which bonds can form. This means the number of ways to produce products in an assembly path explodes as the MA increases since the paths recursively utilize previous steps. To capture this, we evaluated the model with values of *α* between two and three, where two indicated the most conservative quadratic growth rate, and three representing a limiting case where both factors grow super-linearly. Under these conditions, molecules with a MA of between 15 and 20 would have a chance formation of one molecule in 10^23^, or one molecule in a mole of substance respectively. We consider the one molecule in a mole threshold significant since it would be much lower than the detection limit for any known analytical technique. Importantly, we’ve not assumed any specific details about the availability of resources or the stability of specific bonds. This means that these results are agnostic because they do not depend on the particulars of the chemistry, only on the size and accessibility of the chemical space. Our model shows that MA tracks the specificity of a path through the combinatorically vast chemical space, and this supports our thesis that high MA molecules cannot form in detectable abundance through random and unconstrained processes, implying that the existence of high MA molecules depends on additional constraints imposed on the process.

### Measuring MA in chemical space

**Fig. 2:**
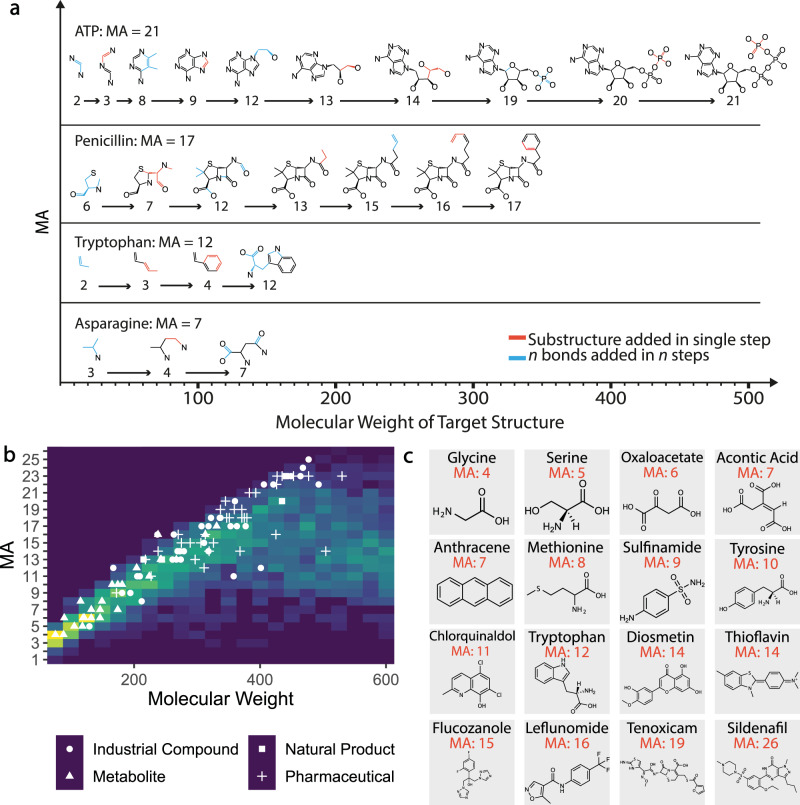
Molecular assembly and chemical space. **a** Schematic of assembly paths for four example molecules (hydrogens and charges omitted for clarity). **b** The computed MA of molecules from the Reaxys database shown by molecular weight. The color scale indicates the frequency, with increasing frequency from dark purple (0.0) to green and yellow (1.0) of molecules in a given molecular weight range with a given MA. 2.5 million MA were calculated, in the figure shown here that data has been subsampled to control for bias, see SI. The overlaid plot with the white labels shows how the MA varies for some compound types where some natural products, pharmaceuticals, and metabolites have a wide range of values (these molecules are listed in Supplementary Information Section 7, table 2). Note that the range of MA for the amino acids is limited. The molecular masses are binned in 50 Dalton sections. **c** Example organic molecular structures and the corresponding MA values calculated.

In order to explore how MA is distributed in real molecules, we needed a way to compute the MA of a molecule, and thus we have devised an approach that uses bonds as the basic units, which simplifies our computation (Fig. 2, see Supplementary Information Section 2 for details). In using bonds as the basic unit, we describe structures as bonds connected by atoms, where two bonds are joined by superimposing an atom from each. Computing an assembly pathway of a molecule can be done simply by decomposing the object into fragments and reconstructing it, however, identifying the shortest pathway is computationally challenging.

We, therefore, developed an algorithm that calculates the “split-branch” variant of MA, implementing an efficient search for assembly pathways by recursively finding duplicated structures within the molecule (see Supplementary Information section 1 for full details). In this algorithm, the pathway found may not be the shortest, and so the result is an upper bound for the MA. However, the value calculated provides a robust estimate for the MA of the molecules in the work presented here. The algorithmic implementation of MA was used to characterize chemical space as represented by the Reaxys® database^7,31^. We retrieved 25 million compounds with molecular mass up to 1000 Daltons from the database and the MA was calculated for a subset of 2.5 million unique structures over a molecular mass range up to 800 Daltons, see Fig. 2b, which shows how the MA of molecules in Reaxys vary with molecular weight. These results show that for small molecules (mass < ~250 Daltons) the MA is strongly constrained by their mass. This is understandable because small molecules have limited compositional diversity and few structural asymmetries. The MA of molecules with a mass greater than ~250 Daltons appear to be less determined by molecular weight, indicating that they can display vastly more compositional and structural heterogeneity. This is significant because it gives us insight into how to develop an experimental measure of MA based on tandem mass spectrometry by focusing on fragmenting molecules that have a mass greater than 250 Daltons.

### Measuring MA in real molecules

**Fig. 3:**
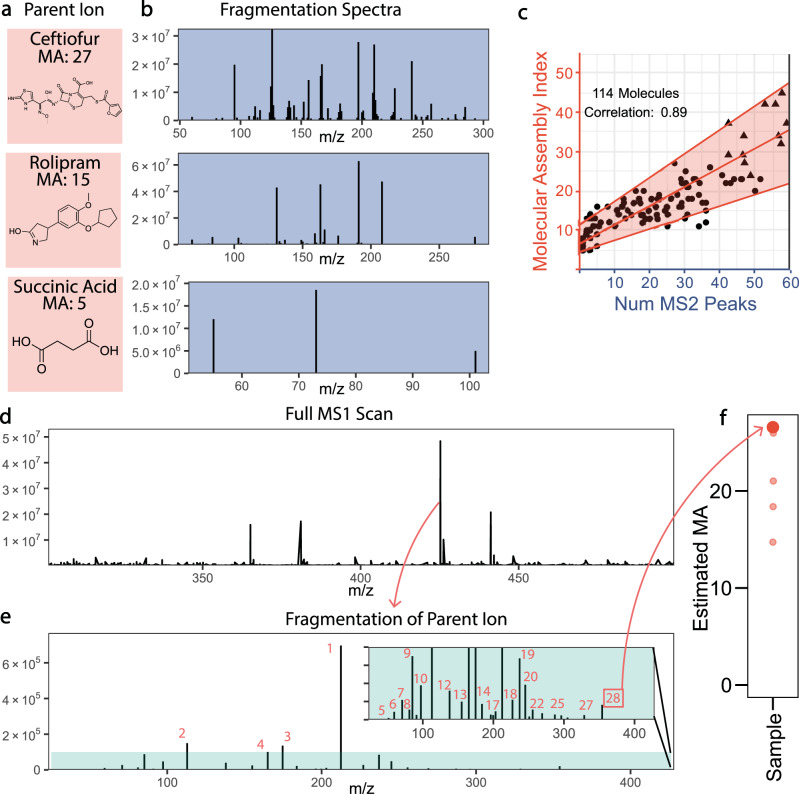
Experimental correlation of mass spectrometry data to MA and MA analysis of mixtures. **a** Three example molecular structures with associated MA index. **b** The fragmentation spectra associated with the molecular ions from (A). The high MA molecules have more peaks in their fragmentation spectra. **c** The observed correlation between the number of peaks in a fragmentation spectrum and the MA value of the ion, the shaded region shows the 90% prediction interval using quantile regression, with the median prediction shown in the center line. The circles represent small organics while triangles represent peptides. D-F indicate analytical workflow for measuring MA in mixtures. **d** A single ion is selected based on intensity. **e** MS2 spectra from the selected ion, with the inset showing the same spectra zoomed in on the shaded region to show lower intensity peaks. The total number of peaks in the fragmentation spectra are counted to correlate with the MA. **f** Many ions from the mixture will be fragmented and the predicted MA from that sample form a distribution, we consider the highest MA value measured to represent the MA of the mixture.

Having established a method to calculate MA and explored how it varies in known chemical space, we next developed an analytical method to correlate experimental data to MA directly. Since MA is closely related to the structural heterogeneity of molecules, we developed a method based on tandem mass spectrometry (MS/MS). That approach allowed us to resolve distinct fragmentation patterns between high and low MA molecules. Tandem MS provides advantages in terms of life detection experiments because it generates separate signals for different ions in real complex mixtures. This separation is critical since MA is a measure based on individual molecules. Our central hypothesis is that high MA molecules will generate MS2 spectra with many distinct peaks, and that lower MA molecules would generate proportionally fewer since they tend to have fewer bonds and more symmetry, see Fig. 3. To test this hypothesis, we collected MS2 spectra for >100 small molecules and peptides for which we had calculated MA (sample preparation details are in Supplementary Information Section 4).

We analyzed these molecules using an Orbitrap Fusion Lumos Tribrid mass spectrometer and compared the number of MS2 peaks in the spectra to the calculated MA for all molecules. This instrument has a much higher resolution than mass spectrometers expected on planned space missions, and our goal here is to demonstrate the viability of this analysis in principle. The results are shown in Fig. 3C, where each point represents a unique molecule. To count the number of MS2 peaks for known molecules we use Single Ion Monitoring (SIM) to ensure we fragmented the ion with the exact mass of the known molecule. Noise filtering, and peak counting are described in detail in Section 4 of SI. Some molecules were analyzed multiple times and the number of MS2 peaks was averaged (see more details in Section 4 of SI). Our analysis demonstrated a linear relationship, with a correlation of 0.89, between the number of MS2 peaks generated by a fragmented ion and its MA. Once we had established a direct relationship between our theoretically determined MA, and an easily observable quantity (i.e., the number of peaks in a fragmentation spectrum), we moved on to testing our central thesis that high MA molecules do not form in detectable quantities in the absence of biological or technological processes.

### Life detection using MA measurements

**Fig. 4:**
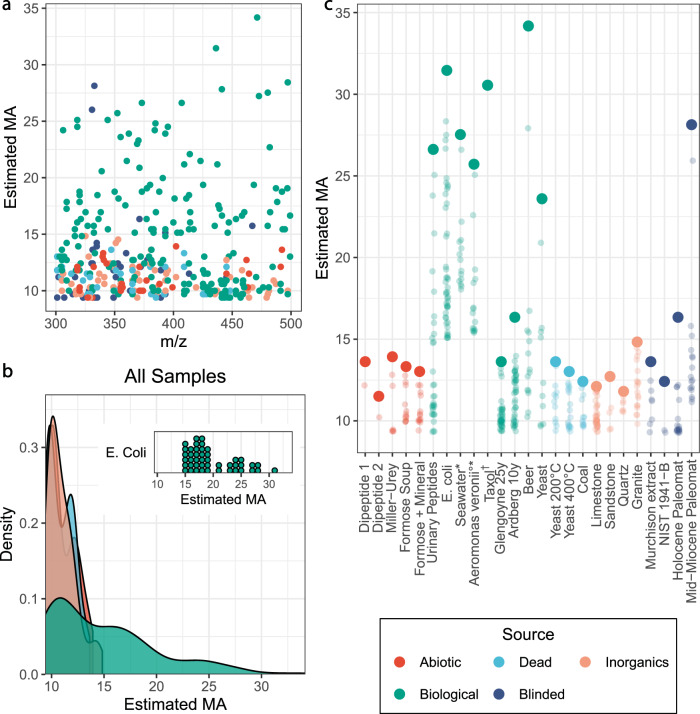
Estimated MA of laboratory and environmental samples. **a** The estimated MA against the parent mass of many ions for different samples in the 300–500 m/z range (excluding Taxol with has a m/z value of 854.9). **b** The distributions of estimated MA for all samples split by category, colored by source, the inset shows the distribution of points for a single biological sample, *E. Coli*. The MA of biological samples has a wider distribution, showing that only biologically produced samples produce MA above a certain threshold. **c** The estimated MA values for each sample with the blinded identities correctly labeled. The highest MA value in each sample is bold and the lower values faded. Each sample may have more than 15 points due to the dynamic exclusion settings used, which enable us to collect more MS2 peaks. Samples may have less than 15 points due to excluding noisy or unreliable spectra, for more information see Supplementary Information Section 5. *These samples were run with a column attached to the mass spectrometry but no chromatographic method was used. °This sample was gathered from an online database and analyzed with a different instrument. † Taxol is shown in Fig. 4C but has a mass that is not shown in Fig. 4A or 5B. See Supplementary Information Section 6 for details.

We collected MS2 spectra from a wide variety of mixtures, including prebiotic soups, biological, abiotic, inorganic, dead, and blinded samples. We used Data Dependent Acquisition (DDA) to acquire MS2 data from the most intense ions in the mixture, allowing these to be directly compared to the single compound samples measured previously (see more details in Section 4 of SI). The biological samples were explicitly produced by living systems such as *E. coli* lysates, yeast cultures, urinary peptides, a seawater sample, a highly complex natural product (Taxol) as well as fermented beverages and distillates (home-brewed beer and Scottish Whisky). Abiotic samples were produced in a controlled synthesis without enzymes or other biological influence (besides the chemists that prepared them) and included dipeptides, and Miller–Urey spark discharge mixtures. We also investigated the incredibly messy sugar-based ‘formose’ reaction mixtures with and without mineral salts added^32^. Inorganic samples included extracts from terrestrial mineral sources such as quartz, limestone, sandstone, and granite. Dead samples, which were taken from terrestrial sources that have been influenced by biological processes but are not alive, included coal, and yeast burned at 200 °C and 400 °C. Finally, the blinded samples were a collection of samples whose origin was unknown at the time of analysis. These included a CM2 carbonaceous chondrite sample (Murchison meteorite), bay sediment, and biological material from two different geological epochs, the Holocene (~30,000 years old) and the Mid-Miocene ~14 Ma^33^. In addition, we analyzed a sample of the bacteria *Aeromonas veronii* collected via an online repository^34^, it was analyzed with a different analytical platform but the results confirm our analysis, see Supplementary Information Section 6 for details. All samples we extracted in a mixture of water and methanol, with other details of the extraction listed in the Supplementary Information Section 6. The samples were analyzed directly in the mass spectrometer without any chromatography, see Fig. 4.

For each sample, our goal was to fragment as many of the most intense MS1 peaks as possible while still collecting reliable MS2 spectra from those peaks. We used data-dependent acquisition to fragment the 15 most intense parent ions in the MS1 spectra. Based on our analysis of the Reaxys database we focused on MS1 peaks in the m/z range of 300–500 because our analysis indicated molecules in that mass range take on a diverse set of MA values (see Figure 2a). By fragmenting the most intense MS1 peaks we were able to collect many distinct MS2 spectra from the mixtures. By determining the number of MS2 peaks in these spectra, and using the observed correlation from our analysis of single molecules, we can estimate the MA of the different ions found in the mixtures. In complex mixtures, there is a chance that multiple isomers could be selected for fr agmentation simultaneously, we’ve taken steps to mitigate the effects of possibility and we do not believe it has affected the results here, see Supplementary Information Section 5. Figure 3D–F shows an illustration of this workflow indicating the selected peak in the MS1 spectra (D), the associated MS2 spectra with the peaks counted, with the inset zoomed in on the same data to show the lower intensity peaks (E), and the predicted MA for that MS2 spectra and others from the same mixture (F).

These results demonstrate that we can identify the living systems by looking for mixtures with an MA greater than a certain threshold, see Fig. 4. In the case of the analysis presented here it appears that only living samples produced MA measurement above ~15. Importantly this measurement does not imply that samples with a maximum MA below 15 are non-living, on the contrary several samples made or altered by living systems failed to generate MA above this threshold such as the Bay Sediment and some of the Scottish whisky. These represent false negatives, and they illustrate an important feature of MA-based life detection protocols: not all molecules produced by living processes have high MA–indeed complex mammals regularly produce CO_2_–but all high MA molecules are produced by living (or technological) processes. This can be seen clearly in Fig. 4b, which shows the distribution of MA values across all samples, separated by source, such that all the peaks from all the biological samples are shown in one color, all the peaks from all the dead samples shown in another, etc. The biological samples are the only ones that produce high MA molecules, since biological samples also produce low MA molecules, the biological distribution is broader. This is critical as it implies that looking for very high MA values in mixtures is an agnostic way to search for living systems.

## Discussion

This approach to exploring molecular complexity using molecular assembly allows the development of a measurable indication of complexity. Importantly we are able to evaluate real samples without the need for complex preparation or chromatography, the only critical analytical feature for this analysis is the ability to fragment individual ions. As such we have shown that our approach to life detection using molecules in the mixtures as unique markers allows us to establish a threshold beyond which the molecules can be unambiguously determined as being produced by a living system.

We’ve shown this method can be applied to a wide range of samples and can detect biologically produced molecules even in very old samples around ~14 Ma (such as the Mid-Miocene material), indicating the method can detect living systems even if they are no longer active. Our system avoids the potential of false negatives and allows us to search the universe agnostically for evidence of what life does rather than attempting to define what life is^35,36^.

We have developed a scale of molecular assembly which is the first experimentally verifiable measure of molecular complexity. Using mass spectrometry, this method has been applied to a range of non-biological and biological samples demonstrating the utility and power of this method. The complexity measure works on unknown samples at the molecular level, as it is intrinsic to the molecule ions selected in the ion trap, and not a characteristic of the mixture when MS/MS is used as the detector. A key aspect of using MS/MS means that little sample preparation is needed, and chromatographic separation is not required. This is because complex mixtures are ignored, whilst intrinsically complex molecules can be unambiguously identified. This means that at MS/MS-based MA experiment can be used to identify the likelihood that the molecules found in a sample are derived from a biological or technological process. The approach can be used to map the wider environment and also look at distributions of molecular complexity in the environment as a function of time and space with MA^37^. These results demonstrate it is possible to use this method to build a life detection instrument that could be deployed on missions to extraterrestrial locations to detect biosignatures, map the extent of life on Earth, as well be used as a molecular complexity scale to quantify the constraints needed to direct prebiotically plausible processes towards the emergence of de-novo life in the laboratory.

## Methods

### Development of pathway assembly complexity for molecules

The molecular assembly algorithm, written in C++, implements the split branch variant of molecular assembly with bonds as the basic objects (See Supplementary Information for more details). It takes an MDL mol file as input, and outputs a single integer value as the molecular assembly index (MA). The MA is determined by searching through partitions of identical substructures, with the algorithm run recursively on those substructures where required. The split-branch MA is calculated by:

1 $$MA=\left\{\begin{array}{cc}\mathop{\sum }\limits_{i=1}^{n}(M{A}_{i}+{N}_{i}-1),&number\,of\,bonds > 1\hfill\\ \hfill1,&number\,of\,bonds=1\end{array}\right.$$

Where the sum is over each duplicated substructure, with *N*_*i*_ representing the number of such duplicates in the structure (the −1 is because the MA is based on the MA of the duplicated substructures plus the number of additional copies).

### Probabilistic algorithm for exploring molecular assembly

The computational model of molecular assembly pathways was implemented in R v3.5. Each edge weight was drawn from a distribution of the form ${w}_{i} \sim {10}^{{\mathscr{U}}(0,h)},$ where ${\mathscr{U}}(0,h)$ represents a uniform distribution between 0 and *h*, such that *h* controls how many orders of magnitude the weights vary over. The weights are normalized such that the total weight of all outgoing edges is one, and therefore each probability has a value between zero and one. The effect of changing *h* is shown in Supplementary Information Section 3. The results in the main text use *h* = 4 such that probabilities of any given joining operation vary over four orders of magnitude. The probability of each transition was calculated as the weight of the edges divided by all the edge weights emanating from the source node. The probability of each path was calculated by taking the product of all transition probabilities starting from the root and ending with the final product. Additional information in the Supplementary Information Section 3.

### Exploration of chemical space

In order to explore known chemical space, we used the Reaxys database, working with a set of ~25 million substances, representing all molecules in the database with molecular weight <1000. We calculated the MA of ~2.5 million of these, with molecules of high complexity and molecular weight not calculated due to computation limitations from the algorithm. The MA of the molecules was saved within a local postgresql database. Python scripts were used to extract/analyze the data and output figures. Further details can be found in the SI.

### Mass spectrometry workflow

All samples were analyzed by tandem mass spectrometry in an Orbitrap Fusion Lumos Tribrid mass spectrometer (Thermo, San Jose, CA, USA). Molecules analyzed for the standard curve calculation were introduced to the mass spectrometer via an Advion Nanomate (Ithaca, NY, USA). Samples of 15 µl were injected onto an emitter with a +1.2 KV voltage applied, the gas on the nanomate was set to 40 psi. Samples were analyzed for 6 mins, during which a Single Ion Monitoring (SIM) scan for a molecule exact mass was performed followed by a fragmentation event (MS2). This ensured fragmentation data were collected for the targeted analyte and not any potential contamination. The fragmentation method was HCD with fragmentation energies set at 45% for the first 3 mins and 35% for mins 3–6. The isolation window for MS2 fragmentation selection was set to 0.5 Da, the resolution of the SIM scan was 2,40,000 at 400 m/z and the resolution of the MS2 scans was 30,000 at 400 m/z. See Supplementary Information Section 4 and 5 for more details.

### MS environmental samples

For environmental samples analytical conditions were as above, however, the SIM scan was replaced by a MS1 survey Scan, and the 15 most intense peaks were selected for MS2. If ions were selected for fragmentation twice in 10 s, these were then excluded for the next 30 secs of analysis. The linear relationship was fit using quantile regression, where the upper line was fit with *τ* = 0.95, the middle line was the median fit with *τ* = 0.5, and the lower line was the fit with *τ* = 0.05, such that the shaded region shows the uncertainty in the relationship with 90% confidence, while the middle line shows the expected fit. See Supplementary Information Section 5 for further details.

## Supplementary information

Supplementary Information

## Data Availability

The supplementary information contains examples of raw data and processed data are provided with further instructions on how to use the code. Complete data set available upon request. Source data are provided with this paper.

## Source data

Source Data File

## Code availability

The software required to compute Molecular Assembly Indices from mol files as well as Python v3.7 code and R v3.5 code for processing mass spectrometry data is included in the supplementary information.

## References

[1] Ballou EV, Wood PC, Wydeven T, Lehwalt ME, Mack RE (1978). Chemical interpretation of Viking Lander 1 life detection experiment. Nature.

[2] Schwieterman EW (2018). Exoplanet biosignatures: a review of remotely detectable signs of life. Astrobiology.

[3] Selsis, F., Despois, D. & Parisot, J. P. Signature of life on exoplanets: can Darwin produce false positive detections? *Astron. and astrophys.* **388**, 985–1003 (2002).

[4] Gentry DM (2017). Correlations between life-detection techniques and implications for sampling site selection in planetary analog missions. Astrobiology.

[5] Lovelock JE (1965). A physical basis for life detection experiments. Nature.

[6] Marshall SM, Murray ARG, Cronin L (2017). A probabilistic framework for identifying biosignatures using Pathway Complexity. Philos. Trans. R. Soc. A: Math.,.

[7] Marshall, S. M., Moore, D., Murray, A. R. G., Walker, S. I. & Cronin, L. Quantifying the pathways to life using assembly space. Preprint at https://arxiv.org/abs/1907.04649 (2019) 10.3390/e24070884 PMC9323097 35885107

[8] Cassan, A. et al. One or more bound planets per Milky Way star from microlensing observations. *Nature* **481**, 167–169 (2012). 10.1038/nature10684 22237108

[9] Lissauer JJ, Dawson RI, Tremaine S (2014). Advances in exoplanet science from Kepler. Nature.

[10] Schneider, J. *The Extraolar Planet Encyclopaedia*. (2019). http://exoplanet.eu/

[11] Walker SI (2018). Exoplanet biosignatures: future directions. Astrobiology.

[12] Monod, J. In *Studies in the Philosophy of Biology: Reduction and Related Problems* (eds Francisco Jose Ayala & Theodosius Dobzhansky) 357–375 (Macmillan Education UK, 1974).

[13] Deamer, D. W. & Fleischaker, G. R. In *The Quarterly Review Of Biology Vol. 69–2* (Podolsky, S. H. & Tauber, A. I. eds) 253 (The University of Chicago Press, Chicago, 1994), 10.1086/418549.

[14] Mariscal C, Doolittle WF (2020). Life and life only: a radical alternative to life definitionism. Synthese.

[15] Kuchling F, Friston K, Georgiev G, Levin M (2020). Morphogenesis as Bayesian inference: a variational approach to pattern formation and control in complex biological systems. Phys. Life Rev..

[16] Breslow, R. & Levine, M. S. Amplification of enantiomeric concentrations under credible prebiotic conditions. *Proc. of the Natl. Acad. of Sci. of the USA* **103**, 12979–12980 (2006). 10.1073/pnas.0605863103 PMC1559738 16938839

[17] Schmidt GA, Frank A (2019). The Silurian hypothesis: would it be possible to detect an industrial civilization in the geological record?. Int. J. Astrobiol..

[18] Thomas-Keprta KL (2002). Magnetofossils from Ancient Mars: a robust biosignature in the martian meteorite ALH84001. Appl. Environ. Microbiol..

[19] Barber DJ, Scott ERD (2002). Origin of supposedly biogenic magnetite in the Martian meteorite Allan Hills 84001. Proc. Natl Acad. Sci..

[20] Bohacek RS, McMartin C, Guida WC (1996). The art and practice of structure-based drug design: A molecular modeling perspective. Medicinal Res. Rev..

[21] Eschenmoser A (2011). Etiology of potentially primordial biomolecular structures: from vitamin B12 to the nucleic acids and an inquiry into the chemistry of life’s origin: a retrospective. Angew. Chem. Int. Ed..

[22] Walker SI, Davies PCW (2013). The algorithmic origins of life. J. R. Soc. Interface.

[23] Smith, J. M. in *In the Scope of Logic, Methodology and Philosophy of Science. Philosophy of Science* 2 (Gärdenfors P., Woleński J., Kijania-Placek K. eds) 177–194 (Springer, Dordrecht, 2000), 10.1007/978-94-017-0475-5_18.

[24] Nicolaou KC (1994). Total synthesis of taxol. Nature.

[25] Paton RS, Goodman JM (2007). Exploration of the accessible chemical space of acyclic alkanes. J. Chem. Inf. Modeling.

[26] Nikolić, S., Trinajstić, N., Tolić, I. M., Rücker G. & Rücker, C. In *Complexity in Chemistry: Introduction and Fundamentals* (eds Rouvray, D. H., Bonchev, D.) 29-89 (Taylor & Francis, UK, 2003).

[27] Schmitt-Kopplin P (2019). Systems chemical analytics: introduction to the challenges of chemical complexity analysis. Faraday Discuss..

[28] Coley CW, Rogers L, Green WH, Jensen KF (2018). SCScore: synthetic complexity learned from a reaction corpus. J. Chem. Inf. Modeling.

[29] Neveu M, Hays LE, Voytek MA, New MH, Schulte MD (2018). The Ladder of Life Detection. Astrobiology.

[30] Zhang, Z., Shan, T. & Chen, G. Random walks on weighted networks. **87**, 10.1103/physreve.87.012112 (2013). 10.1103/PhysRevE.87.012112 23410288

[31] Lawson, A. J., Swienty-Busch, J., Géoui, T. & Evans, D. In *The Future of the History of Chemical Information* 1164 *ACS Symposium Series* Ch. 8, 127-148 (American Chemical Society, 2014).

[32] Colón-Santos S, Cooper GJT, Cronin L (2019). Taming the combinatorial explosion of the formose reaction via recursion within mineral environments. ChemSystemsChem.

[33] Greenfield SR (2020). Life and its traces in Antarctica’s McMurdo Dry Valley paleolakes: a survey of preservation. Micron.

[34] Wang, D. MTBLS1411: Hfq regulates efflux pump expression and purine metabolic pathway to increase the trimethoprim resistance in Aeromonas veronii. (2019). at https://www.ebi.ac.uk/metabolights/MTBLS1411/descriptors 10.3389/fmicb.2021.742114 PMC8652118 34899630

[35] Cronin L (2006). The imitation game—a computational chemical approach to recognizing life. Nat. Biotechnol..

[36] Cronin L, Walker SI (2016). Beyond prebiotic chemistry. Science.

[37] Kim H, Smith HB, Mathis C, Raymond J, Walker SI (2019). Universal scaling across biochemical networks on Earth. Sci. Adv..

