# Supplementary material for: Identifying molecules as biosignatures with assembly theory and mass spectrometry
Source: Nat Commun. 2021 May 24;12:3033. doi: 10.1038/s41467-021-23258-x (PMC8144626; doi:10.1038/s41467-021-23258-x)
Supplement: Supplementary file 1 — Supplementary Information [file 41467_2021_23258_MOESM1_ESM.pdf]

Supplementary Information for:

Identifying Molecules as Biosignatures with Assembly Theory and Mass Spectrometry

Stuart M. Marshall†,[1] Cole Mathis†,[1] Emma Carrick,[1] Graham Keenan,[1] Geoffrey J. T. Cooper,[1] Heather Graham,[2] Matthew Craven[1], Piotr S. Gromski,[1] Douglas G. Moore,[3] Sara. I. Walker,[3] and Leroy Cronin*[1]

[1]*School of Chemistry, University of Glasgow, UK.*

[2]*Astrobiology Analytical Laboratory, NASA Goddard Space Flight Center, USA*

[3]*Beyond Centre for Concepts in Fundamental Science, Arizona State University, USA*

Contents

1 Complexity as a Biosignature 3
2 Computing Molecular Assembly, Algorithm implementation details 4
2.1 Theory of Object Assembly 4
2.2 Molecular Assembly Index 6
3 MA in Chemical Space 11
3.1 Possible Chemical Space using MOLGEN 11
3.2 Known Chemical Space in Reaxys 13
4 Random decision tree model of molecular synthesis 14
4.1 Model Description and Parameterization 14
4.2 Results and Interpretation 15
5 MS/MS and MA 17
5.1 Analytical Decisions 18
5.1.1 Ionization source 18
5.1.2 Resolution 18
5.1.3 Collision Energy 18
5.1.4 Direction Injection and Isomeric species 18
5.2 Data collection using Orbitrap 19
6 Sample Prep Details 22
6.1 Yeast 23
6.2 Urinary Peptides 24

6.3 Rock and Soil Samples....24
6.4 Beer....24
6.5 Dipeptides....24
6.6 Formose Reaction Mixtures....24
6.7 Miller-Urey Spark-Discharge mixture....24
6.8 Whisky....24
6.9 Taxol....25
6.10 Carbonaceous chondrite (Murchison Extract)....25
6.11 Marine Sediment....25
6.12 Holocene Paleomat....25
6.13 Mid-Miocene Lakebed Sediment....25
6.14 Seawater Extract....26
6.15 Aeromonas veronii (External Data)....26
7 Table of Molecules and Associated MA values....29
8 Example Mass Spectra with Fragmentation....51
8.1 Single Molecules via SIM....51
8.2 Environmental Samples via Data Dependent Acquisition....55
9 Inferring MA with Convolutional Neural Networks....58
10 References....60

1 Complexity as a Biosignature

Biosignatures are defined as "object[s], sub-stance[s], and/or pattern[s] whose origin specifically requires a biological agent"(*1, 2*). Here we focus on evaluating the plausibility of molecular artefacts (or objects) as biosignatures. For reviews relevant to evaluating atmospheric patterns as candidate biosignatures we refer the reader to recent work by Walker et al.(*3*) and Schwieterman et al.(*4*). Previous authors have suggested using specific biomolecules, such as lipids(*5*) or nucleic acids(*6*) to identify the living systems. Unfortunately, relying on specific organic molecules prohibits us from detecting life based on non-terran biochemistry. Others have suggested using homochiral polymers as a universal biosignature(*7*), however abiotic processes are known to produce enantiomeric excesses that could result in false positives(*8*). Finally, isotopic fractionation has been posited to distinguish biologically generated material from abiotic material(*9*). However, as pointed out by Neveu(*10*) isotopic fractionation can also be generated by abiotic processes, and effective evaluation of samples requires prior knowledge of metabolic pathways, restricting its applicability to known life.

Living systems are able to generate complex molecules in a way that is not possible for abiotic systems, and so a complexity-based model is a promising prospect for use as a molecular biosignature. We propose that a good molecular complexity measure for the purpose of life detection should satisfy three criteria. Firstly, the model would need to reflect the pathway of formation of a molecule, providing a correlation with or a bound to the likelihood of overcoming the combinatoric explosion of diversity which results from random interactions, thereby providing a distinction between potentially abiotic molecules, and those that required a biological influence to form. Secondly, a good complexity measure needs to be conceptually simple and intrinsic, with minimal external choices required. We cannot take into account all of the rules of chemistry, environmental conditions, and multifaceted interactions that molecules can undergo without generating a complexity model that is too convoluted to use. Additionally, we want to avoid imposing external weightings that do not necessarily correlate consistently with likelihood of abiotic formation, such as ring counts, or the presence of specific functional groups or heteroatoms. Finally, for use in life detection, there must be a consistent experimental predictor, so that we can analyse unknown molecular samples and determine their complexity.

The determination of molecular complexity has been extensively explored theoretically, with many metrics devised based on structural, topological, or graph theoretical complexity. These include measures based on specific graph features, such as counts of atoms/bonds(*11*), distances between atoms in the molecular graph(*12, 13*), paths through the molecular graph(*14*) and total walk counts(*15*), connectivity of atoms(*16, 17*), number of subgraphs(*18*), fractal dimensions(*19*), and information theory based on molecular symmetry(*12, 20, 21*). Other complexity measures rely on weighting for specific molecular features such as the number of rings, heteroatoms, and properties such as electronegativity(*22, 23*). Complexity measures have also been proposed which use machine learning(*24, 25*), and crowdsourcing(*26*).

No molecular complexity measure proposed to date fully fulfils the criteria we propose above. There is no complexity measure that we are aware of that capture potential history of formation for molecules. Some measures incorporate intrinsic features that intuitively add complexity, such as the number of molecular fragments (subgraphs) and atom connectivity, or lower it such as symmetry, these measures do not track how likely such features are to form by bringing

fragments together one step at a time. This allows for a potentially large increase in complexity at low combinatoric cost, for example connectivity indices could increase dramatically in a single step, or symmetry could quite easily be broken in a single step, resulting in a discontinuity between the complexity measure and the effort required to increase the molecule's complexity. We can also disregard any measures that count specific features, or include current synthetic difficulty, as these are externally weighted and cannot be shown to be useful for life detection in an agnostic sense. This is also true for machine learning and crowdsourced based measures, as those models are restricted by the chemistry of life observed so far on earth, and we have no way of telling if they could be used to threshold life detection in general. Finally, we do not know of any molecular complexity model published to date that has a strong correlation with experimental data. The model proposed in this paper fulfils these three criteria, allowing for experimental analysis to indicate a complexity value based on a simple, intrinsic, agnostic measure, that can be shown in theory to bound the biological threshold.

2 Computing Molecular Assembly, Algorithm implementation details

In a previous paper(*27*), we introduced the concept of Pathway Complexity (now renamed Object Assembly) as a model by which we could determine if an object had a biological origin. Here we review the concept of Object Assembly, with a focus on its application to molecules, and introduce an algorithm used to calculate the Object Assembly Indices of molecular graphs

2.1 Theory of Object Assembly

The Object Assembly Index (OA) of an object is defined in the context of an assembly space(*28*), which defines how objects can be made from a set of basic building blocks through combination operations. Each point in the assembly space is an object, and arrows between objects A and C are labelled by another object B with the implication that A and B can be combined in some predetermined way to make object C (See Supplementary Figure 1). There is required to be a symmetric arrow in the space between objects B and C, labelled with object A. Traversal along arrows in the assembly space represents a series of joining operations. An Assembly Subspace is a subset of objects and arrows that itself constitutes an assembly space. A subspace that contains the irreducible building blocks of the parent assembly space (is "rooted"), and contains a target object X, can be thought of as containing a recipe to create X using joining operations. The OA of X is defined as the size of the smallest rooted Assembly Subspace containing X. The OA can be thought of as the minimum number of joining operations required to create X, starting from basic objects, where objects created in the initial steps can be "re-used" in subsequent joining operations. Concepts related to Assembly Spaces and the Assembly Index are formalised in Ref. (*28*).

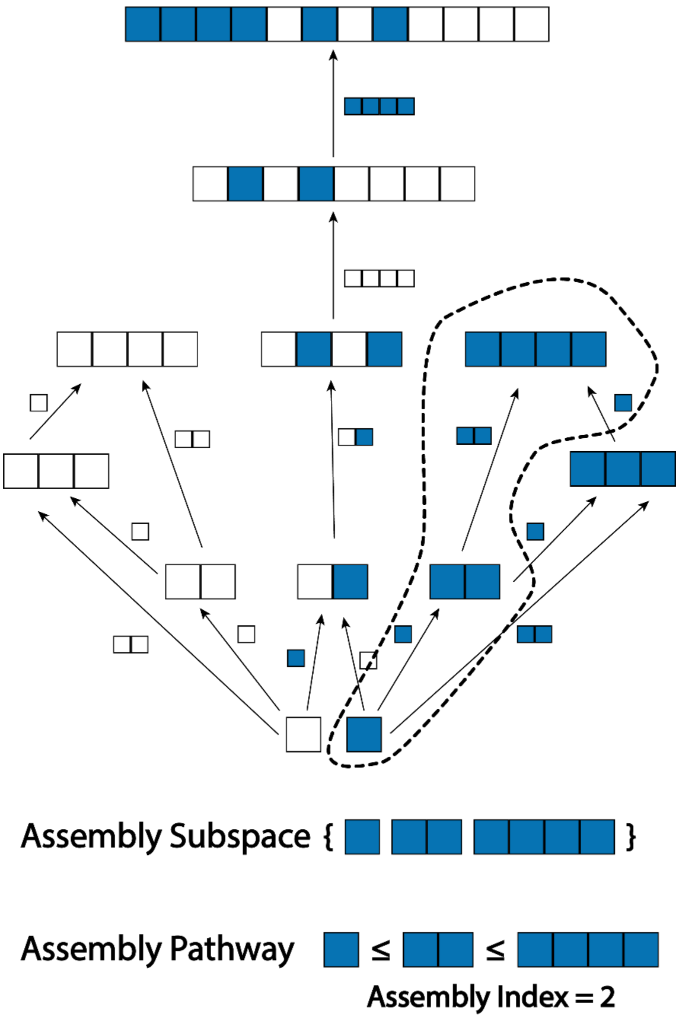

Supplementary Figure 1: An assembly space for object that can be created from blue and white blocks. Some arrows have been omitted for clarity. The label on the arrow represents the object in the space that needs to be combined with the source to make the target. The dashed region represents an assembly subspace, which is the smallest subspace that contains the object made of a row of 4 blue blocks. The assembly index of that object is the number of objects in that subspace, not including basic objects.

Intuitively, the OA of an object is correlated positively with its size, and negatively with the number of repeated and non-overlapping substructures along the minimal pathways. Any such substructures could themselves contains repeated substructures, further reducing the OA, recursively. Objects with low OA are those objects which are small and/or contain internal symmetries, while objects with high OA tend to be large and heterogenous. An upper bound for the OA of an object of size s is s-1, based on the fact that it is always possible to construct an object by adding a single basic object at each step. A lower bound can be found by considering that at each step it is possible to join the object created in the previous step to itself, and an object created this way in n steps will have size s=2^n, with n being the minimum possible OA of an object of size s. Therefore, log_2[illegible]s is a lower bound for the OA of any object of size s.

Construction of an object using the object assembly model is designed to mimic the construction of objects through random collisions starting from basic building blocks and

determining, in principle, the minimal pathway indicates how many steps this could be accomplished in. This allows us to set a lower bound on how likely the object is to be found in abundance in the context of all the other objects that could have been created through undirected/abiotic interactions.

2.2 Molecular Assembly Index

Object Assembly theory has a natural application to molecules, and the model was devised with molecules in mind. Either atoms or bonds can be considered as the irreducible objects, and typically hydrogen depleted representations would be used to reduce computational cost. In computing the Molecular Assembly (MA), we use a graph-theory based model, considering only the connectivity of atoms and bonds within molecules, restricted by valence rules. A more complex assembly space could be considered whereby atoms and bonds are represented in three dimensions, and a joining step between two structures is only permitted if the resulting structure is chemically feasible. This would be computationally prohibitive with current algorithmic implementations although it may be explored in a future study.

Assembly pathways in the model described here are not representative of molecular synthesis but rather represent what synthesis would be if the complexities of chemistry, other than valence rules, were ignored. If we consider syntheses where all steps are of the form A+B→C, where C is the only product, then the space of synthetic pathways of this type is an Assembly Subspace of the Assembly Space used in our model, containing a subset of the structures and connections between them. In our previous work(*28*), we have shown that the MA in an Assembly Subspace is an upper bound for the MA in the original space, and hence such synthetic pathways cannot be shorter. In cases where A+B→C+D, or A→C+D, the most complex product will tend to have lower MA than in the case of A+B→C, and so there will be a tendency for steps of these kinds to result in longer synthetic pathways rather than shorter ones. Since most steps in our model will not represent real synthetic steps, with synthesis being significantly more difficult than in our model, we consider the MA of a molecule to be a reasonable lower bound on the shortest synthetic pathway with atoms as starting materials. This opens the possibility that a molecule will have a relatively low MA but could only realistically be synthesised in a much larger number of steps, and hence from a life detection point of view this could result in false negatives. Our intention, however, is that the measure is robust against false positives, allowing us greater confidence in the biological origin of molecules that we find to be above the threshold. Future MA models may incorporate decomposition steps and reactions with multiple products.

The molecular assembly algorithm calculates the split-branched object assembly index, a variant of the Object Assembly Index. This variant was chosen for algorithmic simplicity(*28*). The split-branch object assembly index of a molecule is an upper bound for the MA of the molecule(*28*), although there is an offset of 1 between the variants as the initial step of the assembly index is a joining of two basic objects, whereas the initial step of the split-branch process can be thought of as laying down a single basic object. The split-branch variant can be considered intuitively as forming structures in their own separate environments, before bringing them together, and so a substructure used to create one object cannot be used in the creation of a separate object without rebuilding it. In the conventional MA measure, one can think of all the structures forming in the same environment, and such reuse would be permitted. For simplicity, in subsequent paragraphs "MA" should be understood to mean the split-branched molecular assembly index.

We calculate the MA on hydrogen depleted graphs, and use bonds as basic objects, to reduce computational complexity and allow for simpler representation of molecular fragments. The algorithm takes a molecular graph, and calculates all possible connected substructures, grouping these into fragments that are identical. This grouping is done by associating fragments with their InChI string, using the InChI API,(*29*) as the InChI string is a canonical representation of a chemical structure (i.e. each chemical structure is represented by a single unique InChI string). Following this, the algorithm searches through partitions of the molecule into non-overlapping substructures, with each unique substructure in a molecule contributing its own MA to the MA of the target, plus 1 for each time it is duplicated. The MA of the substructures is calculated recursively using the MA algorithm, unless it can be determined implicitly due to the substructure size being 3 bonds or fewer (substructures of size 1, 2, and 3 bonds have MA 1, 2, and 3 respectively).

The algorithm uses several methods to reduce the computational expense of the calculation. Only substructures duplicated at least once are considered in the partitions, with bonds not in those substructures contributing 1 each to the MA. The order of searching through partitions is based on the size and multiplicity of the repeated substructures (e.g. three substructures of size 2, and two of size 4), and minimum/maximum MA values for such partitions can be calculated based on size alone. In this way, substructures can be searched in order of increasing minimum MA, which allows the algorithm to terminate when the minimum MA of a partition based on size/multiplicity is greater than or equal to best MA value found so far. A simplified flow-chart representation of the algorithm can be seen in supplementary figures 2 - 4. Two examples of this calculation are shown in supplementary figure 5. The recursively constructed fragments are color coded, to match the number of steps they contribute, while the black bonds indicate the additional bonds required to complete the molecule.

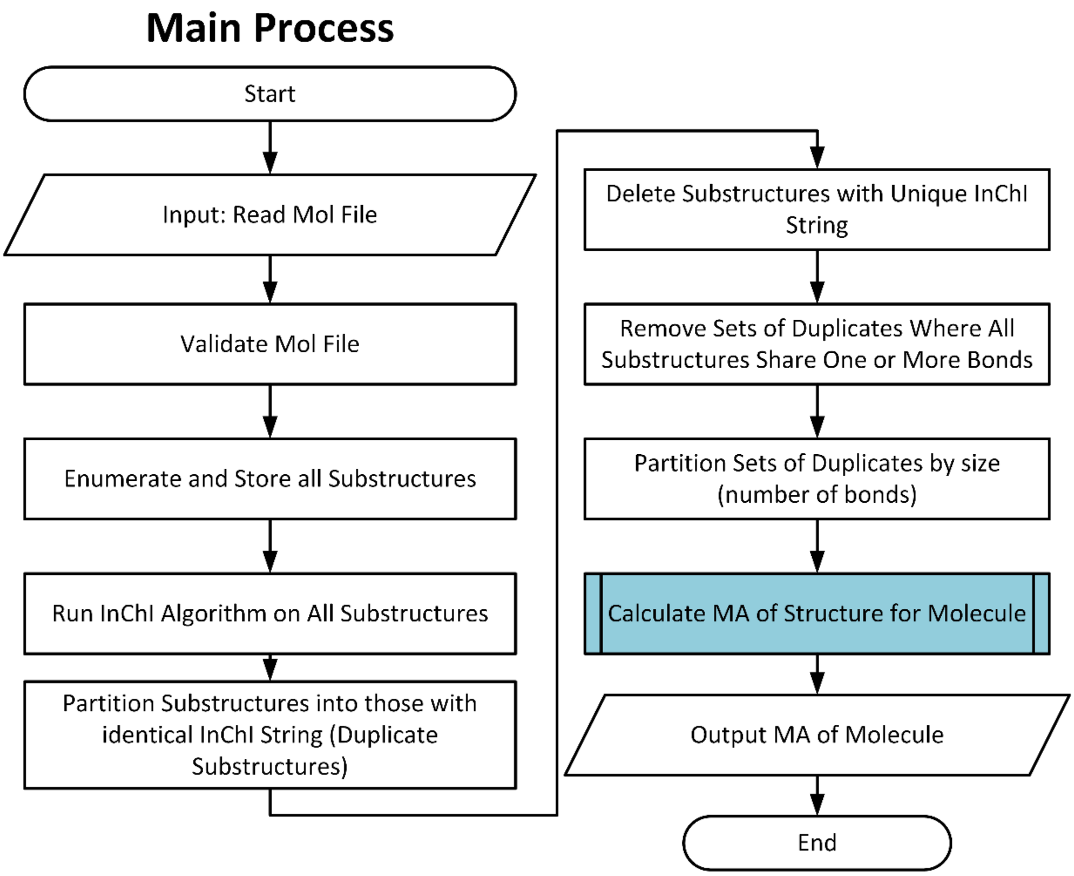

Supplementary Figure 2: Flow Diagram describing the algorithmic implementation of the Split-Branch Assembly Index calculation as applied to molecular structures. The blue highlighted box corresponds to a subprocess described with its own flow diagram.

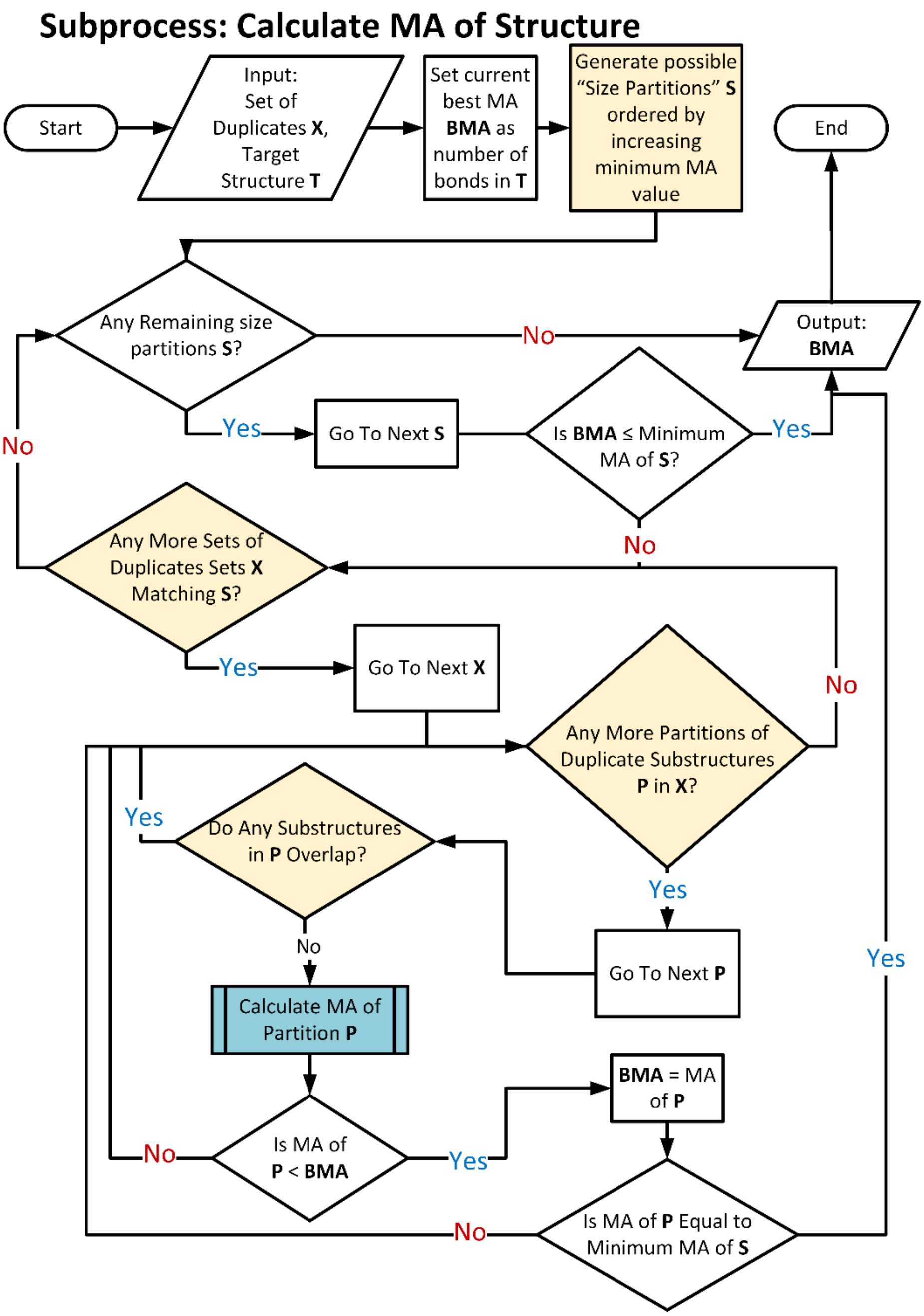

Supplementary Figure 3: Detailed Explanation of the "Calculate Substructure MA" subprocess called in the Split-Branch algorithm Method. The blue highlighted box corresponds to a subprocess described with its own flow diagram. The yellow-gold highlighted boxes are described in more detail in the SI text.

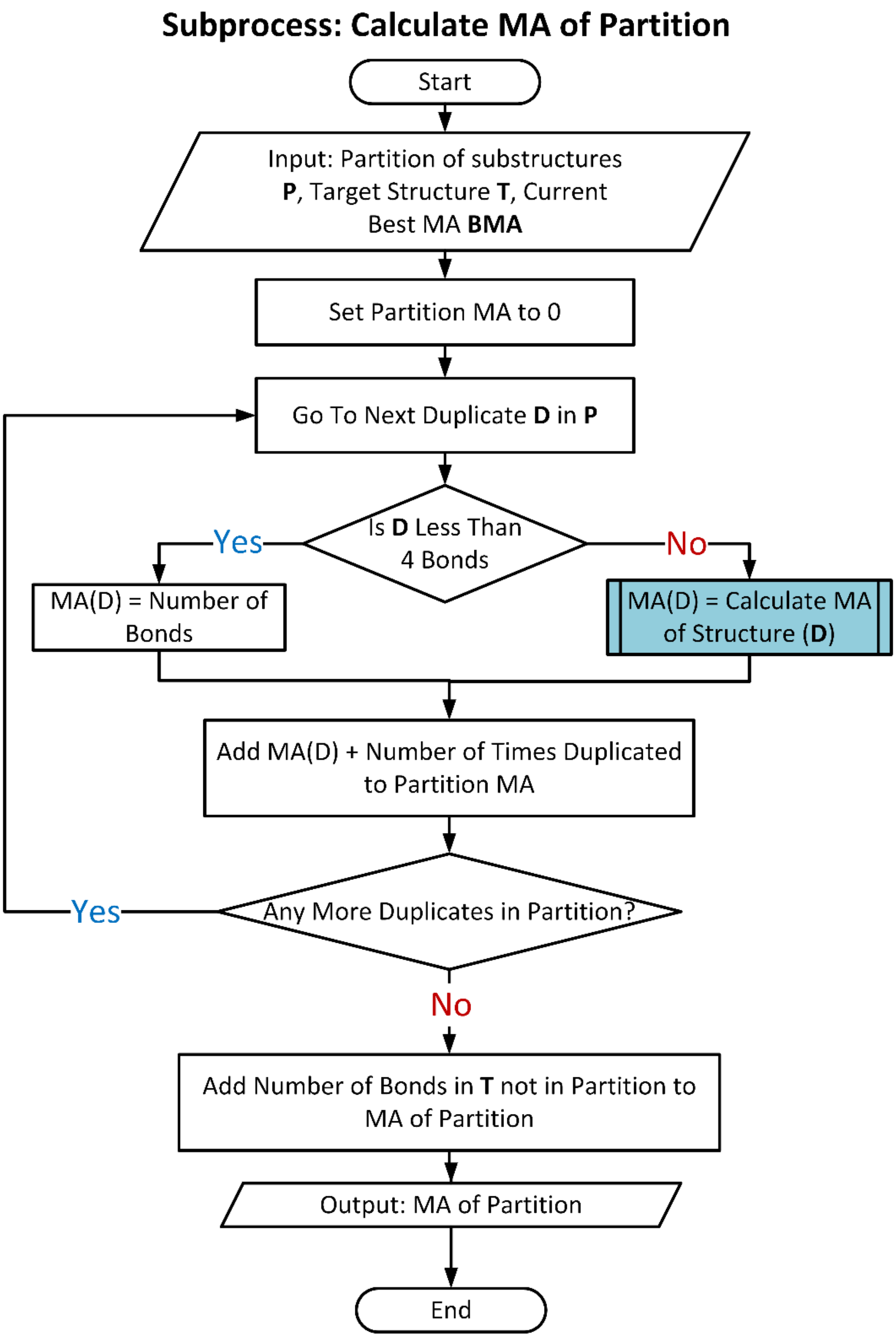

Supplementary Figure 4: Detailed Explanation of the "Calculate MA of Partition" subprocess called in the Split-Branch algorithm Method. The blue highlighted box corresponds to a subprocess described with its own flow diagram.

The algorithm to calculate the MA for molecules was written in C++, compiled with the Boost library and InChI API(*29*), and currently runs natively on Windows, or on Linux if using WINE. It takes an MDL Mol file as input, and outputs a single integer for the MA, as well as some details of the minimal length pathway found. The algorithm terminates when a single

minimal length pathway is found, so no information is provided on the number of minimal length pathways.

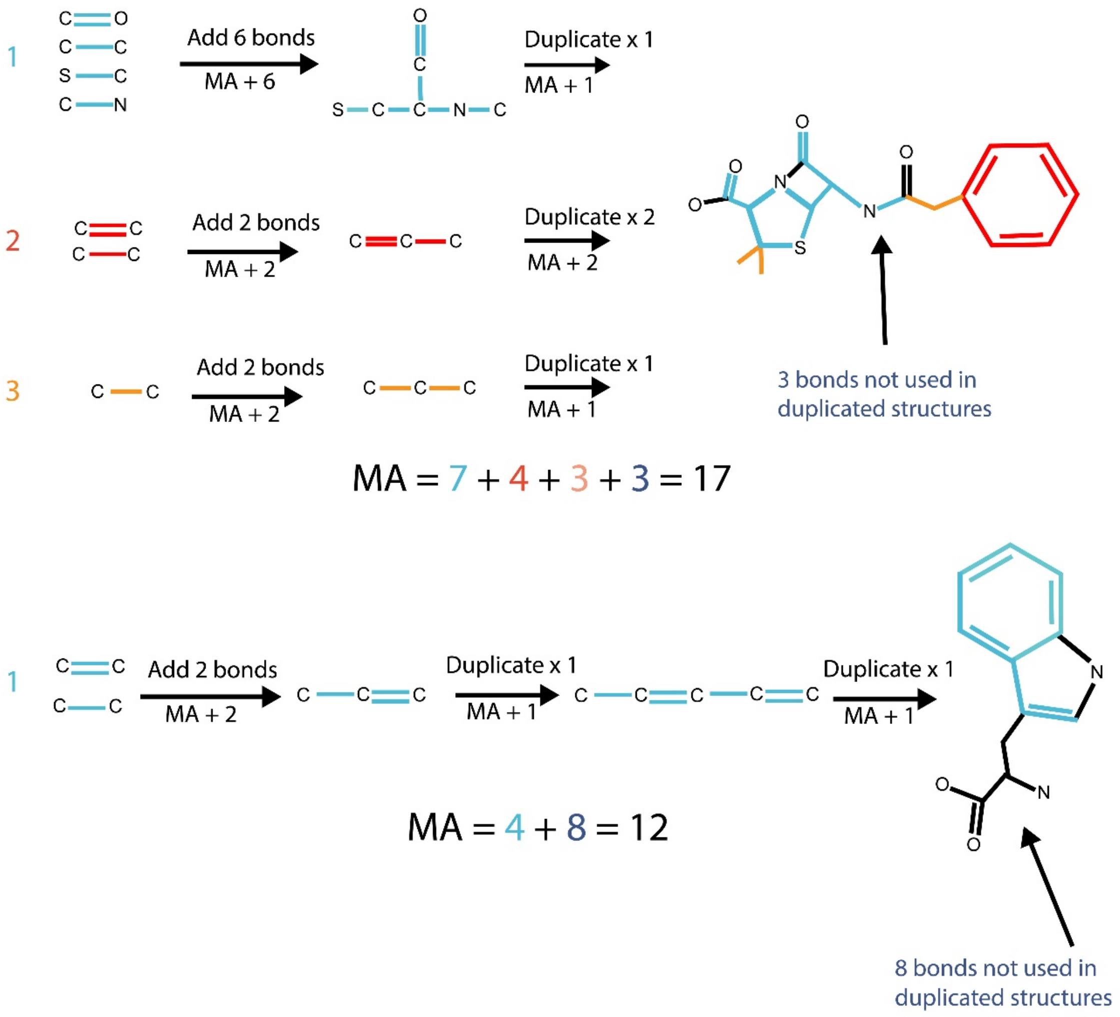

Supplementary Figure 5: Schematic examples of the Split-Branch calculation of Molecular Assembly Index for penicillin (top) and tryptophan (bottom).

3 MA in Chemical Space

In exploring chemical space, we distinguish between theoretical chemical space, being the space of all possible molecules, and extant chemical space, being the space of molecules known to have been discovered or synthesised (and documented). To explore extant (known) chemical space we utilize a subset of the Reaxys® database(*30*) with molecular weight up to 1000 Daltons, which contains approximately 25 million substances. The distribution of molecular weights from this subset is shown in Supplementary Figure 6. To explore possible (or theoretical) chemical space we generated possible chemical structures using MOLGEN(*31*).

3.1 Possible Chemical Space using MOLGEN

In order to estimate the size of a constrained subset of theoretical chemical space, we used the commercial software MOLGEN(*31*), which enumerates all structures for a given molecular

formula, or formula range. MOLGEN commands we used to enumerate molecules took the form:

$$> \text{ mgen C6H6 } -\text{v}$$

which will enumerate all structural isomers with molecular formula C6H6, or

$$> \text{ mgen C0} - \text{10S0} - \text{10N0} - \text{10O0} - \text{10H0} - \text{100 } - \text{sum C} + \text{N} + \text{O} + \text{S} = 10$$

which will enumerate all structural isomers with up to 10 atoms of each of C, N, O, and S, and with a total of 10 atoms of C, N, O, and S, and up to 100 H atoms (an arbitrary high figure chosen to represent any number of H atoms)

Initially, we enumerated all hydrocarbons with up to 12 carbon atoms, for all possible combinations of C and H atoms, see Supplementary Figure 7 (left). The number of possible structural isomers rises rapidly with the number of C atoms, peaking at C12H8 with approximately 47 million structural isomers.

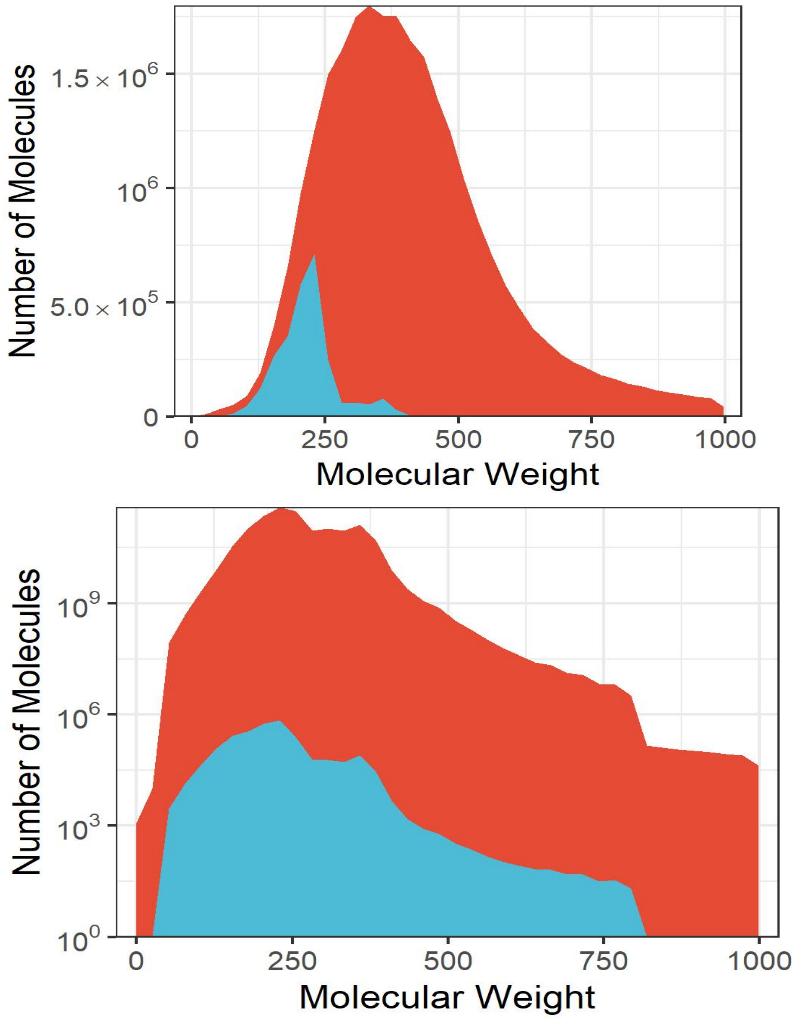

Supplementary Figure 6: Distribution of Molecular weights in Extant chemical space on a linear (top) and logarithmic (bottom) scale. The colours indicate the distribution for molecules which have Molecular Assembly indices calculated.

Next, we calculated the total number of possible structures containing only C, N, O, S, and H. We calculated up to 9 non-H atoms due to computational constraints, with the total number of structures being approximately 120 million, shown in Supplementary Figure 7 (right) The number of structures for n non-H atoms was approximately $(n + 3)!/6$, and assuming an increase at this rate would imply that the number of possible structures for 70 non-H atoms would be approximately 10^{100}, significantly higher than the estimated number of atoms in the observable universe. Conversely, the number of possible molecules in known chemical space initially increases as size increases from small molecules before dropping off as molecules become larger, less likely to be found in nature, and more difficult to synthesize. Using Reaxys as a proxy for known chemical space, the number of total molecules containing only C, N, O, S and H peaks at about 530k molecules for 24 non-H atoms, before reducing to ~12k for 70 non-H atoms, with no substances having over 82 non-H atoms.

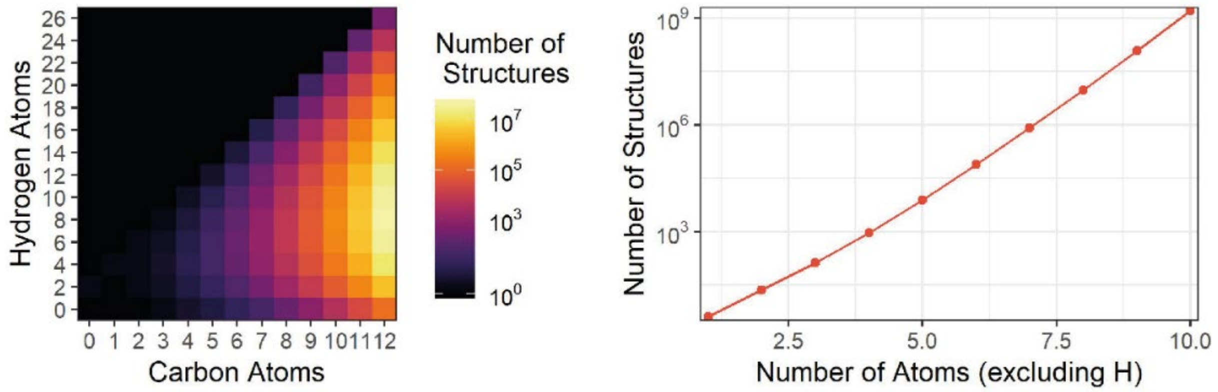

Supplementary Figure 7: Graphs of possible chemical structures as enumerated by MOLGEN. Left graph shows the total number of possible hydrocarbons up to 12 C atoms. Right graph shows the total number of non-H atoms in molecules containing up to 10 atoms of C, N, O, and S.

3.2 Known Chemical Space in Reaxys

A subset of the 25 million molecules in the Reaxys database(*30*) was analysed using the MA algorithm. The computational complexity of the algorithm prevented analysis of molecules with MA greater than approximately 27, and there is a bias towards successfully calculating molecules with lower MA value at any given molecular weight. The distribution of molecular weights for molecules with successfully computed MA is shown in Supplementary Figure 6. However, it can still be seen that the MA of molecules tends be more broadly distributed for each molecular weight above a MA of 10-15 (see Supplementary Figure 8). We interpret this spread to indicate that for small (low mass) molecules, the range of MAs for each molecular weight is tightly constrained, due to the limited number of ways small molecules can be constructed, however this effect is removed for heavier molecules. This means that above a certain mass range, the molecular weight of a molecule and its MA effectively decouple. To confirm this effect was not an artefact of the relatively low representation of high MA molecules in the data, we subsampled the data in Supplementary Figure 8, such that the molecular weight range was sampled uniformly. This subsampled data was used to generate Figure 2A in the main text which shows that MA is highly constrained by molecular weight for molecules with masses between ~1-250 Daltons.

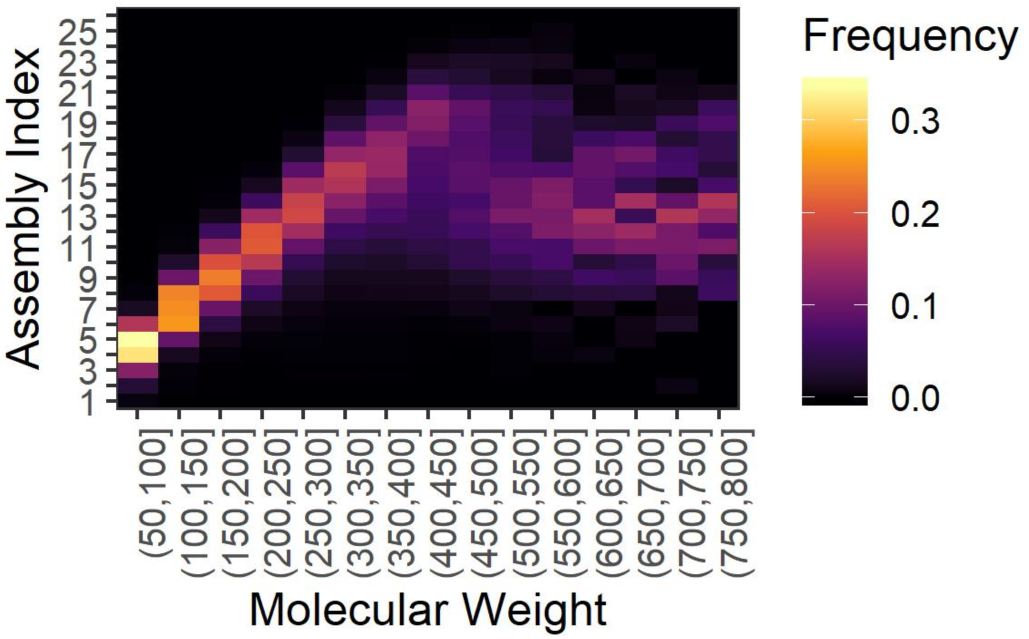

Supplementary Figure 8: 2D histogram of molecular assembly against molecular weight. Molecular weight bins are normalised so that the intensity in each bin sums to 1, as there are a far greater number of low molecular weight molecules in the database. Unlike figure 2B in the main text, this figure shows all of the calculated MA indices.

The disparity between the number of known molecules and the number of possible molecules suggests that novel ways of exploring chemical space are required to identify important molecules and processes amongst the chemical noise. Exploring the structure of chemical space using molecular assembly could help identify processes that increase chemical complexity and generate molecules for material design, drug discovery and processes critical to artificial life.

4 Random decision tree model of molecular synthesis

Our goal is to use MA to distinguish biological artefacts from abiotic chemical products. To accomplish this, we must be able to determine a threshold MA for biological artefacts above which any reliable synthesis must be due to biological processes. To estimate a range of values for this threshold we explored the statistical properties of assembly pathways by computationally modelling the molecular assembly process as a random walk on directed trees.

4.1 Model Description and Parameterization

In this model the root of the tree corresponds to abiotically available precursors, while the number of leaves on the root correspond to the number of possible combinations of those precursors. Each node in the tree (besides the root) corresponds to molecules which could be synthesized from the abiotically available precursors. The depth of a given node (the shortest number of steps between it and the root) corresponds to the MA of that compound, with those precursors, see Figure 1B in the main text. We label the breadth of the tree at depth i as k_i.

Using this model, we can calculate the likelihood of different assembly pathways and determine a MA which corresponds to a sufficiently low likelihood of spontaneous formation. These likelihoods will depend on the properties of the decision tree used. For example, if we assume that the breadth of the tree is constant, k, for all depths, and that for each node all leaves

are equally likely then the likelihood of any molecule with a MA index of m, will be $p(m) = k^{-m}$. These two assumptions, that the number of leaves (and therefore number of possible reactions) for any given molecule is constant and that all leaves are equally likely, are unrealistic for most chemical systems. Organic molecules can interact to form many different products. Which product forms depends on their structural and thermodynamic properties. Likelihood of those products can vary dramatically, often spanning several orders of magnitude. We relax these assumptions and compute bounds on the likelihood of the most likely assembly pathway in the decision tree. We introduce two parameters, described below, which control the properties of the tree. The first parameter h, controls to relative likelihood of possible transitions. The other parameter is a function, which controls the expected number of possible transitions at each point in the synthesis process, we test a linear function and an exponential function as two examples with different behaviour.

4.2 Results and Interpretation

Our goal is to determine the probability of the most likely pathway through a decision tree. To provide robust estimates in what follows we simulated 1 million decision trees under different assumptions, and for each tree we calculated the most likely pathway. Given this distribution of likelihoods of for each set of conditions, we report a conservatively high probability by using the 99th percentile of the distribution.

We first relaxed the assumption that all leaves of a given node are equally likely. The simplest way to do this is to assume that the likelihood of each choice is drawn from a uniform random distribution between 0 and 1 and normalized such that the sum of the likelihoods is one. Under these conditions we find that the resulting probability does not change significantly from the case where all choices are equally likely, following the trend of $p(m) = k^{-m}$. The uniform random distribution over choices implies that all future choices have a likelihood that are of the same order of magnitude, in this sense the distribution over choices is very homogenous. We next investigated more heterogenous distributions.

An obvious way to generate distributions over choices which vary by orders of magnitude is to first draw values, x, from a uniform distribution between 0 and the value h, and then assigning the likelihood of choices as 10^x (where the likelihood of all choices is once again normalized to one). Example of distributions generated using this method are shown in Supplementary Figure 9. By varying the value of h we can investigate more or less heterogenous distributions. Under these conditions we find that the more heterogeneous the distribution the more likely the most likely path through the tree way. This is an unsurprising result; the effect of very heterogeneous distributions is to funnel the probability towards a limited subset of all possible paths at each step. Given these findings we continued to use the heterogenous distributions with $h = 4$ for the remaining simulations, which we believe captures the appropriate degree of bias.

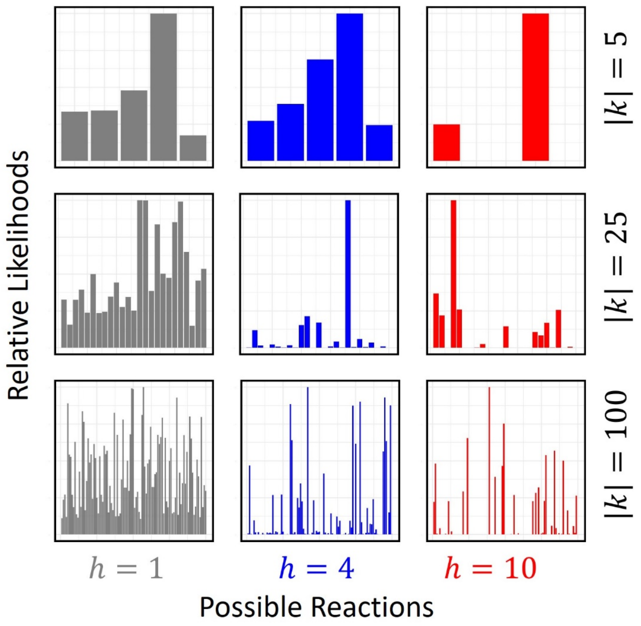

Supplementary Figure 9: Choice Distributions for various number of choices (k) and heterogeneity (h) values.

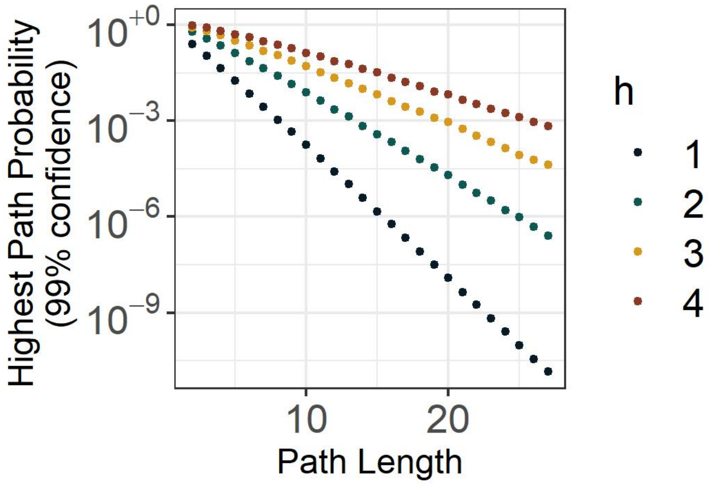

Supplementary Figure 10: Highest Path Probability vs Path Length using a uniform and constant number of choices at each depth. Different colours correspond to different choice distribution heterogeneities. Examples of choice distributions for each heterogeneity values are shown in Supplementary Figure 9.

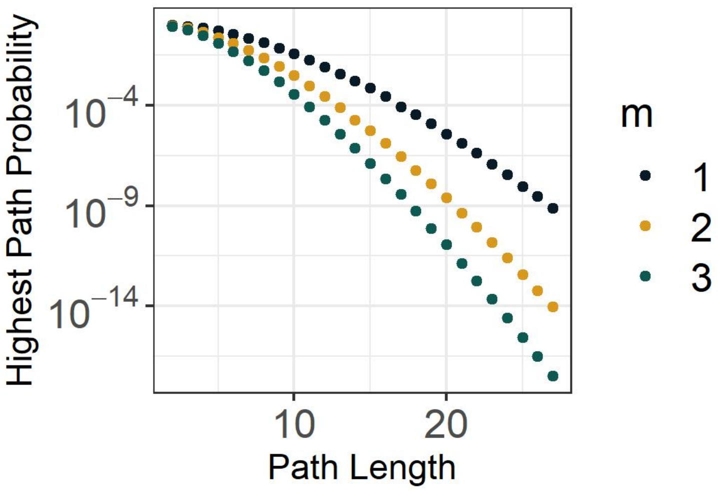

Supplementary Figure 11: Highest Path Probability vs Path length using a linearly increasing number of choices at each depth. Different colours show different slopes for the linear function. The heterogeneity of the choice distribution was kept constant at $h = 4$.

We can further relax the assumption that the number of leaves at a given depth is constant. First, we considered the case where the number of choices at a given depth is a random integer from the uniform distribution between (2,26). This did not significantly alter the results of the previous simulations. In general, we expect that the number of choices to grow as a function of the depth of the node. We model this growth in two ways, with a linear function of slope (m) and a power law with an exponent (α). For the linear case with a slope of 3 we find that molecules with a MA index of 30 have a likelihood of 1 in a mole (10^{-23}), while in the power law case with an exponent of 3, molecules of MA index 15 have a similar likelihood, which is shown the main text Figure 1C. Given these computational results we suggest that an appropriate threshold for MA is likely to be within the range of 15-30.

Implicit in this model we have assumed that all precursors and intermediate structures are not only available but nearly infinite in concentration. This is a generous approximation. Relaxing this assumption would only serve to universally decrease the probabilities calculated here, which means that our model is overestimating the likelihood of the most likely pathway. We do not make any assumptions about the effect of atomic composition or bond stability in the model presented here. This means that the results generalize beyond known biochemistry to any assembly process that can be presented by the recursive joining of structures.

5 MS/MS and MA

In order to identify molecules which are produced via biological processes we need a method to experimentally determine the Molecular Assembly Index of any compound. We chose to investigate MS/MS as an analytical method to determine the MA of molecules based on the hypothesis that the fragmentation pattern of molecules should be closely related to the recursive decomposition used to determine the MA of the molecular graphs. Here we describe the analytical procedure we developed to experimentally explore the relationship between fragmentation and MA of molecules.

5.1 Analytical Decisions

5.1.1 Ionization source

In the work presented here we chose to analyse all of our samples using an Electrospray Ion source for the mass spectrometry (ESI). There are of course different ionization methods that could affect the analysis. ESI was chosen in this case because it is a standard ionization method for most omics-based approaches and in the analysis of complex mixtures (REF). The most significant difference between ESI used here and other sources is that some molecules would be more readily ionized using different sources. Our analysis of over 100 compounds suggests that many molecules, particularly small organics ionize well enough for detection using ESI. In the context of life detection experiments future work could be done to test different ionization methods based on mission objectives and predictions about the extra-terrestrial conditions from which samples will be collected.

5.1.2 Resolution

It is important we do not make any assumptions about what we are looking for in terms of elemental composition. We are just concerned with the intrinsic complexity of the molecule as determined by the fragmentation. Our technique does not need to identify any elementary composition – the MA maps as a function of the number of fragments. Therefore, the resolution is only set to ensure we can select a single nominal mass for fragmentation. This a key point of our approach meaning it can search for highly complex and unknown molecules.

5.1.3 Collision Energy

In routine bioanalysis of a particular analyte it would be proper to fully optimize the instrumentation for the analyte of interest. We investigated the effect of collision energies on the known molecules selected for our standard curve using both 35 and 45 kV. As expected at higher collisions there are more MS2 product ions, although across all the molecules tested the correlation between MA and MS2 product ions was similar. We decided to cautiously use 35 kV for the environmental because some molecules not able to fragment completely. In the environmental samples this would lead to false negatives which we have already accepted as part of our approach.

5.1.4 Direction Injection and Isomeric species

Using direction injection makes our experiments more directly comparable to potential space missions. However, it also raises the possibility of co-fragmentation of isomeric species potentially leading to overestimates of the number of fragments associated with any one ion. In order to test for this, we reanalysed one sample that is notoriously complex from an analytical perspective, the Murchison meteorite sample. We reanalysed this sample using LC-MS. However, because this analysis was done for a slightly different set of experiments, it was run with a higher mass range, to compare it to the results presented here only parent masses in the 300-500 m/z range were kept. The results of this analysis identified multiple isomeric parent masses that would have been observed simultaneously in the direct injection sample, however the final analysis demonstrated the LC-MS results were consistent with our direct injection results (see SI Figure 20).

The LCMS procedure is as follows: analyte was infused into the mass spectrometer using a Thermo Heated ESI (H-ESI) source with a +3.8kV voltage applied. The mass spectrometer was run with a DDA method, with a mass range of 150-1750 *m/z*. The 20 most intense ions were selected for fragmentation with dynamic exclusion with ions excluded for 30 seconds if

detected twice within 30 seconds. The 30 second period was chosen to match the expected LC peak width. HCD fragmentation was set at a fixed 35%. Separation was achieved using an EC-Poroshell C18 reverse-phase column (dimensions: 150 mm x 4.6 mm, pore size: 2.7 µM). A gradient method was applied at a fixed total flow rate of 0.5 ml min^{-1}. Total run time was 55 minutes. The timetable for the gradient method is below:

Time (min)	% A	% B
0	80	20
15	60	40
25	5	95
52.9	5	95
53.00	80	20

5.2 Data collection using Orbitrap

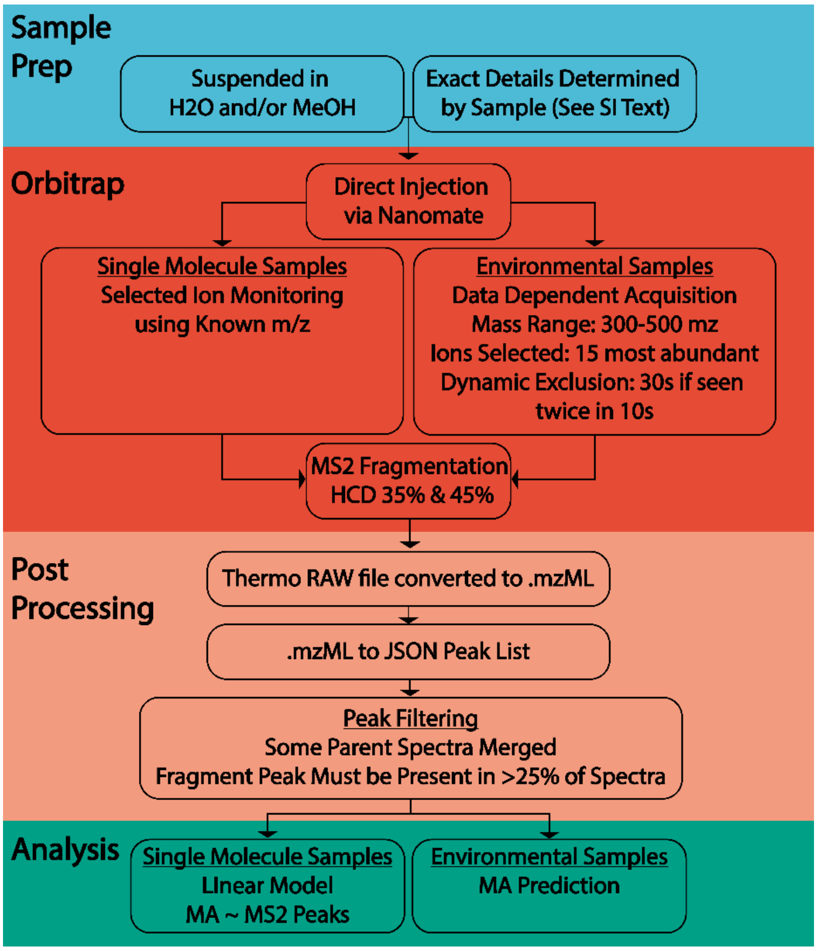

Supplementary Figure 12: Similar preparation procedures were applied to all the samples, slight variations in sample preparation were used due to the nature of different samples, these differences are documented in the text below. Prepared samples underwent identical analysis with a 15µl injection from an Advion Nanomate, followed by an MS1 full scan and MS2 fragmentation on a Thermo Fusion Lumos Orbitrap. The MS data was then processed and analysed.

Samples were analysed using a Thermo Fusion Lumos LTW-Orbitrap, which is capable of multiple rounds of fragmentation at a high resolution when ions are scanned in the Orbitrap after HCD fragmentation. By comparing data from fragmented molecules to the calculated MA of the associated molecular graph, we were able to uncover a correlation between MS fragmentation data and MA, allowing us to experimentally determine the MA of unknown environmental samples.

Molecules were solubilised in MeOH:H_2O as much as possible and introduced to the Thermo Fusion Lumos Tribrid Orbitrap mass spectrometer via an Advion Nanomate. 15 µL of sample was injected onto an emitter with a +1.2 kV voltage applied. Samples were analysed for 6 mins, during which a Single Ion Monitoring (SIM) scan was performed for the specific molecule's m/z, followed by MS2 fragmentation. Both the SIM and MS2 fragment ions were analysed in the Orbitrap with HCD fragmentation set at 35% and 45%. The isolation window was set at 0.5 Da, the resolution of the SIM scan was 240000, and the MS2 resolution was 30000.

MS data was converted into mzML files using MS Convert(*32*). In-house scripts were then used to convert the mzML files into Json peak lists, with all MS1 peaks collected for each m/z over the 6 minutes analysis being merged. Spectra with maximum intensity under 50000 were discarded, and for those remaining all peaks within 0.01 Daltons were merged. All MS2 peaks not present in at least 25% of MS2 spectra from the corresponding MS1 parent were disregarded. Since our theoretical measure does not include hydrogen atoms, any peak that was +/- 1.0 Dalton from another peak was merged, thus reducing the over count of ions which differ only by one hydrogen atom. The remaining MS2 peaks were counted, and this number was used with the calculated MA of the molecular graph associated with the MS1 peak to generate the correlation.

Environmental samples were each analysed under the same ionisation conditions, without any chromatography or other separation techniques. However, the mass spectrometer was run with a Data Dependent Acquisition (DDA) method which fragmented the 15 most intense ions, using a dynamic exclusion of 30 secs if the analyte was present twice in 10 secs, with a mass range of 300-500 m/z. Given the number of ions in the complex environmental samples, we also filtered peaks from the MS2 spectra that were below 10% of the highest peak in that spectrum. All other parameters were as above. In the analysis of the complex environmental samples it was noticed that despite the high resolution of the mass spectrometer, co-fragmentation was resulting in excessively high numbers of MS2 peaks, by effectively merging different MS1 parent ions into the same MS2 spectra. To account for this phenomenon, the analysis method was amended. After counting the number of MS2 peaks for each selected parent ion, the algorithm checked the MS1 spectra for peaks within 0.5 Daltons of the parent ion. The total number of MS2 peaks was divided by the number of MS1 peaks found within 0.5 Daltons of the parent mass. This effectively accounts for the co-fragmentation patterns in that it divides the number of MS2 peaks across the total number of identified unique ions in the collision cell during the fragmentation. This method was used in all samples and was found not to affect the previous results for single ions.

In principle isomeric compounds with the same formulae could both enter the collision cell simultaneously, this is an important possibility because if they generated two distinct fragmentation spectra that were super imposed it would create more peaks than expected, potentially causing false positives using our method. Our analysis addresses this issue in two

ways. First, the chance of two completely different structural isomers having abundances at a similar order of magnitude is very small, therefore the 10% intensity threshold will cut out any fragment ions from isomers which have an abundance approximately one order of magnitude less than the dominate ion. Second different structural isomers will have different bonding energies, and the degree of these differences will be critical. Molecules with very similar bonding energies are likely to share more structural motifs, which would generate the same fragments. The fragmentation of two (or more) molecules with very different bonding energies will occur unreliably due to a random distribution of the energy between different ions. By enforcing peaks occur in 25% or more of the scans we remove those fragments which occur unreliably.

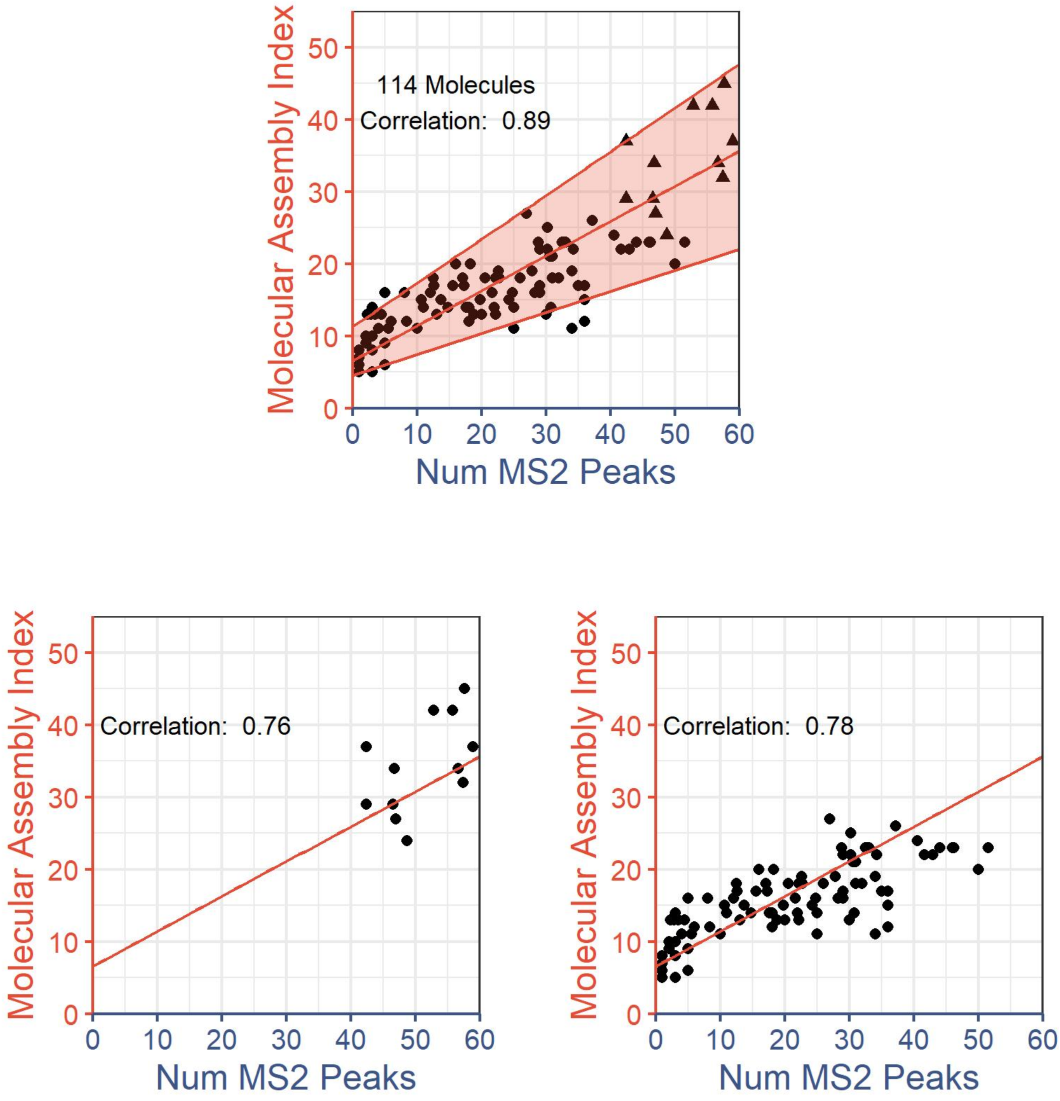

Supplementary Figure 13: Correlation of known molecular assembly indices with the number of peaks observed in MS2. Molecules were analysed multiple times and the number of MS2 peaks was averaged for each unique molecule. Results were plotted and a linear relationship

was fitted. Top shows a combined plot with all molecules. Bottom left shows the plot and correlation for only peptides, and the bottom right shows the plot and correlation for only small organics.

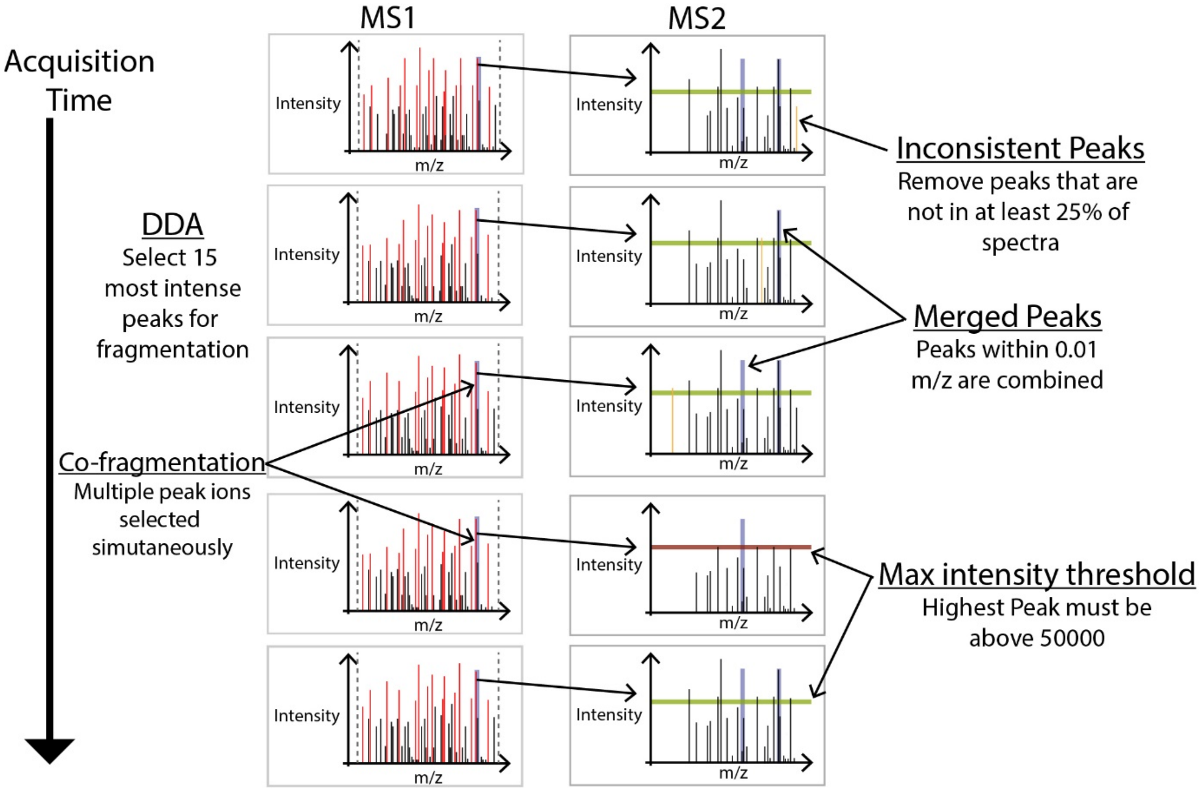

Supplementary Figure 14: Mass Spec Thresholding Procedure for MS1 and MS2 data using Data Dependent Acquisition. This workflow shows the different thresholding procedures used on the environmental samples. In the MS1 spectra the top 15 most intense peaks were selected for fragmentation. Those peaks were checked for co-fragmentation and the number of ions within the selected window was recorded. For each selected MS1 parent ion the MS2 spectra were filtered to remove bad fragmentation (using the maximum intensity threshold), peaks were merged if they were within 0.01 Dalton of one another, and any MS2 peaks which occurred in less than 25% of spectra were removed.

6 Sample Prep Details

Having established our ability to experimentally determine the MA of molecules using tandem MS, we next sought to directly test our hypothesis that high MA molecules can only be produced by living systems. To do that we prepared and analyzed several mixtures, including those sourced from abiotic, live biological, and dead/degraded biological sources. Each sample was prepared with a similar procedure with the only significant differences arising due to the different nature of the samples. The details regarding the preparation of those samples are listed below.

6.1 Yeast

A solution of sucrose was added to 1g of commercially available baker's yeast and allowed to activate at room temperature (18 ºC) overnight. On observation of carbon dioxide bubbles, the yeast was centrifuged at 15115 x g for 10 mins. The supernatant was discarded, and the pellet was split into 4 samples. One sample was labelled native and 1 mL of methanol was added followed by 30 mins sonication. The other three samples were analysed by Thermo gravimetric analysis (TGA) at three different temperatures 200 ºC, 400 ºC and 600 ºC (Supplementary Figure 15). The charred samples were then extracted by sonicating for 30 mins in methanol, and all four samples were filtered prior to mass spec analysis as described in Supplementary Information 5.2.

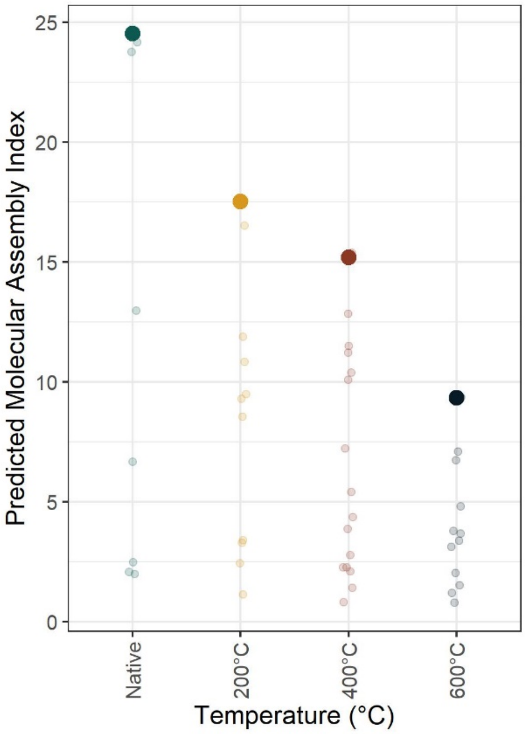

Supplementary Figure 15: Predicted molecular assembly index of yeast treated at different temperatures. Native = 18ºC, 200 ºC, 400 ºC and 600 ºC. The Predicted Molecular Assembly index can be seen to decrease at higher temperatures.

Escherichia Coli MG1655 was purchased from DSMZ (Germany). Bacteria cells were grown overnight in a 50 mL lysogeny broth (LB) media at 37 °C and 250 rpm until O.D. (optical density) of 0.6 was achieved. A 5:100 dilution in fresh media was incubated overnight at 3°C and 250 rpm and harvested when O.D. was 1.8-2.0. Bacterial culture was then centrifuged for 10 minutes at 4°C to form a cells pellet which was washed twice with 50-100 mL of ice-cold water. After that, the wet pellet was dissolved in water to make a final concentration of ca. 1 g/mL. Mechanical cell lysis using bead beating method was used to avoid any chemical or enzymatic interference. In a beat beating tube, 500 µL of cell solution was mixed with 500 µL of water and was run on the beat beater machine for 30 seconds followed by incubation on ice for another 30 seconds. This process was repeated 10 times. Samples were centrifuged at 4 °C for 3 min before removing the supernatant and centrifuging the supernatant again for 60

minutes. The resulted supernatant was collected and stored at −80 °C for further analysis, we were able to use a fraction of this lysate originally intended for other studies.

6.2 Urinary Peptides

Pooled human urine was mixed 50:50 with 2M urea, 10mM NH4OH and 0.02% sodium dodecyl sulfate. Samples were filtered with Centristat, 20 kDa cutoff, (Satorius, Göttingen, Germany). The filtrate was desalted in a PD-10 column (GE Healthcare Bio Sciences, Uppsala, Sweden). The processed urine was dried and stored at 4 °C before use. The Urinary Peptides sample was reconstituted in 500 µL H2O before injection into the mass spectrometer.

6.3 Rock and Soil Samples

Coal, serpentine, sandstone, limestone, granite, quartz and clay were separately crushed in a rock crusher and sieved to <0.25 mm. Rocks were supplied by Richard Tayler Minerals (Surrey UK). 1Mg of rock dust was submerged in 1mL of MeOH overnight at room temperature, centrifuged at 15115 x g for 10 mins and the resultant supernatant removed and filtered through Wattman paper. The eluent was loaded onto a 96 well plate and analysed by mass spectrometry.

6.4 Beer

Home brewed beer courtesy of Dr James Ward Taylor was mixed 50:50 with MeOH. Samples were then loaded onto a 96 well plate and injected into the mass spectrometer.

6.5 Dipeptides

1 mg of Alanine/Arginine (Dipeptide 1) and Glycine/Arginine (Dipeptide 2) were weighed and reconstituted in equal parts MeOH:H2O. These dipeptides were loaded onto a 96 well plate and injected into the mass spectrometer.

6.6 Formose Reaction Mixtures

The Formose Reaction(*33*) was carried out by adding Formaldehyde (0.5 mL), Glycolaldehyde (0.0126 g), Water (4.5 mL) and Calcium Hydroxide (0.0705g) to a 22mL borosilicate glass vial. The mixture was stirred at 1200rpm with a magnetic stirrer and heated at 50°C for 48 hours. Three types of experiments were done. 1) A typical one-pot control and 2) the reaction in the presence of mineral samples (see ref (*33*)).

6.7 Miller-Urey Spark-Discharge mixture

A typical Miller-Urey Spark-Discharge experiment(*34*) was carried out in the following fashion: 400mL of HPLC water was placed in the reaction flask, which was degassed, evacuated and then pressurized to 1 atm with a gas mixture of 40% methane, 40% ammonia and 20% hydrogen. The reactor was heated and a 24 kV spark discharge was turned applied, in a 10 seconds alternating ("on" - "off") duty-cycle. The experiment was continued for 7 days, after which the system was flushed with nitrogen gas and the product mixture was removed.

6.8 Whisky

Whisky was kindly donated from Group members and The Jar Troon Whisky Specialists. . A sample of a 10 year old Ardberg and a 25 year old Glengoyne were diluted 1:50 with LC-MS grade H2O before loaded onto a 96 well plate and injected and analysed using the same methods as previously described.

6.9 Taxol

Taxol (Paclitaxel) was purchased from Sigma (Cat :T7402, Lot#MKBZ4464V) and solubilised in MeOH to a concentration of 1.5mg/ml. This concentration was injected into the mass spectrometer.

6.10 Carbonaceous chondrite (Murchison Extract)

In order to test the MA of an extra-terrestrial sample we used a portion of the Murchison meteorite originally from the Chicago Field Museum. This meteorite had been kept stored in a 600 mL parafilm-covered glass beaker and sealed for an unknown period of time (many years) inside a glass desiccator containing both P4O10 and CaCl2 desiccants at the University of Chicago. Recent analyses (*35*) have revealed contamination such that these results are not pristine, however this sample offers a unique natural sample for the assessment of novel analytical procedures.

For the purposes of method development a ~4 g portion of the Chicago Murchison sample was powdered in an ashed ceramic mortar and then extracted first with methanol three times (10 mL each) and then by dichloromethane (two times, 10 mL each) by ultrasonication for 30 min at ambient temperature (*36*). To remove excess sulphur species, the solvent extracts were combined over copper pellets that had been freshly treated with 0.1M HCl (to remove CuO) then washed with water, methanol, and dichloromethane. The total volume was gently reduced by 80% by a spinning band column at 40º C before OrbiTrap analysis by direct infusion.

6.11 Marine Sediment

A standard reference material (SRM 1941b) for Organics in Marine Sediment was obtained from the National Institute for Standards and Technology (U.S. Department of Commerce, Gaithersbury, MD). These sediments were collected from the Chesapeake bay (39º 12.3'N and 76º 31.4'W) and freeze-dried, sieved to 150 µm, homogenized and then radiation sterilized by 60Co before dispersal. This SRM is intended for evaluation of methods for the analysis of polycylic aromatic hydrocarbons, polychlorinated biphenyl congeners and chlorinated pesticides, among other similar contaminants. This SRM has been thoroughly characterized with analyses published widely ((*37, 38*) among others). Extraction of this sample followed as above (SI-6.10) except that solvent reduction was achieved by gentle nitrogen blow-down at ambient temperature.

6.12 Holocene Paleomat

This sample was collected in 2016 from the upshore sediments of Lake Vanda (77º 31.2'S, 161º 38.3'E) in Antarctica(*39*). This paleomat was excavated from beneath ~5cm of sediment using ashed copper utensils and collected into sterile cryotubes and placed directly into a charged liquid nitrogen cryoshipper. Paleomats of this type are thin, desiccated remnants of ancient benthic microbial mats. The paleomat record in the Lake Vanda valley date to millennial time-scales spanning ~30,000 years and represent one of the few sources of organic carbon in local soils. Extraction of 2.0 mg of this sample followed as above (SI-6.10) except that the sulphur capture step was omitted.

6.13 Mid-Miocene Lakebed Sediment

This sample was collected in 2016 from a small basin near Mount Boreas in the western Olympus Range (77º 28.42'S, 161º 10.2'E) in Antarctica(*40*). These unconsolidated, planar

lacustrine beds contain mixed fossiliferous mat and individual samples were excavated from fine sands just beneath the surface of a rocky terrain. Sediments contained obvious fragments of fossilized moss material and have also been found to contain benthic diatoms and ostracodes as well as biomarkers from typical lacustrine microbial communities. Samples were collected using ashed copper utensils and collected into sterile cryotubes and placed directly into a charged liquid nitrogen cryoshipper. $^{40}Ar/^{39}Ar$ dating of in situ ashfall layers indicate an age 14.07 +/- 0.05 Ma. Diatom stratigraphy indicates that this lake persisted for perhaps thousands of years before burial by washed material from nearby Mt Boreas. Extraction of 2.0 mg of this sample followed as above (SI-6.10) except that the sulphur capture step was omitted.

6.14 Seawater Extract

100 ml of deep-sea water was evaporated in a round bottom flask. The remaining 'salt crust' was resolubilized in 5ml of 100% methanol. This was left on the bench for 2 hours to settle, after which 250ul of the supernatant was centrifuged at 15115 x g for 15 mins. The supernatant was then collected and 20ul was injected on to a Vanquish UPLC with the Thermo Fusion Lumos connected.

6.15 Aeromonas veronii (External Data)

Mass Spectra from a sample of Aeromonas veronii data was downloaded from Metabolights(*41*). This data was analysed on a Triple TOF 6600 (Sciex). The downloaded .wiff files were converted using MSConvert to mzML, where an index was written, and 64-bit binary encoding precision was selected. In addition, charged states 1-4 was included and MS1 and MS2 levels were selected. We then processed the data through our analysis pipeline, with the addition step of only selecting ions in the 300-500 m/z mass range. This data was collected via LC-MS/MS and therefore is slightly different than the data collected by our instrument. Accordingly, we've only selected the top 15 highest MA peaks from this data to include in the analysis shown in the main text. All the data is shown below.

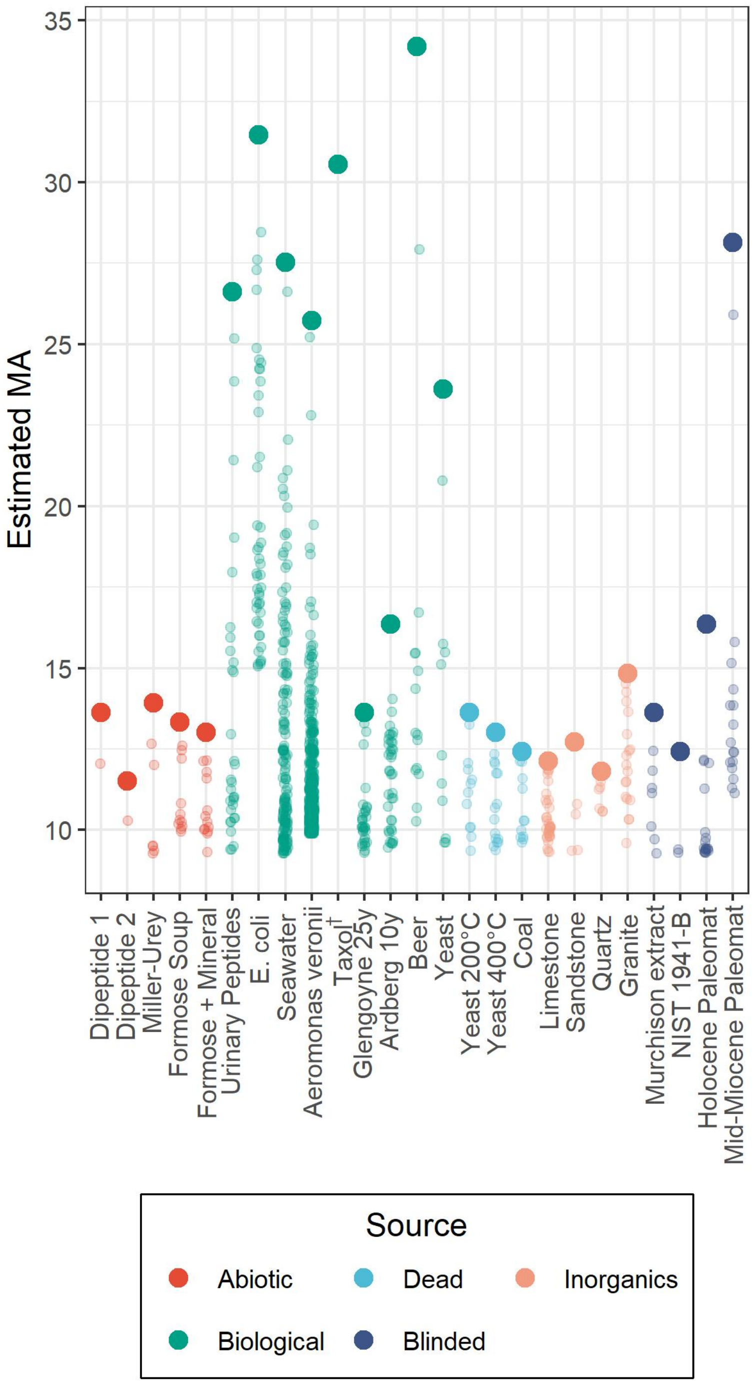

Supplementary Figure 16: All Samples including all the data from the Aeromonas Veronii Sample that was collected from an online repository (*41*) and the seawater which was run with a column attached.

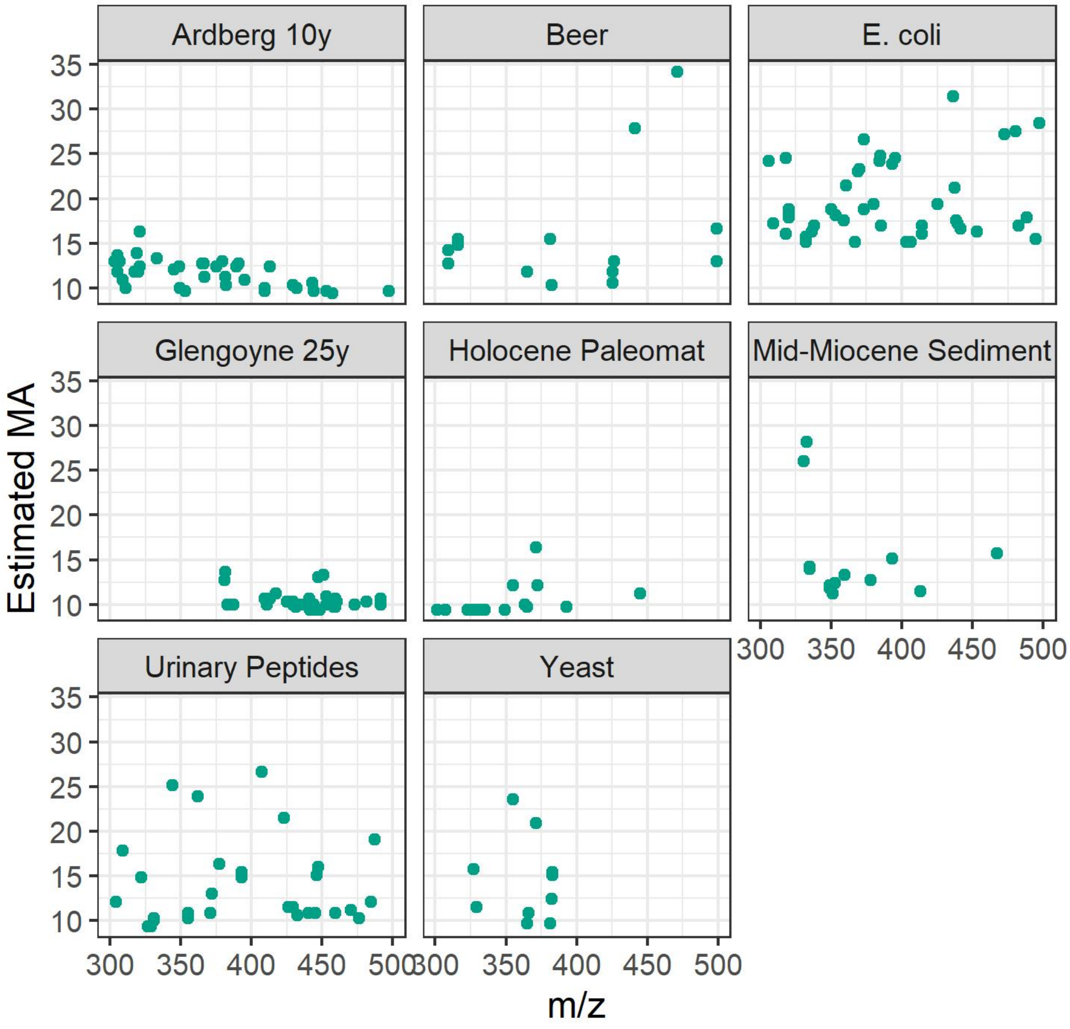

Supplementary Figure 17: MA vs m/z for all biological samples collected via our instrument.

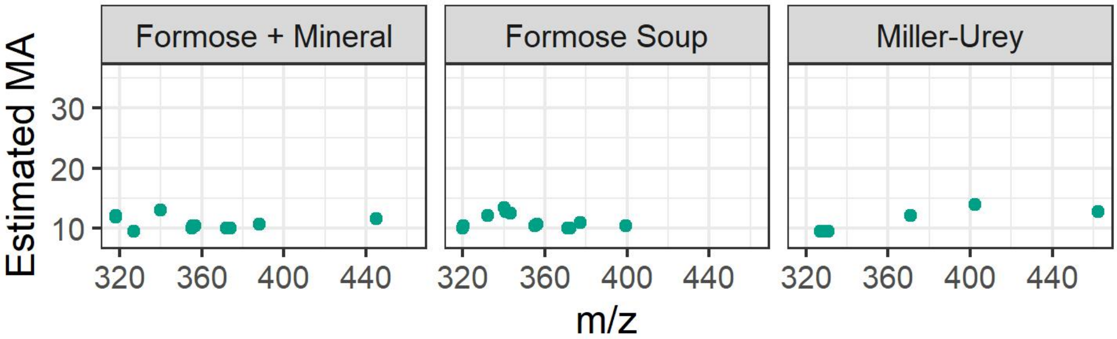

Supplementary Figure 18: MA vs m/z for all prebiotic samples.

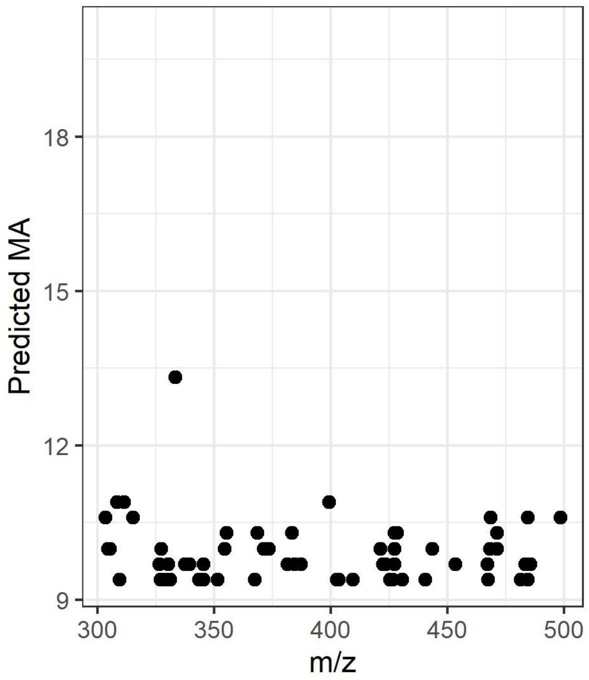

Supplementary Figure 19. MA measurements from LC-MS analysis of the Murchison Meteorite data. This demonstrate the analysis is robust to co-fragmentation because the results of the MA measurements are not affected when chromatography is used to separate the sample, indicating the Direct Injection analysis did not cause issues with simultaneous fragmentation of ions.

7 Table of Molecules and Associated MA values

For reference we've compiled a table of the 114 compounds used to correlate their MA values with associated MS2 peaks obtained by mass spectrometry. This table includes many well-known organic molecules and a set of peptides. Full details including molecular identifiers are available on demand.

Peptide: YWHQNWYF<br><br>$C_{64}H_{70}N_{14}O_{13}$<br>MA 64<br>MW 1242.52	Peptide: HWYQNWYF<br><br>$C_{64}H_{70}N_{14}O_{13}$<br>MA 63<br>MW 1242.52	Peptide: HWYQNWYA<br><br>$C_{58}H_{66}N_{14}O_{13}$<br>MA 58<br>MW 1166.49
Peptide: YWHQNWYW<br><br>$C_{66}H_{71}N_{15}O_{13}$<br>MA 53<br>MW 1281.54	Peptide: QNWYF<br><br>$C_{38}H_{44}N_8O_9$<br>MA 50<br>MW 756.32	Peptide: QNYWF<br><br>$C_{38}H_{44}N_8O_9$<br>MA 50<br>MW 756.32

Peptide: QNWYA<br><br>$C_{32}H_{40}N_8O_9$<br>MA 45<br>MW 680.29	Peptide: NWYF<br><br>$C_{33}H_{36}N_6O_7$<br>MA 42<br>MW 628.26	Peptide: NYWF<br><br>$C_{33}H_{36}N_6O_7$<br>MA 42<br>MW 628.26
Peptide: ASGNQSGV<br><br>$C_{27}H_{46}N_{10}O_{13}$<br>MA 40<br>MW 718.32	Peptide: QNWYW<br><br>$C_{40}H_{45}N_9O_9$<br>MA 40<br>MW 795.33	Peptide: FGSNQ<br><br>$C_{23}H_{33}N_7O_9$<br>MA 37<br>MW 551.23
Peptide: NWYA<br><br>$C_{27}H_{32}N_6O_7$<br>MA 37<br>MW 552.23	Peptide: WYF<br><br>$C_{29}H_{30}N_4O_5$<br>MA 34<br>MW 514.22	Peptide: YWF<br><br>$C_{29}H_{30}N_4O_5$<br>MA 34<br>MW 514.22
Peptide: NWYW<br><br>$C_{35}H_{37}N_7O_7$<br>MA 32<br>MW 667.28	Peptide: FGSN<br><br>$C_{18}H_{25}N_5O_7$<br>MA 29<br>MW 423.18	Peptide: WYA<br><br>$C_{23}H_{26}N_4O_5$<br>MA 29<br>MW 438.19
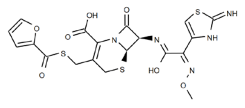<br>PubChem CID: 5484735<br><br>$C_{19}H_{17}N_5O_7S_3$<br>MA 27<br>MW 523.03	Peptide: GSGNQ<br><br>$C_{16}H_{27}N_7O_9$<br>MA 27<br>MW 461.19	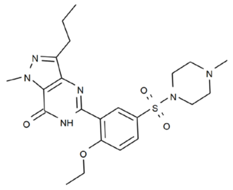<br>PubChem CID: 135398744<br><br>$C_{22}H_{30}N_6O_4S$<br>MA 26<br>MW 474.2

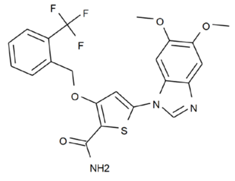<br>PubChem CID: 9826308<br><br>$C_{22}H_{18}F_3N_3O_4S$<br>MA 25<br>MW 477.1	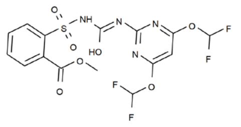<br>PubChem CID: 101525<br><br>$C_{15}H_{12}F_4N_4O_7S$<br>MA 24<br>MW 468.04	Peptide: WYW<br><br>$C_{31}H_{31}N_5O_5$<br>MA 24<br>MW 553.23
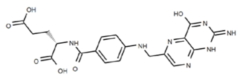<br>PubChem CID: 135398658<br><br>$C_{19}H_{19}N_7O_6$<br>MA 23<br>MW 441.14	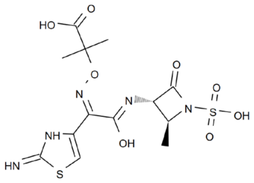<br>PubChem CID: 5459211<br><br>$C_{13}H_{17}N_5O_8S_2$<br>MA 23<br>MW 435.05	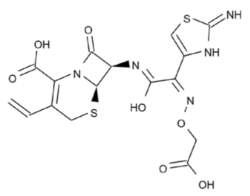<br>PubChem CID: 6321411<br><br>$C_{16}H_{15}N_5O_7S_2$<br>MA 23<br>MW 453.04
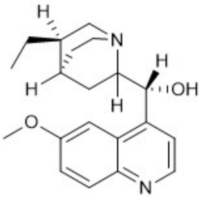<br>PubChem CID: 16212138<br><br>$C_{27}H_{29}ClN_2O_3$<br>MA 23<br>MW 464.19	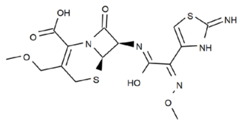<br>PubChem CID: 6321413<br><br>$C_{15}H_{17}N_5O_6S_2$<br>MA 23<br>MW 427.06	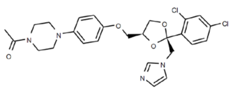<br>PubChem CID: 456201<br><br>$C_{26}H_{28}Cl_2N_4O_4$<br>MA 23<br>MW 530.15

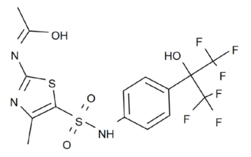<br>PubChem CID: 44241473<br><br>$C_{15}H_{13}F_6N_3O_4S_2$<br>MA 23<br>MW 477.03	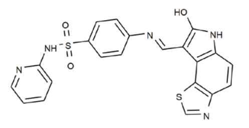<br>PubChem CID: 3536<br><br>$C_{21}H_{15}N_5O_3S_2$<br>MA 23<br>MW 449.06	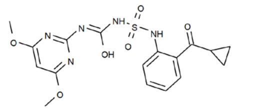<br>PubChem CID: 6451137<br><br>$C_{17}H_{19}N_5O_6S$<br>MA 22<br>MW 421.11
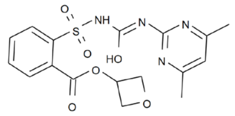<br>PubChem CID: 86443<br><br>$C_{17}H_{18}N_4O_6S$<br>MA 22<br>MW 406.09	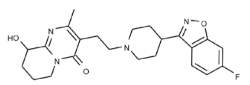<br>PubChem CID: 115237<br><br>$C_{23}H_{27}FN_4O_3$<br>MA 22<br>MW 426.21	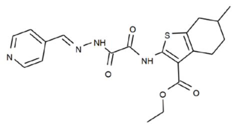<br>PubChem CID: 5893995<br><br>$C_{20}H_{22}N_4O_4S$<br>MA 22<br>MW 414.14
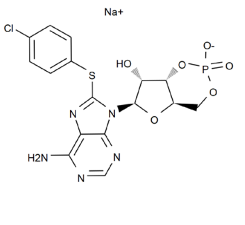<br>PubChem CID: 23672705<br><br>$C_{16}H_{14}ClN_5NaO_6PS$<br>MA 22<br>MW 493.0	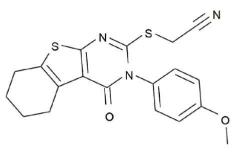<br>PubChem CID: 1913406<br><br>$C_{19}H_{17}N_3O_2S_2$<br>MA 21<br>MW 383.08	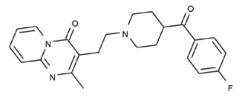<br>PubChem CID: 4847<br><br>$C_{23}H_{24}FN_3O_2$<br>MA 21<br>MW 393.19

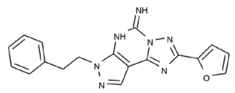<br>PubChem CID: 176408<br><br>$C_{18}H_{15}N_7O$<br>MA 20<br>MW 345.13	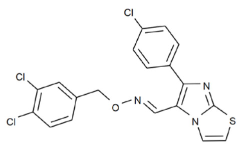<br>PubChem CID: 5912745<br><br>$C_{19}H_{12}Cl_3N_3OS$<br>MA 20<br>MW 434.98	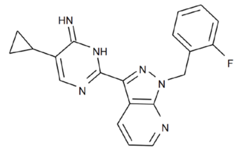<br>PubChem CID: 9798973<br><br>$C_{20}H_{17}FN_6$<br>MA 20<br>MW 360.15
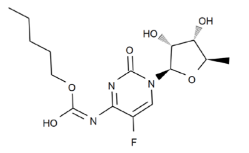<br>PubChem CID: 60953<br><br>$C_{15}H_{22}FN_3O_6$<br>MA 19<br>MW 359.15	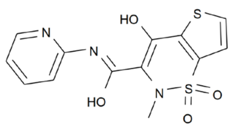<br>PubChem CID: 54677971<br><br>$C_{13}H_{11}N_3O_4S_2$<br>MA 19<br>MW 337.02	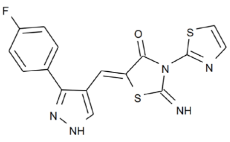<br>PubChem CID: 1239801<br><br>$C_{16}H_{10}FN_5OS_2$<br>MA 19<br>MW 371.03
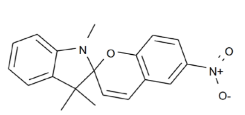<br>PubChem CID: 99766<br><br>$C_{19}H_{18}N_2O_3$<br>MA 18<br>MW 322.13	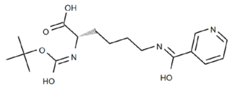<br>PubChem CID: 7018782<br><br>$C_{17}H_{25}N_3O_5$<br>MA 18<br>MW 351.18	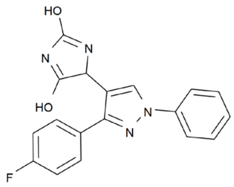<br>PubChem CID: 660311<br><br>$C_{18}H_{13}FN_4O_2$<br>MA 18<br>MW 336.1

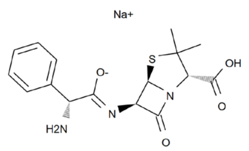<br>PubChem CID: 101603128<br><br>$C_{16}H_{18}N_3NaO_4S$<br>MA 18<br>MW 371.09	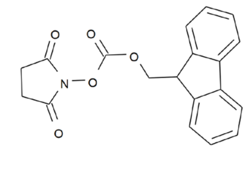<br>PubChem CID: 134122<br><br>$C_{19}H_{15}NO_5$<br>MA 18<br>MW 337.1	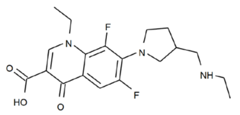<br>PubChem CID: 121833<br><br>$C_{19}H_{23}F_2N_3O_3$<br>MA 18<br>MW 379.17
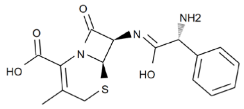<br>PubChem CID: 27447<br><br>$C_{16}H_{17}N_3O_4S$<br>MA 18<br>MW 347.09	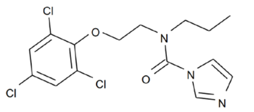<br>PubChem CID: 73665<br><br>$C_{15}H_{16}Cl_3N_3O_2$<br>MA 18<br>MW 375.03	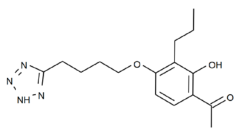<br>PubChem CID: 3969<br><br>$C_{16}H_{22}N_4O_3$<br>MA 17<br>MW 318.17
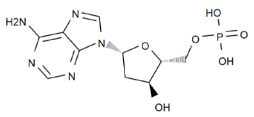<br>PubChem CID: 12599<br><br>$C_{10}H_{14}N_5O_6P$<br>MA 17<br>MW 331.07	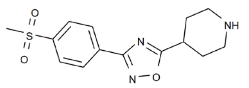<br>PubChem CID: 2761149<br><br>$C_{14}H_{17}N_3O_3S$<br>MA 17<br>MW 307.1	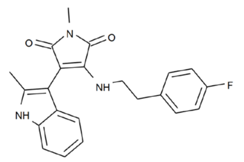<br>PubChem CID: 25209788<br><br>$C_{22}H_{20}FN_3O_2$<br>MA 17<br>MW 377.15

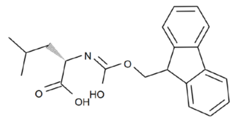<br>PubChem CID: 1549133<br><br>$C_{21}H_{23}NO_4$<br>MA 17<br>MW 353.16	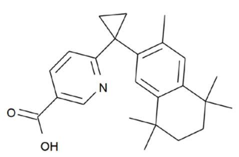<br>PubChem CID: 3922<br><br>$C_{24}H_{29}NO_2$<br>MA 17<br>MW 363.22	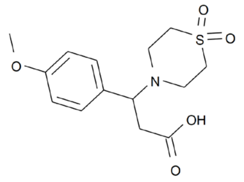<br>PubChem CID: 2766026<br><br>$C_{14}H_{19}NO_5S$<br>MA 16<br>MW 313.1
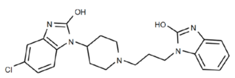<br>PubChem CID: 3151<br><br>$C_{22}H_{24}ClN_5O_2$<br>MA 16<br>MW 425.16	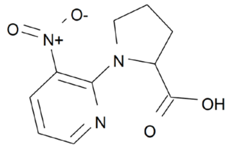<br>PubChem CID: 2766183<br><br>$C_{10}H_{11}N_3O_4$<br>MA 16<br>MW 237.07	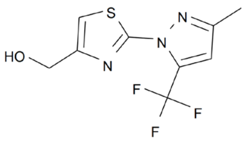<br>PubChem CID: 2763602<br><br>$C_9H_8F_3N_3OS$<br>MA 16<br>MW 263.03
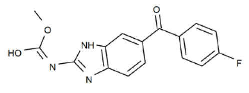<br>PubChem CID: 35802<br><br>$C_{16}H_{12}FN_3O_3$<br>MA 16<br>MW 313.09	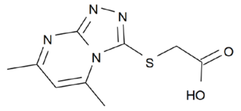<br>PubChem CID: 705437<br><br>$C_9H_{10}N_4O_2S$<br>MA 16<br>MW 238.05	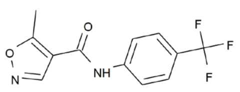<br>PubChem CID: 3899<br><br>$C_{12}H_9F_3N_2O_2$<br>MA 16<br>MW 270.06

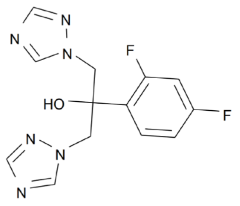<br>PubChem CID: 3365<br><br>$C_{13}H_{12}F_2N_6O$<br>MA 15<br>MW 306.1	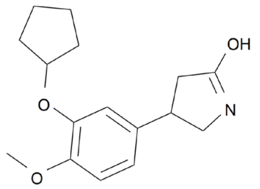<br>PubChem CID: 5092<br><br>$C_{16}H_{21}NO_3$<br>MA 15<br>MW 275.15	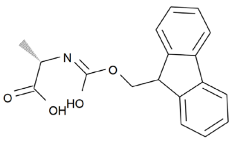<br>PubChem CID: 6364642<br><br>$C_{18}H_{17}NO_4$<br>MA 15<br>MW 311.12
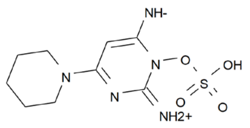<br>PubChem CID: 4219798<br><br>$C_9H_{15}N_5O_4S$<br>MA 15<br>MW 289.08	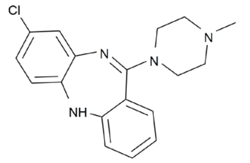<br>PubChem CID: 135398737<br><br>$C_{18}H_{19}ClN_4$<br>MA 15<br>MW 326.13	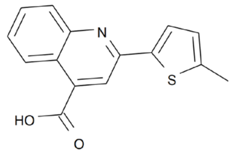<br>PubChem CID: 673714<br><br>$C_{15}H_{11}NO_2S$<br>MA 14<br>MW 269.05
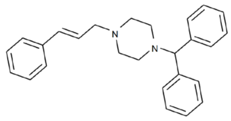<br>PubChem CID: 1547484<br><br>$C_{26}H_{28}N_2$<br>MA 14<br>MW 368.23	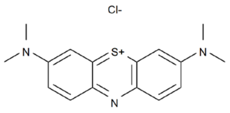<br>PubChem CID: 6099<br><br>$C_{16}H_{18}ClN_3S$<br>MA 14<br>MW 319.09	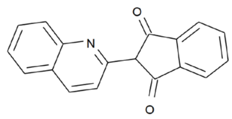<br>PubChem CID: 6731<br><br>$C_{18}H_{11}NO_2$<br>MA 14<br>MW 273.08

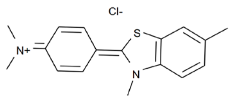<br>PubChem CID: 16953<br><br>$C_{17}H_{19}ClN_2S$<br>MA 14<br>MW 318.1	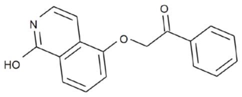<br>PubChem CID: 25015515<br><br>$C_{17}H_{13}NO_3$<br>MA 14<br>MW 279.09	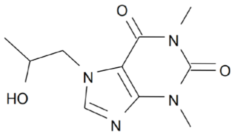<br>PubChem CID: 4977<br><br>$C_{10}H_{14}N_4O_3$<br>MA 14<br>MW 238.11
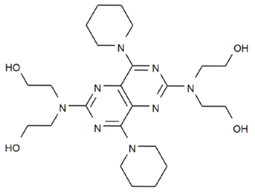<br>PubChem CID: 3108<br><br>$C_{24}H_{40}N_8O_4$<br>MA 14<br>MW 504.32	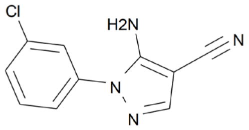<br>PubChem CID: 734496<br><br>$C_{10}H_7ClN_4$<br>MA 13<br>MW 218.04	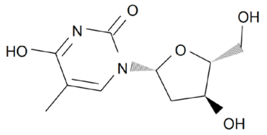<br>PubChem CID: 5789<br><br>$C_{10}H_{14}N_2O_5$<br>MA 13<br>MW 242.09
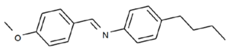<br>PubChem CID: 33363<br><br>$C_{18}H_{21}NO$<br>MA 13<br>MW 267.16	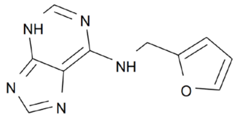<br>PubChem CID: 3830<br><br>$C_{10}H_9N_5O$<br>MA 13<br>MW 215.08	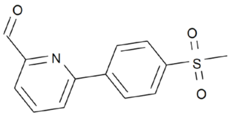<br>PubChem CID: 16217973<br><br>$C_{13}H_{11}NO_3S$<br>MA 13<br>MW 261.05

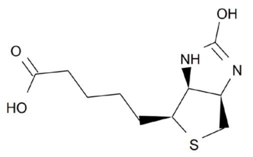<br>PubChem CID: 171548<br><br>$C_{10}H_{16}N_2O_3S$<br>MA 13<br>MW 244.09	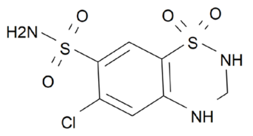<br>PubChem CID: 3639<br><br>$C_7H_8ClN_3O_4S_2$<br>MA 13<br>MW 296.96	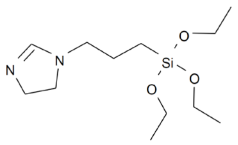<br>PubChem CID: 93933<br><br>$C_{12}H_{26}N_2O_3Si$<br>MA 13<br>MW 274.17
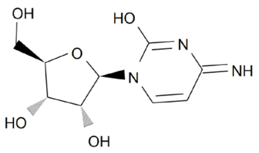<br>PubChem CID: 6175<br><br>$C_9H_{13}N_3O_5$<br>MA 13<br>MW 243.09	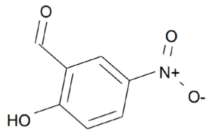<br>PubChem CID: 66808<br><br>$C_7H_5NO_4$<br>MA 12<br>MW 167.02	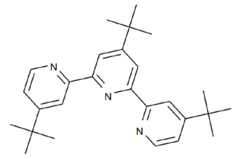<br>PubChem CID: 4229824<br><br>$C_{27}H_{35}N_3$<br>MA 12<br>MW 401.28
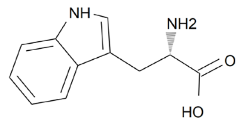<br>PubChem CID: 6305<br><br>$C_{11}H_{12}N_2O_2$<br>MA 12<br>MW 204.09	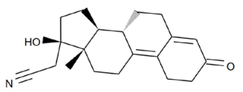<br>PubChem CID: 68861<br><br>$C_{20}H_{25}NO_2$<br>MA 12<br>MW 311.19	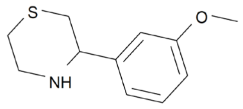<br>PubChem CID: 45036859<br><br>$C_{11}H_{15}NOS$<br>MA 11<br>MW 209.09

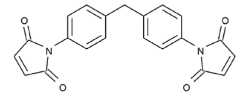<br>PubChem CID: 83648<br><br>$C_{21}H_{14}N_2O_4$<br>MA 11<br>MW 358.1	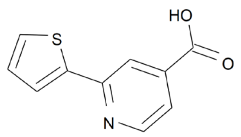<br>PubChem CID: 4739102<br><br>$C_{10}H_7NO_2S$<br>MA 11<br>MW 205.02	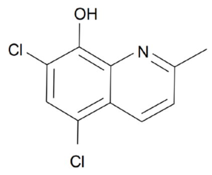<br>PubChem CID: 6301<br><br>$C_{10}H_7Cl_2NO$<br>MA 11<br>MW 226.99
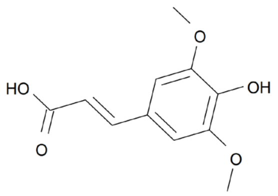<br>PubChem CID: 637775<br><br>$C_{11}H_{12}O_5$<br>MA 11<br>MW 224.07	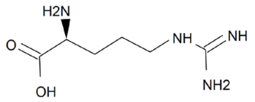<br>PubChem CID: 6322<br><br>$C_6H_{14}N_4O_2$<br>MA 10<br>MW 174.11	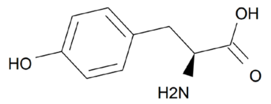<br>PubChem CID: 6057<br><br>$C_9H_{11}NO_3$<br>MA 10<br>MW 181.07
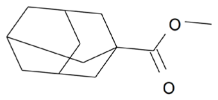<br>PubChem CID: 136553<br><br>$C_{12}H_{18}O_2$<br>MA 9<br>MW 194.13	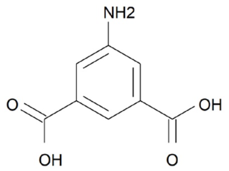<br>PubChem CID: 66833<br><br>$C_8H_7NO_4$<br>MA 9<br>MW 181.04	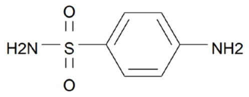<br>PubChem CID: 5333<br><br>$C_6H_8N_2O_2S$<br>MA 9<br>MW 172.03

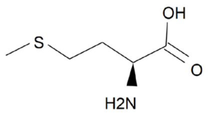<br>PubChem CID: 6137<br><br>$C_5H_{11}NO_2S$<br>MA 8<br>MW 149.05	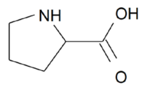<br>PubChem CID: 614<br><br>$C_5H_9NO_2$<br>MA 8<br>MW 115.06	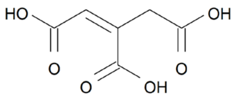<br>PubChem CID: 643757<br><br>$C_6H_6O_6$<br>MA 7<br>MW 174.02
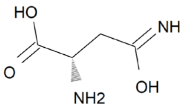<br>PubChem CID: 6267<br><br>$C_4H_8N_2O_3$<br>MA 7<br>MW 132.05	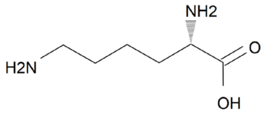<br>PubChem CID: 5962<br><br>$C_6H_{14}N_2O_2$<br>MA 7<br>MW 146.11	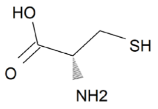<br>PubChem CID: 5862<br><br>$C_3H_7NO_2S$<br>MA 6<br>MW 121.02
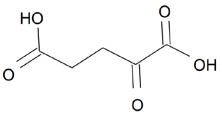<br>PubChem CID: 51<br><br>$C_5H_6O_5$<br>MA 6<br>MW 146.02	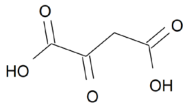<br>PubChem CID: 970<br><br>$C_4H_4O_5$<br>MA 6<br>MW 132.01	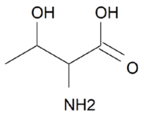<br>PubChem CID: 205<br><br>$C_4H_9NO_3$<br>MA 6<br>MW 119.06

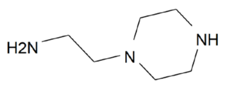	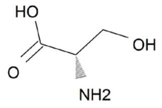	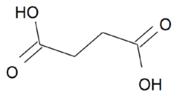
PubChem CID: 8795<br><br>$C_6H_{15}N_3$<br>MA 5<br>MW 129.13	PubChem CID: 5951<br><br>$C_3H_7NO_3$<br>MA 5<br>MW 105.04	PubChem CID: 1110<br><br>$C_4H_6O_4$<br>MA 5<br>MW 118.03

Supplementary Table 1. Compounds Analyzed via Mass Spec, sorted by MA.

The following table contains details for molecules included in figure 2B in the main text.

**Name**	**InChI**	**MA**	**MW**	**Category**
Ceftiofur	InChI=1S/C19H17N5O7S3/c1-30-23-11(9-7-34-19(20)21-9)14(25)22-12-15(26)24-13(17(27)28)8(5-32-16(12)24)6-33-18(29)10-3-2-4-31-10/h2-4,7,12,16H,5-6H2,1H3,(H2,20,21)(H,22,25)(H,27,28)/b23-11-/t12-,16-/m1/s1	27	523.029	Pharmaceutical
Sildenafil	InChI=1S/C22H30N6O4S/c1-5-7-17-19-20(27(4)25-17)22(29)24-21(23-19)16-14-15(8-9-18(16)32-6-2)33(30,31)28-12-10-26(3)11-13-28/h8-9,14H,5-7,10-13H2,1-4H3,(H,23,24,29)	26	474.205	Pharmaceutical
5-(5,6-dimethoxy-1H-benzimidazol-1-yl)-3-((2-(trifluoromethyl)benzyl)oxy)thiophene-2-carboxamide	InChI=1S/C22H18F3N3O4S/c1-30-16-7-14-15(8-17(16)31-2)28(11-27-14)19-9-18(20(33-19)21(26)29)32-10-12-5-3-4-6-13(12)22(23,24)25/h3-9,11H,10H2,1-2H3,(H2,26,29)	25	477.097	Industrial Compound
Primisulfuron-methyl	InChI=1S/C15H12F4N4O7S/c1-28-11(24)7-4-2-3-5-8(7)31(26,27)23-15(25)22-14-20-9(29-12(16)17)6-10(21-14)30-13(18)19/h2-6,12-13H,1H3,(H2,20,21,22,23,25)	24	468.036	Industrial Compound

Folic Acid	InChI=1S/C19H19N7O6/c20-19-25-15-14(17(30)26-19)23-11(8-22-15)7-21-10-3-1-9(2-4-10)16(29)24-12(18(31)32)5-6-13(27)28/h1-4,8,12,21H,5-7H2,(H,24,29)(H,27,28)(H,31,32)(H3,20,22,25,26,30)/t12-/m0/s1	23	441.140	Natural Product
Azetreonam	InChI=1S/C13H17N5O8S2/c1-5-7(10(20)18(5)28(23,24)25)16-9(19)8(6-4-27-12(14)15-6)17-26-13(2,3)11(21)22/h4-5,7H,1-3H3,(H2,14,15)(H,16,19)(H,21,22)(H,23,24,25)/b17-8-/t5-,7-/m0/s1	23	435.052	Pharmaceutical
(3?,9S)-6'-Methoxy-10,11-dihydrocinchonan-9-yl 4-chlorobenzoate	InChI=1S/C27H29ClN2O3/c1-3-17-16-30-13-11-19(17)14-25(30)26(33-27(31)18-4-6-20(28)7-5-18)22-10-12-29-24-9-8-21(32-2)15-23(22)24/h4-10,12,15,17,19,25-26H,3,11,13-14,16H2,1-2H3/t17-,19+,25-,26+/m1/s1	23	464.187	Industrial Compound
Cefpodoxime	InChI=1S/C15H17N5O6S2/c1-25-3-6-4-27-13-9(12(22)20(13)10(6)14(23)24)18-11(21)8(19-26-2)7-5-28-15(16)17-7/h5,9,13H,3-4H2,1-2H3,(H2,16,17)(H,18,21)(H,23,24)/b19-8-/t9-,13-/m1/s1	23	427.062	Pharmaceutical
Ketoconazole	InChI=1S/C26H28Cl2N4O4/c1-19(33)31-10-12-32(13-11-31)21-3-5-22(6-4-21)34-15-23-16-35-26(36-23,17-30-9-8-29-18-30)24-7-2-20(27)14-25(24)28/h2-9,14,18,23H,10-13,15-17H2,1H3/t23-,26-/m0/s1	23	530.149	Pharmaceutical
SR1001	InChI=1S/C15H13F6N3O4S2/c1-7-11(29-12(22-7)23-8(2)25)30(27,28)24-10-5-3-9(4-6-10)13(26,14(16,17)18)15(19,20)21/h3-6,24,26H,1-2H3,(H,22,23,25)	23	477.025	Pharmaceutical
Cefixime CDS 021590	InChI=1S/C16H15N5O7S2/c1-2-6-4-29-14-10(13(25)21(14)11(6)15(26)27)19-12(24)9(20-28-3-8(22)23)7-5-30-16(17)18-7/h2,5,10,14H,1,3-4H2,(H2,17,18)(H,19,24)(H,22,23)(H,26,27)/b20-9-/t10-,14-/m1/s1	23	453.041	Pharmaceutical

4-{[(E)-(7-Oxo-6,7-dihydro-8H-[1,3]thiazolo[5,4-e]indol-8-ylidene)methyl]amino}-N-(2-pyridinyl)benzenesulfonamide	InChI=1S/C21H15N5O3S2/c27-21-15(19-16(25-21)8-9-17-20(19)30-12-24-17)11-23-13-4-6-14(7-5-13)31(28,29)26-18-3-1-2-10-22-18/h1-12,25,27H,(H,22,26)	23	449.062	Industrial Compound
Oxasulfuron	InChI=1S/C17H18N4O6S/c1-10-7-11(2)19-16(18-10)20-17(23)21-28(24,25)14-6-4-3-5-13(14)15(22)27-12-8-26-9-12/h3-7,12H,8-9H2,1-2H3,(H2,18,19,20,21,23)	22	406.095	Industrial Compound
Paliperidone	InChI=1S/C23H27FN4O3/c1-14-17(23(30)28-9-2-3-19(29)22(28)25-14)8-12-27-10-6-15(7-11-27)21-18-5-4-16(24)13-20(18)31-26-21/h4-5,13,15,19,29H,2-3,6-12H2,1H3	22	426.207	Pharmaceutical
Cyclosulfamuron	InChI=1S/C17H19N5O6S/c1-27-13-9-14(28-2)19-16(18-13)20-17(24)22-29(25,26)21-12-6-4-3-5-11(12)15(23)10-7-8-10/h3-6,9-10,21H,7-8H2,1-2H3,(H2,18,19,20,22,24)	22	421.106	Industrial Compound
8-(4-Chlorophenylthio)adenosine 3?,5?-cyclic monophosphate sodium salt	InChI=1S/C16H15ClN5O6PS.Na/c17-7-1-3-8(4-2-7)30-16-21-10-13(18)19-6-20-14(10)22(16)15-11(23)12-9(27-15)5-26-29(24,25)28-12;/h1-4,6,9,11-12,15,23H,5H2,(H,24,25)(H2,18,19,20);/q;+1/p-1/t9-,11-,12-,15-;/m1./s1	22	492.999	Industrial Compound
Necrostatin-5	InChI=1S/C19H17N3O2S2/c1-24-13-8-6-12(7-9-13)22-18(23)16-14-4-2-3-5-15(14)26-17(16)21-19(22)25-11-10-20/h6-9H,2-5,11H2,1H3	21	383.076	Pharmaceutical
Pirenperone	InChI=1S/C23H24FN3O2/c1-16-20(23(29)27-12-3-2-4-21(27)25-16)11-15-26-13-9-18(10-14-26)22(28)17-5-7-19(24)8-6-17/h2-8,12,18H,9-11,13-15H2,1H3	21	393.185	Pharmaceutical
Bay	InChI=1S/C20H17FN6/c21-16-6-2-1-4-13(16)11-27-20-14(5-3-9-23-20)17(26-27)19-24-10-15(12-7-8-12)18(22)25-19/h1-6,9-10,12H,7-8,11H2,(H2,22,24,25)	20	360.150	Industrial Compound

SCH-58261 (7-(2-phenylethyl)-5-amino-2-(2-furyl)-pyrazolo-[4,3-e]-1,2,4-triazolo[1,5-c]pyrimidine)	InChI=1S/C18H15N7O/c19-18-22-16-13(11-20-24(16)9-8-12-5-2-1-3-6-12)17-21-15(23-25(17)18)14-7-4-10-26-14/h1-7,10-11H,8-9H2,(H2,19,22)	20	345.134	Pharmaceutical
CITCO	InChI=1S/C19H12Cl3N3OS/c20-14-4-2-13(3-5-14)18-17(25-7-8-27-19(25)24-18)10-23-26-11-12-1-6-15(21)16(22)9-12/h1-10H,11H2/b23-10+	20	434.977	Natural Product
Necrostatin-7	InChI=1S/C16H10FN5OS2/c17-11-3-1-9(2-4-11)13-10(8-20-21-13)7-12-14(23)22(15(18)25-12)16-19-5-6-24-16/h1-8,18H,(H,20,21)/b12-7-,18-15?	19	371.031	Pharmaceutical
Tenoxican	InChI=1S/C13H11N3O4S2/c1-16-10(13(18)15-9-4-2-3-6-14-9)11(17)12-8(5-7-21-12)22(16,19)20/h2-7,17H,1H3,(H,14,15,18)	19	337.019	Pharmaceutical
Capecitabine	InChI=1S/C15H22FN3O6/c1-3-4-5-6-24-15(23)18-12-9(16)7-19(14(22)17-12)13-11(21)10(20)8(2)25-13/h7-8,10-11,13,20-21H,3-6H2,1-2H3,(H,17,18,22,23)/t8-,10-,11-,13-/m1/s1	19	359.149	Pharmaceutical
Prochloraz	InChI=1S/C15H16Cl3N3O2/c1-2-4-20(15(22)21-5-3-19-10-21)6-7-23-14-12(17)8-11(16)9-13(14)18/h3,5,8-10H,2,4,6-7H2,1H3	18	375.031	Pharmaceutical
(2S)-2-[(tert-Butoxycarbonyl)amino]-6-[(3-pyridinylcarbonyl)amino]hexanoic acid	InChI=1S/C17H25N3O5/c1-17(2,3)25-16(24)20-13(15(22)23)8-4-5-10-19-14(21)12-7-6-9-18-11-12/h6-7,9,11,13H,4-5,8,10H2,1-3H3,(H,19,21)(H,20,24)(H,22,23)/t13-/m0/s1	18	351.179	Pharmaceutical
1-ethyl-7-{3-[(ethylamino)methyl]-1-pyrrolidinyl}-6,8-difluoro-4-oxo-1,4-dihydro-3-quinolinecarboxylic acid	InChI=1S/C19H23F2N3O3/c1-3-22-8-11-5-6-24(9-11)17-14(20)7-12-16(15(17)21)23(4-2)10-13(18(12)25)19(26)27/h7,10-11,22H,3-6,8-9H2,1-2H3,(H,26,27)	18	379.171	Pharmaceutical
1,3,3-Trimethylindolino-6'-nitrobenzo-pyrylospiran	InChI=1S/C19H18N2O3/c1-18(2)15-6-4-5-7-16(15)20(3)19(18)11-10-13-12-14(21(22)23)8-9-17(13)24-19/h4-12H,1-3H3	18	322.132	Industrial Compound

5-[3-(4-Fluorophenyl)-1-phenyl-1H-pyrazol-4-yl]-2,4-imidazolidinedione(5-(1,3-diaryl-1H-pyrazol-4-yl)hydantoin, 5-[3-(4-fluorophenyl)-1-phenyl-1H-pyrazol-4-yl]-2,4-imidazolidinedione)	InChI=1S/C18H13FN4O2/c19-12-8-6-11(7-9-12)15-14(16-17(24)21-18(25)20-16)10-23(22-15)13-4-2-1-3-5-13/h1-10,16H,(H2,20,21,24,25)	18	336.102	Industrial Compound
Ampilicilin Na Salt	InChI=1S/C16H19N3O4S.Na/c1-16(2)11(15(22)23)19-13(21)10(14(19)24-16)18-12(20)9(17)8-6-4-3-5-7-8;/h3-7,9-11,14H,17H2,1-2H3,(H,18,20)(H,22,23);/q;+1/p-1/t9-,10-,11+,14-;/m1./s1	18	371.092	Pharmaceutical
Fmoc Nhydroxysuccinimide ester	InChI=1S/C19H15NO5/c21-17-9-10-18(22)20(17)25-19(23)24-11-16-14-7-3-1-5-12(14)13-6-2-4-8-15(13)16/h1-8,16H,9-11H2	18	337.095	Industrial Compound
Cefalexin	InChI=1S/C16H17N3O4S/c1-8-7-24-15-11(14(21)19(15)12(8)16(22)23)18-13(20)10(17)9-5-3-2-4-6-9/h2-6,10-11,15H,7,17H2,1H3,(H,18,20)(H,22,23)/t10-,11-,15-/m1/s1	18	347.094	Pharmaceutical
LY-171,883	InChI=1S/C16H22N4O3/c1-3-6-13-14(9-8-12(11(2)21)16(13)22)23-10-5-4-7-15-17-19-20-18-15/h8-9,22H,3-7,10H2,1-2H3,(H,17,18,19,20)	17	318.169	Industrial Compound
FMOC Leucine	InChI=1S/C21H23NO4/c1-13(2)11-19(20(23)24)22-21(25)26-12-18-16-9-5-3-7-14(16)15-8-4-6-10-17(15)18/h3-10,13,18-19H,11-12H2,1-2H3,(H,22,25)(H,23,24)/t19-/m0/s1	17	353.163	Industrial Compound
2?-Deoxyadenosine 5?-monophosphate	InChI=1S/C10H14N5O6P/c11-9-8-10(13-3-12-9)15(4-14-8)7-1-5(16)6(21-7)2-20-22(17,18)19/h3-7,16H,1-2H2,(H2,11,12,13)(H2,17,18,19)/t5-,6+,7+/m0/s1	17	331.068	Metabolite
CDS014803	InChI=1S/C14H17N3O3S/c1-21(18,19)12-4-2-10(3-5-12)13-16-14(20-17-13)11-6-8-15-9-7-11/h2-5,11,15H,6-9H2,1H3	17	307.099	Industrial Compound

IM12	InChI=1S/C22H20FN3O2/c1-13-18(16-5-3-4-6-17(16)25-13)19-20(22(28)26(2)21(19)27)24-12-11-14-7-9-15(23)10-8-14/h3-10,24-25H,11-12H2,1-2H3	17	377.154	Industrial Compound
(6-[1-(3,5,5,8,8-Pentamethyl-5,6,7,8-tetrahydro-2-naphthalenyl)cyclopropyl]nicotinic acid)	InChI=1S/C24H29NO2/c1-15-12-18-19(23(4,5)9-8-22(18,2)3)13-17(15)24(10-11-24)20-7-6-16(14-25-20)21(26)27/h6-7,12-14H,8-11H2,1-5H3,(H,26,27)	17	363.220	Industrial Compound
Domperidone	InChI=1S/C22H24ClN5O2/c23-15-6-7-20-18(14-15)25-22(30)28(20)16-8-12-26(13-9-16)10-3-11-27-19-5-2-1-4-17(19)24-21(27)29/h1-2,4-7,14,16H,3,8-13H2,(H,24,29)(H,25,30)	16	425.162	Pharmaceutical
1,3-Nitropyridine	InChI=1S/C10H11N3O4/c14-10(15)8-4-2-6-12(8)9-7(13(16)17)3-1-5-11-9/h1,3,5,8H,2,4,6H2,(H,14,15)	16	237.075	Industrial Compound
3-(1,1-Dioxido-4-thiomorpholinyl)-3-(4-methoxyphenyl)propanoic acid	InChI=1S/C14H19NO5S/c1-20-12-4-2-11(3-5-12)13(10-14(16)17)15-6-8-21(18,19)9-7-15/h2-5,13H,6-10H2,1H3,(H,16,17)	16	313.098	Metabolite
CDS016302	InChI=1S/C9H8F3N3OS/c1-5-2-7(9(10,11)12)15(14-5)8-13-6(3-16)4-17-8/h2,4,16H,3H2,1H3	16	263.034	Industrial Compound
Flubendazole	InChI=1S/C16H12FN3O3/c1-23-16(22)20-15-18-12-7-4-10(8-13(12)19-15)14(21)9-2-5-11(17)6-3-9/h2-8H,1H3,(H2,18,19,20,22)	16	313.086	Pharmaceutical
Pyrimidin Acetic Acid	InChI=1S/C9H10N4O2S/c1-5-3-6(2)13-8(10-5)11-12-9(13)16-4-7(14)15/h3H,4H2,1-2H3,(H,14,15)	16	238.052	Metabolite
leflunomide	InChI=1S/C12H9F3N2O2/c1-7-10(6-16-19-7)11(18)17-9-4-2-8(3-5-9)12(13,14)15/h2-6H,1H3,(H,17,18)	16	270.062	Pharmaceutical
Minoxidil sulfate salt	InChI=1S/C9H15N5O4S/c10-7-6-8(13-4-2-1-3-5-13)12-9(11)14(7)18-19(15,16)17/h6H,1-5H2,(H4,10,11,12,15,16,17)/p+1	15	289.084	Pharmaceutical
Rolipram	InChI=1S/C16H21NO3/c1-19-14-7-6-11(12-9-16(18)17-10-12)8-15(14)20-13-4-2-3-5-13/h6-8,12-13H,2-5,9-10H2,1H3,(H,17,18)	15	275.152	Pharmaceutical
Fluconazole	InChI=1S/C13H12F2N6O/c14-10-1-2-11(12(15)3-10)13(22,4-20-8-16-6-18-20)5-21-9-17-7-19-21/h1-3,6-9,22H,4-5H2	15	306.104	Pharmaceutical

Fmoc L-Alanine	InChI=1S/C18H17NO4/c1-11(17(20)21)19-18(22)23-10-16-14-8-4-2-6-12(14)13-7-3-5-9-15(13)16/h2-9,11,16H,10H2,1H3,(H,19,22)(H,20,21)/t11-/m0/s1	15	311.116	Industrial Compound
Clozapine	InChI=1S/C18H19ClN4/c1-22-8-10-23(11-9-22)18-14-4-2-3-5-15(14)20-16-7-6-13(19)12-17(16)21-18/h2-7,12,20H,8-11H2,1H3	15	326.130	Pharmaceutical
2-(5-Methyl-2-thienyl)-4-quinolinecarboxylic acid	InChI=1S/C15H11NO2S/c1-9-6-7-14(19-9)13-8-11(15(17)18)10-4-2-3-5-12(10)16-13/h2-8H,1H3,(H,17,18)	14	269.051	Industrial Compound
Cinnarizine	InChI=1S/C26H28N2/c1-4-11-23(12-5-1)13-10-18-27-19-21-28(22-20-27)26(24-14-6-2-7-15-24)25-16-8-3-9-17-25/h1-17,26H,18-22H2/b13-10+	14	368.225	Pharmaceutical
Methylene Blue	InChI=1S/C16H18N3S.ClH/c1-18(2)11-5-7-13-15(9-11)20-16-10-12(19(3)4)6-8-14(16)17-13;/h5-10H,1-4H3;1H/q+1;/p-1	14	319.091	Pharmaceutical
Quinoline Yellow	InChI=1S/C18H11NO2/c20-17-12-6-2-3-7-13(12)18(21)16(17)15-10-9-11-5-1-4-8-14(11)19-15/h1-10,16H	14	273.079	Industrial Compound
Thioflavin T	InChI=1S/C17H19N2S.ClH/c1-12-5-10-15-16(11-12)20-17(19(15)4)13-6-8-14(9-7-13)18(2)3;/h5-11H,1-4H3;1H/q+1;/p-1	14	318.096	Industrial Compound
5-(2-Oxo-2-phenylethoxy)-1(2H)-isoquinolinone	InChI=1S/C17H13NO3/c19-15(12-5-2-1-3-6-12)11-21-16-8-4-7-14-13(16)9-10-18-17(14)20/h1-10H,11H2,(H,18,20)	14	279.090	Industrial Compound
7-(2-HYDROXYPROPYL)THEOPHYLLINE	InChI=1S/C10H14N4O3/c1-6(15)4-14-5-11-8-7(14)9(16)13(3)10(17)12(8)2/h5-6,15H,4H2,1-3H3	14	238.107	Pharmaceutical
Dipyridamole	InChI=1S/C24H40N8O4/c33-15-11-31(12-16-34)23-26-20-19(21(27-23)29-7-3-1-4-8-29)25-24(32(13-17-35)14-18-36)28-22(20)30-9-5-2-6-10-30/h33-36H,1-18H2	14	504.317	Pharmaceutical
Hydrochlorothiazide	InChI=1S/C7H8ClN3O4S2/c8-4-1-5-7(2-6(4)16(9,12)13)17(14,15)11-3-10-5/h1-2,10-11H,3H2,(H2,9,12,13)	13	296.964	Pharmaceutical

5-Amino-1-(3-chlorophenyl)-1H-pyrazole-4-carbonitrile	InChI=1S/C10H7ClN4/c11-8-2-1-3-9(4-8)15-10(13)7(5-12)6-14-15/h1-4,6H,13H2	13	218.036	Pharmaceutical
Thymidine	InChI=1S/C10H14N2O5/c1-5-3-12(10(16)11-9(5)15)8-2-6(14)7(4-13)17-8/h3,6-8,13-14H,2,4H2,1H3,(H,11,15,16)/t6-,7+,8+/m0/s1	13	242.090	Metabolite
N-(4-Methoxybenzylidene)-4-butylaniline (MBBA)	InChI=1S/C18H21NO/c1-3-4-5-15-6-10-17(11-7-15)19-14-16-8-12-18(20-2)13-9-16/h6-14H,3-5H2,1-2H3	13	267.162	Industrial Compound
Adenosine	InChI=1S/C10H13N5O4/c11-8-5-9(13-2-12-8)15(3-14-5)10-7(18)6(17)4(1-16)19-10/h2-4,6-7,10,16-18H,1H2,(H2,11,12,13)/t4-,6-,7-,10-/m1/s1	13	267.097	Metabolite
D + Biotin	InChI=1S/C10H16N2O3S/c13-8(14)4-2-1-3-7-9-6(5-16-7)11-10(15)12-9/h6-7,9H,1-5H2,(H,13,14)(H2,11,12,15)/t6-,7-,9-/m0/s1	13	244.088	Natural Product
Triethoxy-3-(2-imidazolin-1-yl)propylsilane	InChI=1S/C12H26N2O3Si/c1-4-15-18(16-5-2,17-6-3)11-7-9-14-10-8-13-12-14/h12H,4-11H2,1-3H3	13	274.171	Industrial Compound
6-[4-(Methylsulfonyl)phenyl]-2-pyridinecarboxaldehyde	InChI=1S/C13H11NO3S/c1-18(16,17)12-7-5-10(6-8-12)13-4-2-3-11(9-15)14-13/h2-9H,1H3	13	261.046	Industrial Compound
Kinetin	InChI=1S/C10H9N5O/c1-2-7(16-3-1)4-11-9-8-10(13-5-12-8)15-6-14-9/h1-3,5-6H,4H2,(H2,11,12,13,14,15)	13	215.081	Natural Product
Cytidine	InChI=1S/C9H13N3O5/c10-5-1-2-12(9(16)11-5)8-7(15)6(14)4(3-13)17-8/h1-2,4,6-8,13-15H,3H2,(H2,10,11,16)/t4-,6-,7-,8-/m1/s1	13	243.086	Metabolite
2-Hydroxy-5-nitrobenzaldehyde	InChI=1S/C7H5NO4/c9-4-5-3-6(8(11)12)1-2-7(5)10/h1-4,10H	12	167.022	Industrial Compound
4,4',4"-Tri-tert-Butyl-2,2':6',2"-terpyridine	InChI=1S/C27H35N3/c1-25(2,3)18-10-12-28-21(14-18)23-16-20(27(7,8)9)17-24(30-23)22-15-19(11-13-29-22)26(4,5)6/h10-17H,1-9H3	12	401.283	Industrial Compound

Tryptophan	InChI=1S/C11H12N2O2/c12-9(11(14)15)5-7-6-13-10-4-2-1-3-8(7)10/h1-4,6,9,13H,5,12H2,(H,14,15)/t9-/m0/s1	12	204.090	Amino Acid
Dienogest	InChI=1S/C20H25NO2/c1-19-8-6-16-15-5-3-14(22)12-13(15)2-4-17(16)18(19)7-9-20(19,23)10-11-21/h12,17-18,23H,2-10H2,1H3/t17-,18+,19+,20-/m1/s1	12	311.189	Pharmaceutical
3-(3-Methoxyphenyl)thiomorpholine	InChI=1S/C11H15NOS/c1-13-10-4-2-3-9(7-10)11-8-14-6-5-12-11/h2-4,7,11-12H,5-6,8H2,1H3	11	209.087	Industrial Compound
1,1'-(Methylenedi-4,1-phenylene)bismaleimide	InChI=1S/C21H14N2O4/c24-18-9-10-19(25)22(18)16-5-1-14(2-6-16)13-15-3-7-17(8-4-15)23-20(26)11-12-21(23)27/h1-12H,13H2	11	358.095	Industrial Compound
2-(2-Thienyl)isonicotinic acid	InChI=1S/C10H7NO2S/c12-10(13)7-3-4-11-8(6-7)9-2-1-5-14-9/h1-6H,(H,12,13)	11	205.020	Industrial Compound
Chlorquinaldol	InChI=1S/C10H7Cl2NO/c1-5-2-3-6-7(11)4-8(12)10(14)9(6)13-5/h2-4,14H,1H3	11	226.990	Pharmaceutical
Sinnapinic Acid	InChI=1S/C11H12O5/c1-15-8-5-7(3-4-10(12)13)6-9(16-2)11(8)14/h3-6,14H,1-2H3,(H,12,13)/b4-3+	11	224.068	Metabolite
Arginine	InChI=1S/C6H14N4O2/c7-4(5(11)12)2-1-3-10-6(8)9/h4H,1-3,7H2,(H,11,12)(H4,8,9,10)/t4-/m0/s1	10	174.112	Amino Acid
Phenylalanine	InChI=1S/C9H11NO2/c10-8(9(11)12)6-7-4-2-1-3-5-7/h1-5,8H,6,10H2,(H,11,12)/t8-/m0/s1	10	165.079	Amino Acid
Tyrosine	InChI=1S/C9H11NO3/c10-8(9(12)13)5-6-1-3-7(11)4-2-6/h1-4,8,11H,5,10H2,(H,12,13)/t8-/m0/s1	10	181.074	Amino Acid
CDS021753	InChI=1S/C12H18O2/c1-14-11(13)12-5-8-2-9(6-12)4-10(3-8)7-12/h8-10H,2-7H2,1H3	9	194.131	Industrial Compound
Sulfanilamide	InChI=1S/C6H8N2O2S/c7-5-1-3-6(4-2-5)11(8,9)10/h1-4H,7H2,(H2,8,9,10)	9	172.031	Pharmaceutical
5-Aminoisophthalic acid	InChI=1S/C8H7NO4/c9-6-2-4(7(10)11)1-5(3-6)8(12)13/h1-3H,9H2,(H,10,11)(H,12,13)	9	181.038	Industrial Compound

Methionine	InChI=1S/C5H11NO2S/c1-9-3-2-4(6)5(7)8/h4H,2-3,6H2,1H3,(H,7,8)/t4-/m0/s1	8	149.051	Amino Acid
4,4'-Diaminobenzene	InChI=1S/C12H12N4/c13-9-1-5-11(6-2-9)15-16-12-7-3-10(14)4-8-12/h1-8H,13-14H2	8	212.106	Industrial Compound
Proline	InChI=1S/C5H9NO2/c7-5(8)4-2-1-3-6-4/h4,6H,1-3H2,(H,7,8)	8	115.063	Amino Acid
Acontic Acid	InChI=1S/C6H6O6/c7-4(8)1-3(6(11)12)2-5(9)10/h1H,2H2,(H,7,8)(H,9,10)(H,11,12)/b3-1-	7	174.016	Metabolite
Asparagine	InChI=1S/C4H8N2O3/c5-2(4(8)9)1-3(6)7/h2H,1,5H2,(H2,6,7)(H,8,9)/t2-/m0/s1	7	132.053	Amino Acid
Lysine	InChI=1S/C6H14N2O2/c7-4-2-1-3-5(8)6(9)10/h5H,1-4,7-8H2,(H,9,10)/t5-/m0/s1	7	146.106	Amino Acid
Cysteine	InChI=1S/C3H7NO2S/c4-2(1-7)3(5)6/h2,7H,1,4H2,(H,5,6)/t2-/m0/s1	6	121.020	Amino Acid
Ketoglutaric Acid	InChI=1S/C5H6O5/c6-3(5(9)10)1-2-4(7)8/h1-2H2,(H,7,8)(H,9,10)	6	146.022	Metabolite
Oxaloacetic Acid	InChI=1S/C4H4O5/c5-2(4(8)9)1-3(6)7/h1H2,(H,6,7)(H,8,9)	6	132.006	Metabolite
Threonine	InChI=1S/C4H9NO3/c1-2(6)3(5)4(7)8/h2-3,6H,5H2,1H3,(H,7,8)	6	119.058	Amino Acid
Alanine	InChI=1S/C3H7NO2/c1-2(4)3(5)6/h2H,4H2,1H3,(H,5,6)/t2-/m0/s1	6	89.090	Amino Acid
1-(2-Aminoethyl)piperazine	InChI=1S/C6H15N3/c7-1-4-9-5-2-8-3-6-9/h8H,1-7H2	5	129.127	Industrial Compound
Serine	InChI=1S/C3H7NO3/c4-2(1-5)3(6)7/h2,5H,1,4H2,(H,6,7)/t2-/m0/s1	5	105.043	Amino Acid
Succinic Acid	InChI=1S/C4H6O4/c5-3(6)1-2-4(7)8/h1-2H2,(H,5,6)(H,7,8)	5	118.027	Metabolite
Glycine	InChI=1S/C2H5NO2/c3-1-2(4)5/h1,3H2,(H,4,5)	4	75.070	Amino Acid
Pyruvate	InChI=1S/C3H4O3/c1-2(4)3(5)6/h1H3,(H,5,6)/p-1	4	88.060	Metabolite

Supplementary Table 2: Table of molecules included in figure 2B in the main text. Includes the molecular assembly number (MA), the molecular weight (MW) and the categorisation as used in the figure.

8 Example Mass Spectra with Fragmentation

Below are some example mass spectra of the compounds shown in table 1 with the MS1 and MS2 spectra. Full data available on request.

8.1 Single Molecules via SIM

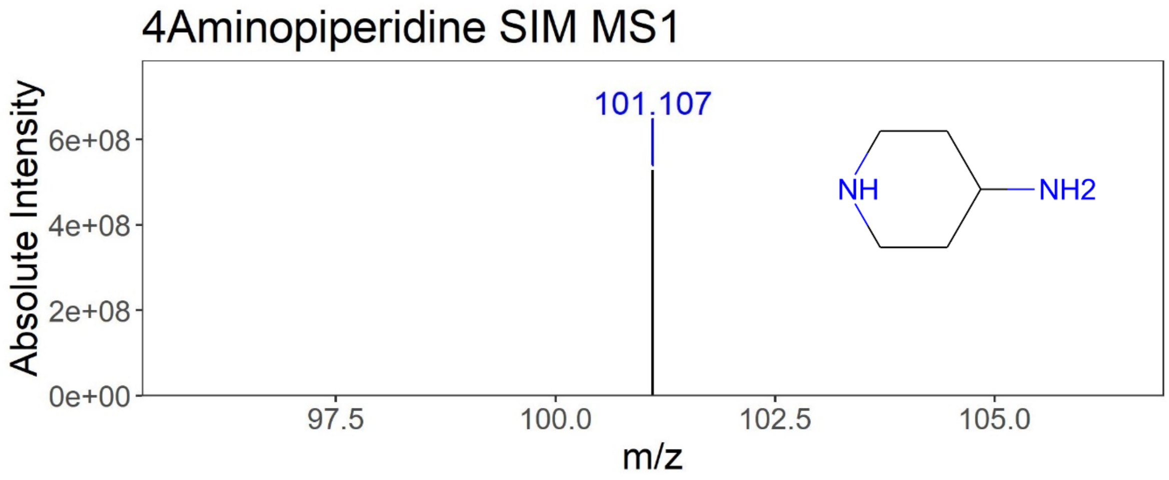

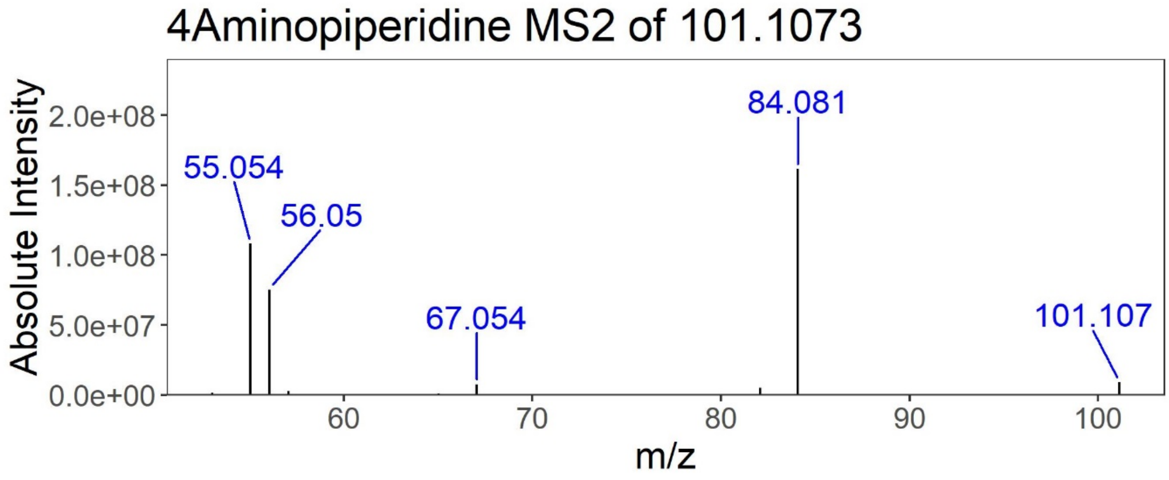

Supplementary Figure 20: MS1 of 4-Aminopiperidine sample (top) with MS2 spectra (bottom). This molecule has a Molecular Assembly Index of 4 and 4 MS2 peaks

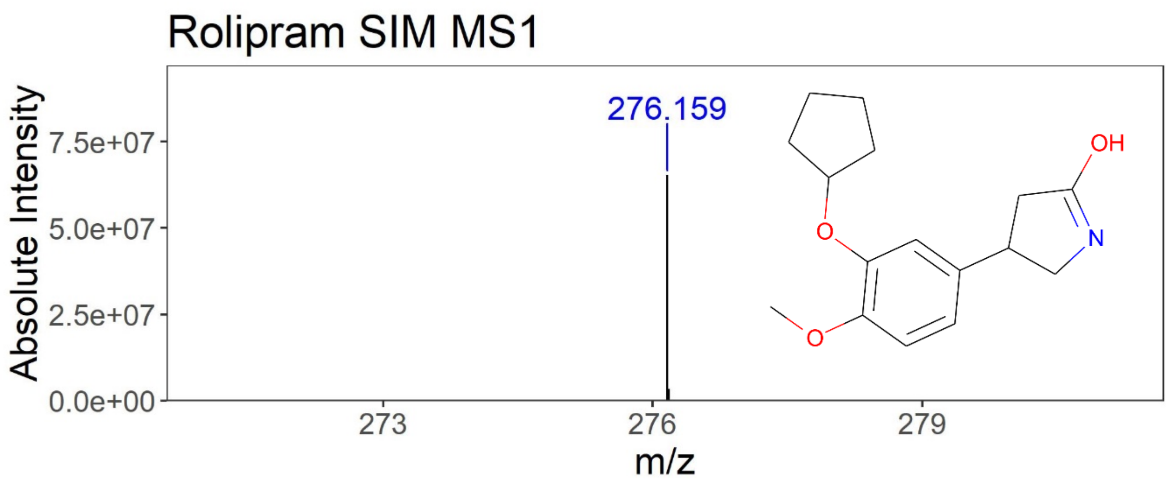

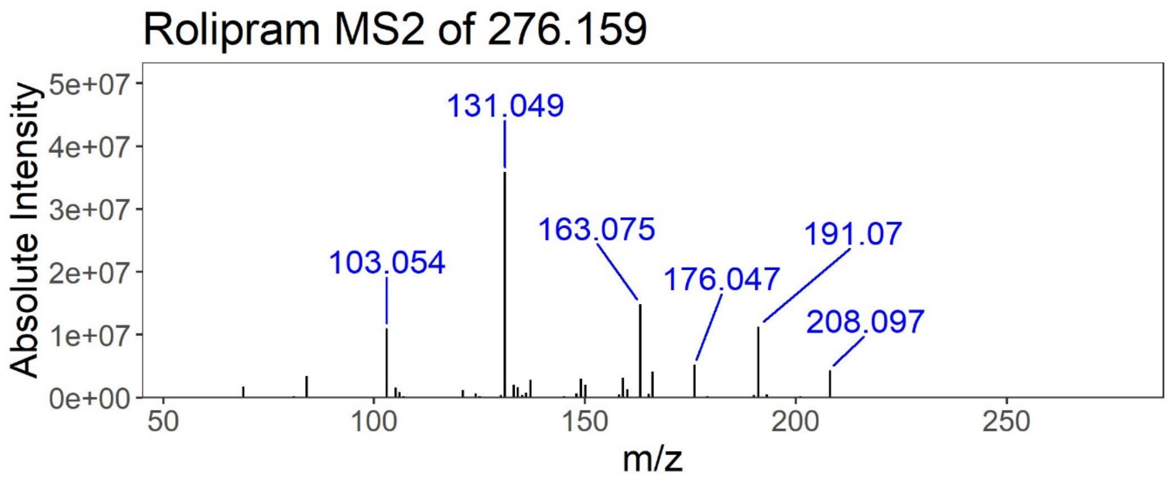

Supplementary Figure 21: MS1 of Rolipram sample (top) with MS2 spectra (bottom). This molecule has a Molecular Assembly Index of 15 and 10 MS2 peaks

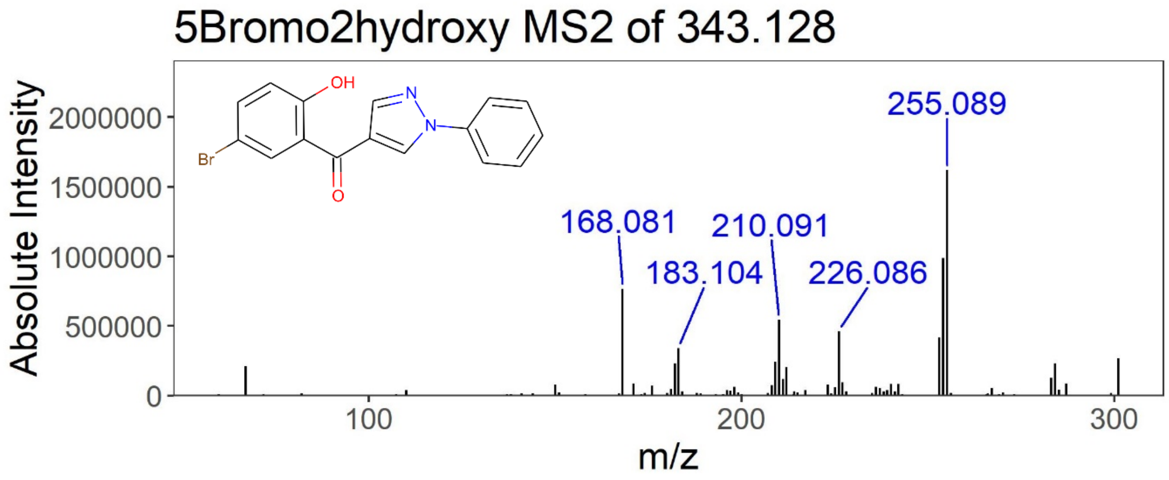

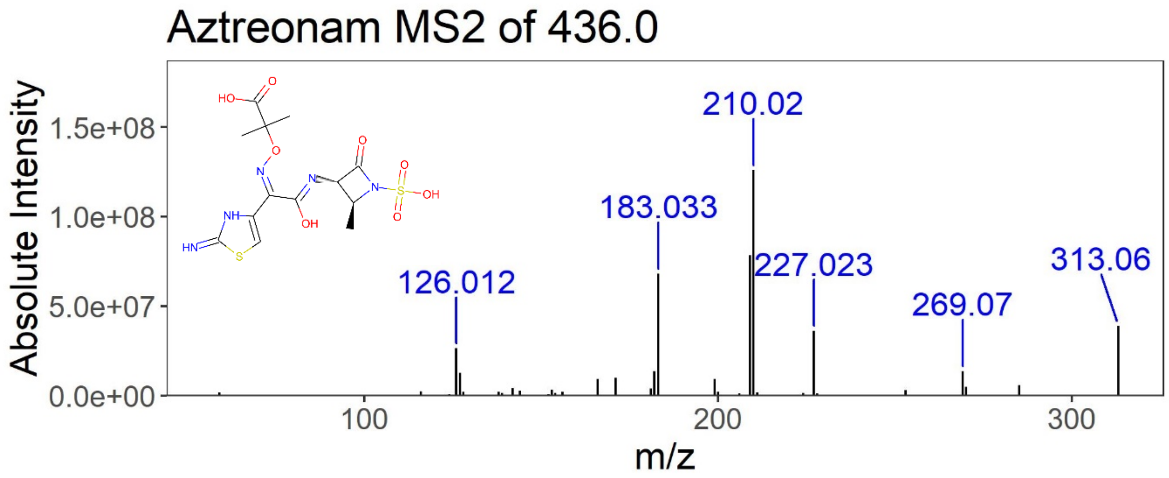

Supplementary Figure 22: MS2 for (5-bromo-2-hydroxy-phenyl)-(1-phenyl-1h-pyrazol-4-yl)ketone (top) and Aztreonam (bottom). (5-bromo-2-hydroxy-phenyl)-(1-phenyl-1h-pyrazol-4-yl)ketone has a Molecular Assembly Index of 16, and 18 MS2 peaks. Aztreonam has a Molecular Assembly Index of 23, and 14 MS2 peaks.

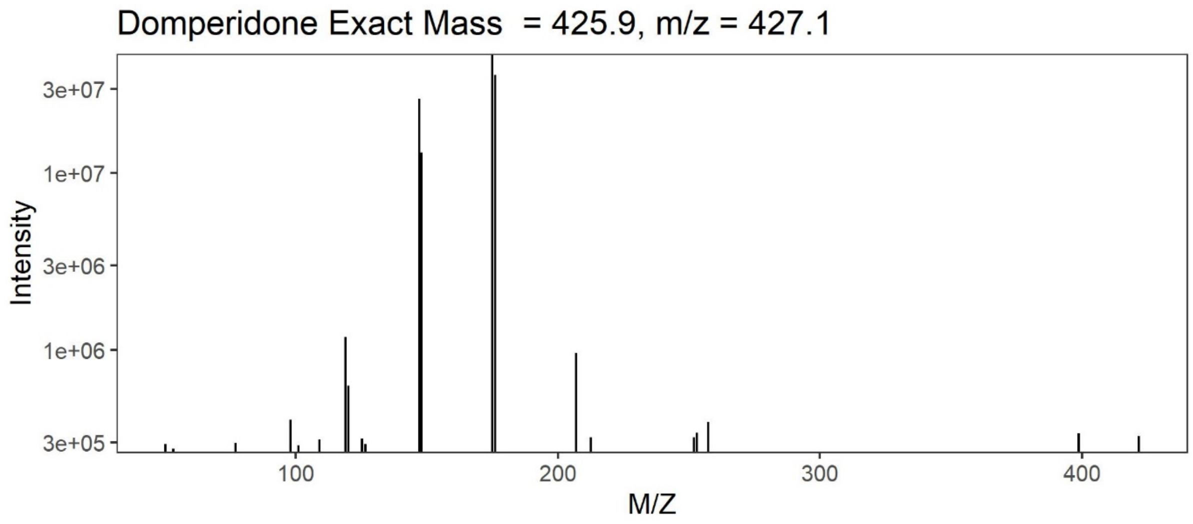

Supplementary Figure 23: MS2 Spectra of Domperidone, Exact Mass of 425.9, m/z of 427.1, and a Molecular Assembly Index of 16, with 23 peaks in the fragmentation spectra.

8.2 Environmental Samples via Data Dependent Acquisition

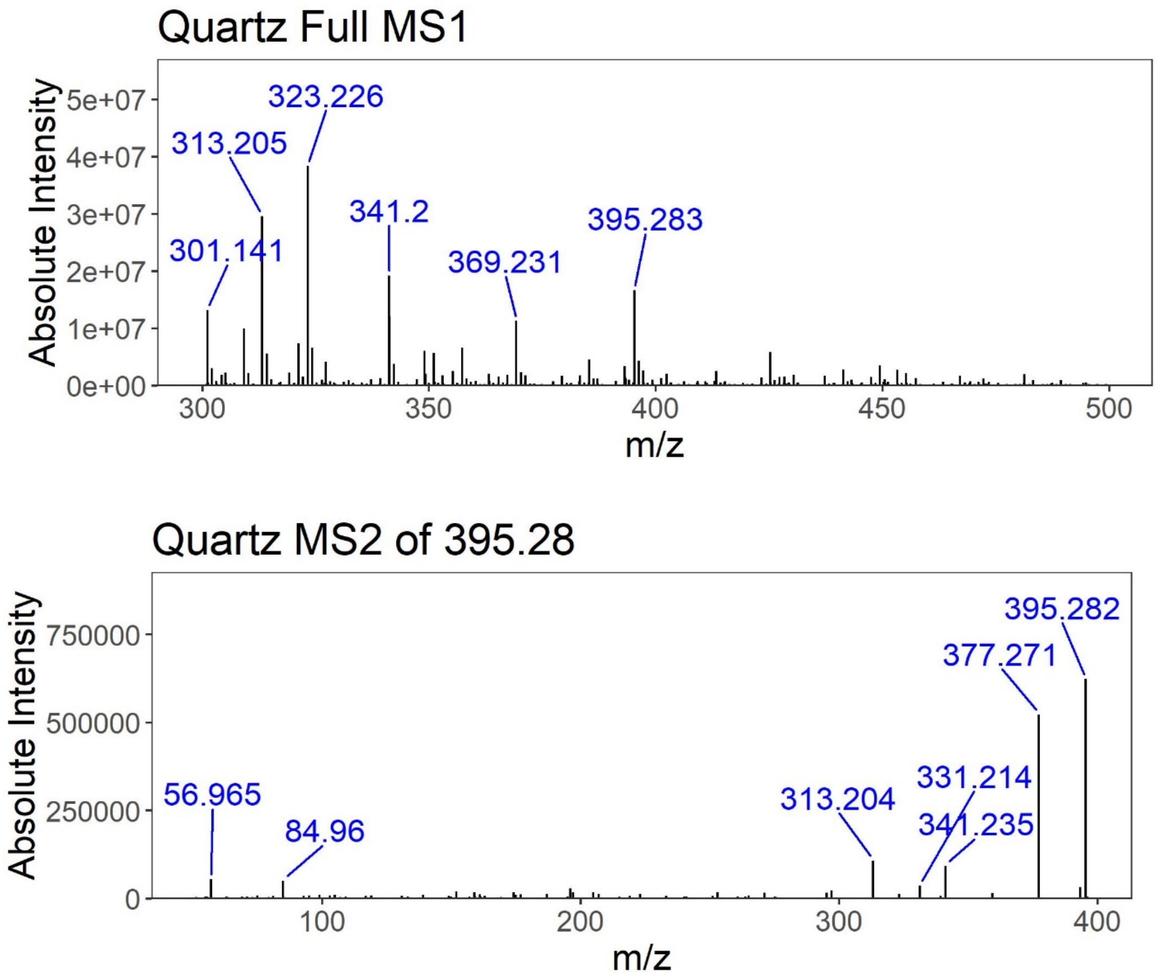

Supplementary Figure 24: Full MS1 (top) and selected (bottom) MS2 spectra from Quartz sample. The highest number of MS2 peaks observed in this sample was 9 and the number of shown in the extracted spectra is 9

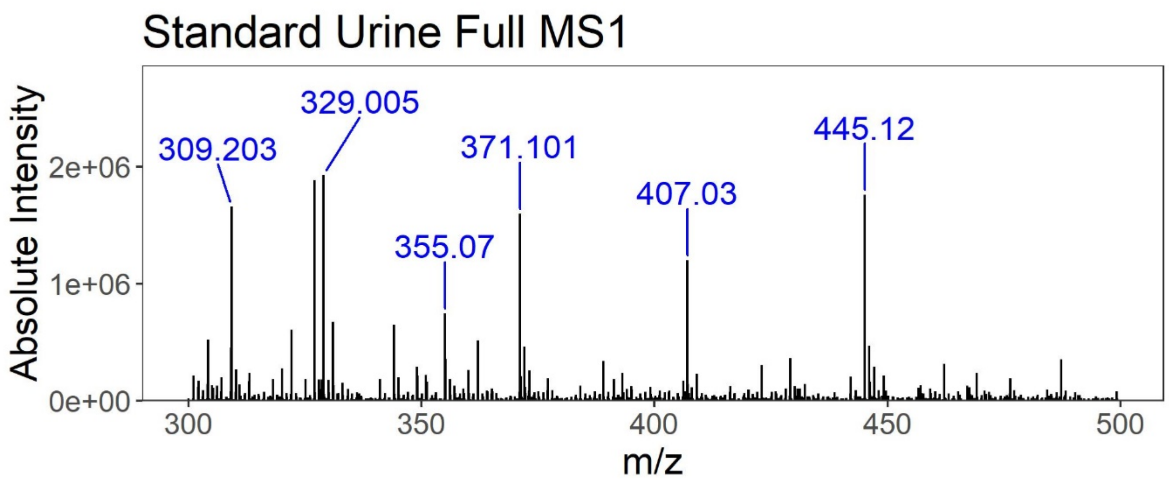

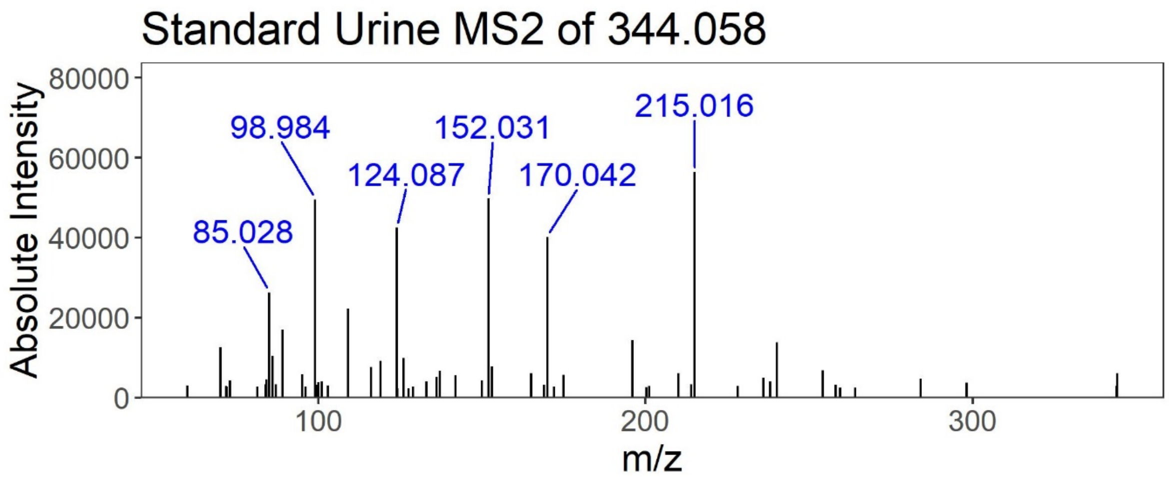

Supplementary Figure 25: Supplementary Figure 24. Full MS1 (top) and selected (bottom) MS2 spectra from Urinary Peptides sample. The highest number of MS2 peaks observed in this sample was 58 and the number of shown in the extracted spectra is 53.

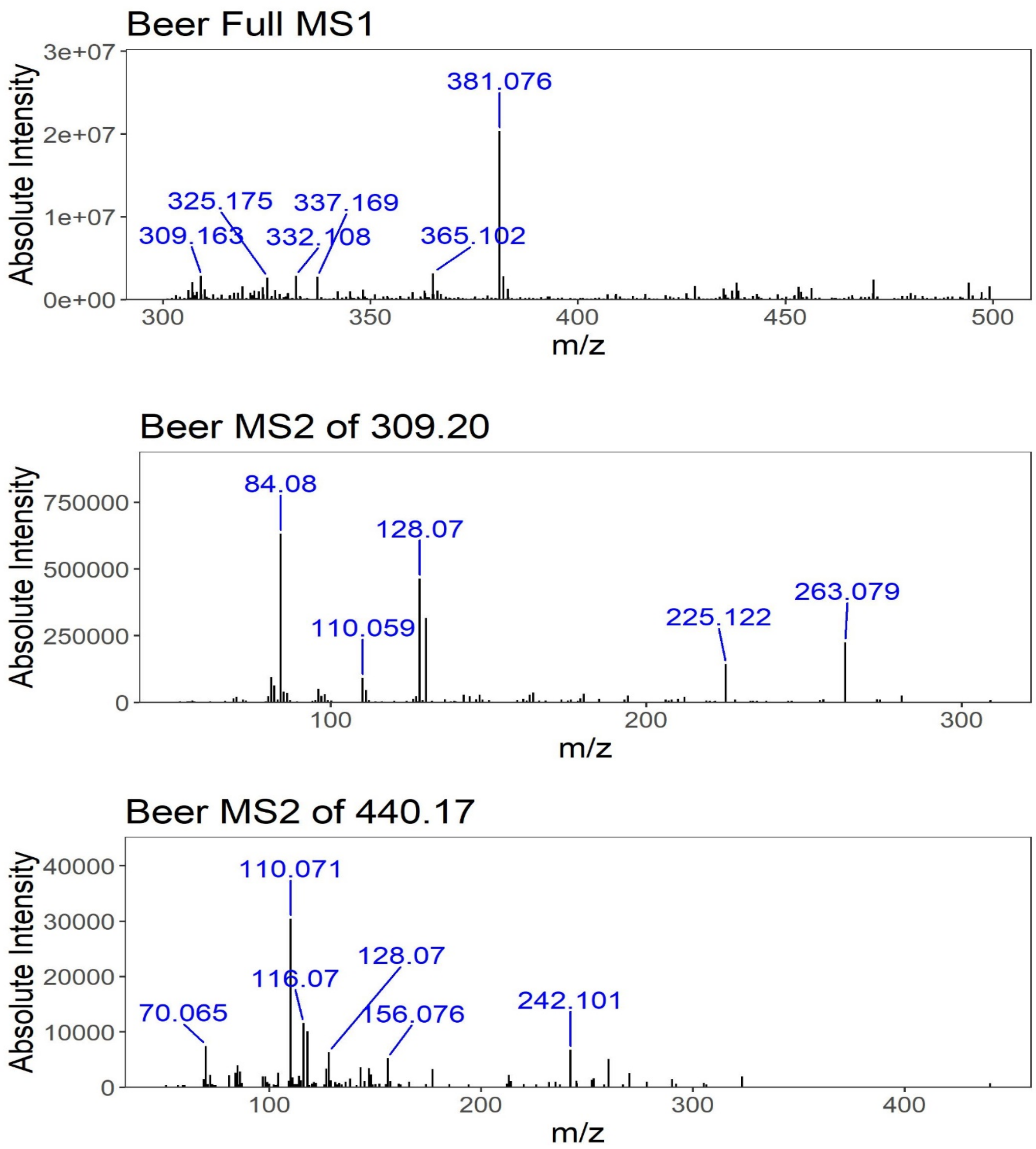

Supplementary Figure 26: Full MS1 (top) and selected (bottom) MS2 spectra from Beer sample. The highest number of MS2 peaks observed in this sample was 83 and the number of shown in the extracted spectra is 17 (middle) and 62 (bottom)

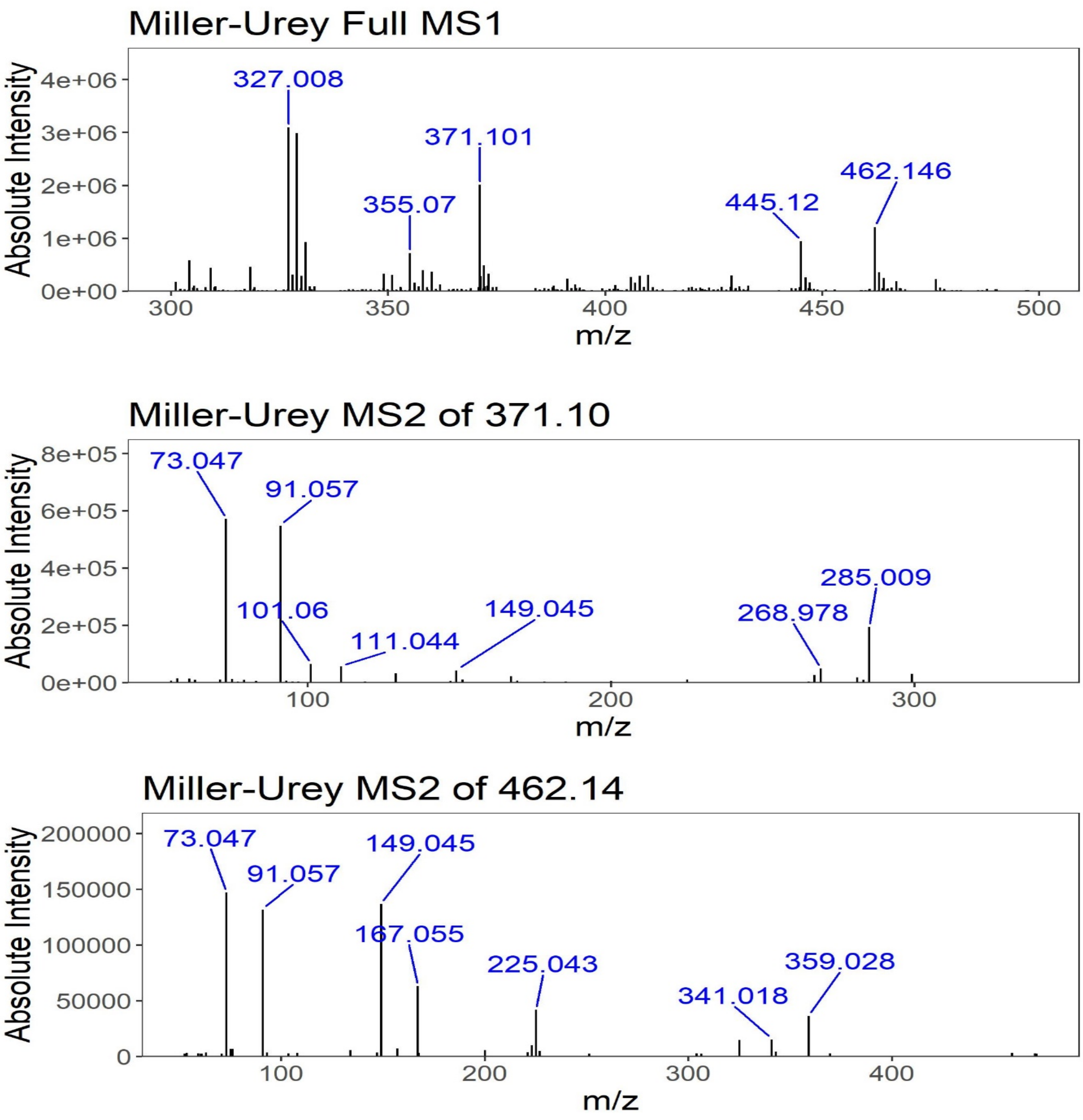

Supplementary Figure 27: Full MS1 (top) and selected (bottom) MS2 spectra from Miller Urey sample. The highest number of MS2 peaks observed in this sample was 16 and the number of shown in the extracted spectra is 10 (middle) and 13 (bottom).

9 Inferring MA with Convolutional Neural Networks

A Convolutional Neural Network (CNN) was generated to predict MA from Mass Spectrometry (MS) data, using the VGG19 (*42*) architecture show in Supplementary Figure 28. The MS Data was extracted as JCAMP-DX files from the NIST webbook (*43*) with a python script. Input images were programmatically generated from the JCAMP-DX files with white peaks of given height and horizontal position on a black background (Supplementary Figure 27). The training dataset comprised 5652 input images, over the MA range of 4 to 11, with a total of 707 images per MA. The labels were one-hot encoded MA indices. For general information on CNNs and one-hot encoding, see (*44*).

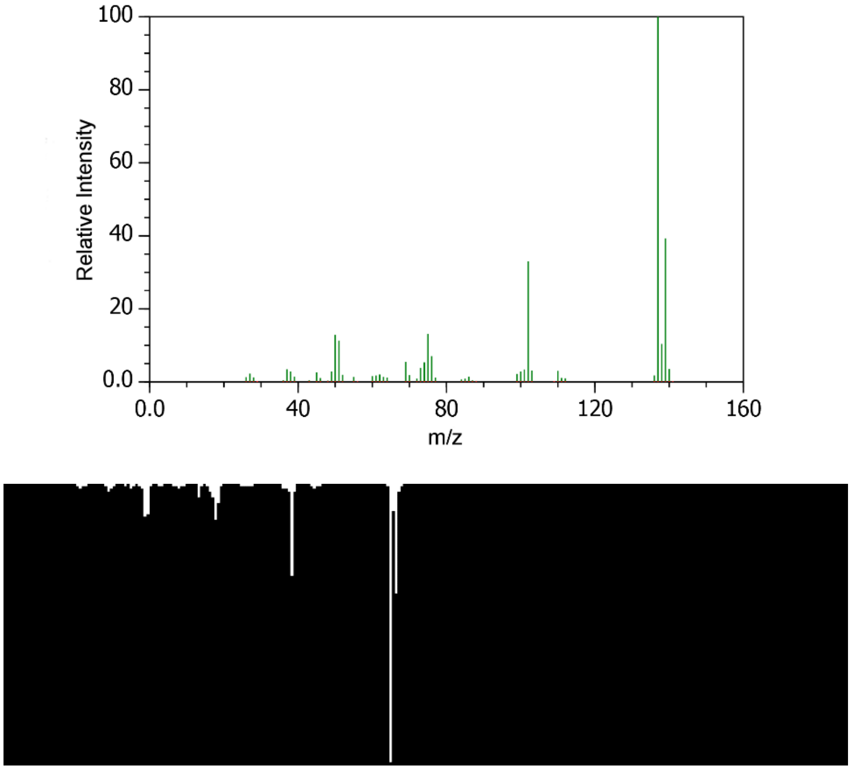

Supplementary Figure 28: Top: Mass spectrum of 4-chlorobenzonitrile. Bottom: Training image used for the MS CNN corresponding to the mass spectrum 4-chlorobenzonitrile.

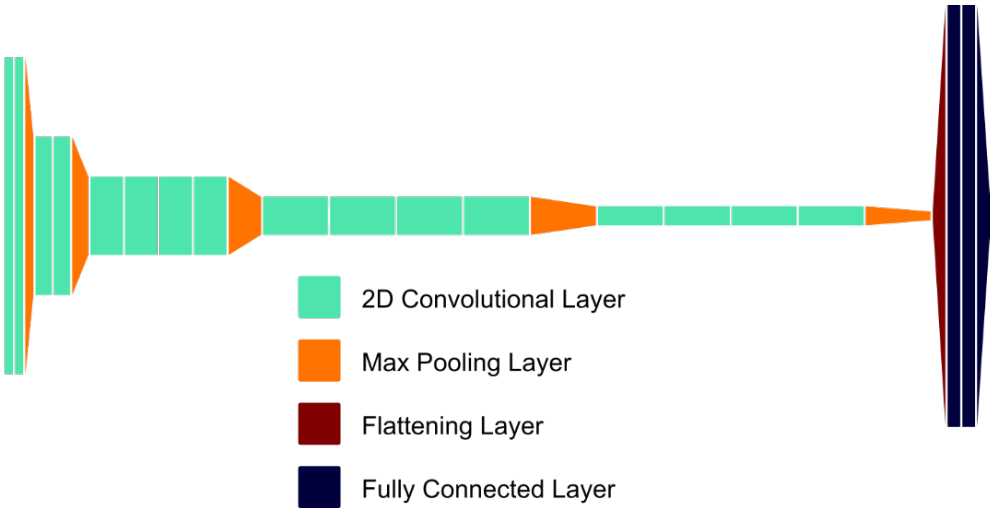

Supplementary Figure 29: Graphical representation of the VGG19 architecture used. The width and height of each block represent the dimensions of the tensor passed into each layer. The height denotes the x and y dimensions, and the depth denotes the z dimension. The fully connected layers are not drawn to the scale of their input tensors, as the input tensors are 1D vectors with a length far greater than the dimensions of the convolutional and max pooling layers.

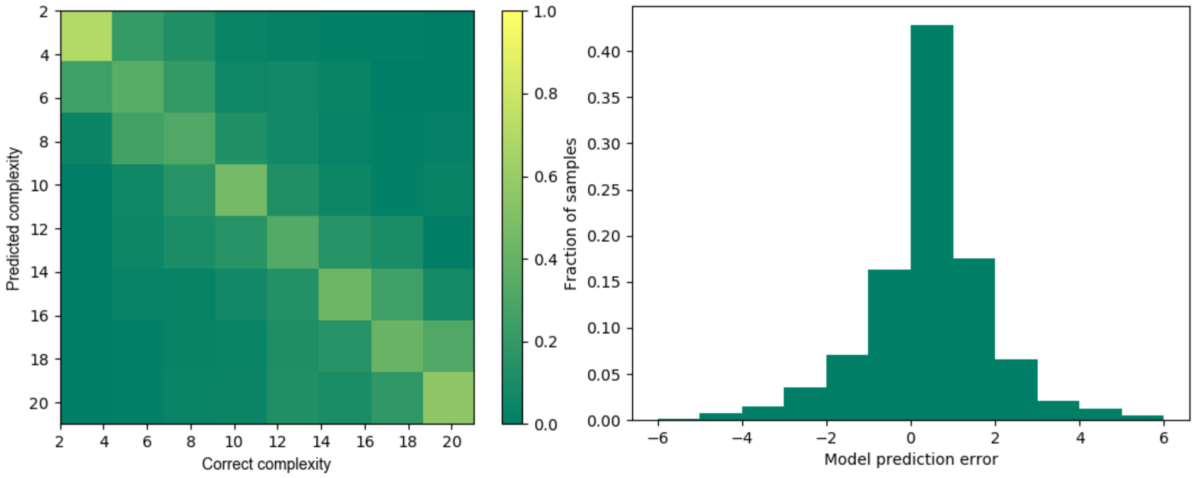

Supplementary Figure 30: Visualisation of model errors on the test dataset for the MS CNN. Left: Confusion matrix showing model errors at different combinations of predicted and correct MA. Right: Histogram of model errors.

Due to the fact that this was a preliminary investigation into the use of MS spectra with Neural Networks to predict MA, the test dataset was also used for validation. In general, this is bad practice as there is significant risk of selecting hyperparameters that exclusively fit the test dataset, but for initial investigation into whether the CNN would be able to predict with any degree of accuracy, leaving out the validation set to allow more data to be used for training seemed the best option. The test/validation dataset consisted of 624 images, with 78 images per MA. There was insufficient data to train the network at a higher MA range than this. Training was carried out until the test/validation MAE stopped decreasing, 67 epochs in total. The training dataset was updated every three epochs with new MS spectra not contained in the test/validation dataset.

The confusion matrix and error histogram in Supplementary Figure 29 show that the MS CNN managed to predict MA with some degree of accuracy. The maximum absolute error was 6, with a maximum possible absolute error of 7, and the test percentage of perfect guesses for the model was 42.8%. Other models based on SMILES strings and images of molecular structures performed somewhat better, however the MS based model was limited in accuracy and MA range by the limited amount of training data available, and improved performance could be possible with more training data.

10 Supplementary References

1. D. J. Des Marais, M. R. Walter, Astrobiology: Exploring the Origins, Evolution, and Distribution of Life in the Universe. *Annual Review of Ecology and Systematics* **30**, 397-420 (1999).
2. D. J. Des Marais *et al.*, The NASA Astrobiology Roadmap. *Astrobiology* **8**, 715-730 (2008).
3. S. I. Walker *et al.*, Exoplanet Biosignatures: Future Directions. *Astrobiology* **18**, 779-824 (2018).
4. E. Schwieterman *et al.*, Exoplanet Biosignatures: A Review of Remotely Detectable Signs of Life. *Astrobiology* **18**, 663-708 (2018).

5. C. D. Georgiou, D. W. Deamer, Lipids as Universal Biomarkers of Extraterrestrial Life. *Astrobiology* **14**, 541-549 (2014).
6. S. Benner, Detecting Darwinism from Molecules in the Enceladus Plumes, Jupiter's Moons, and Other Planetary Water Lagoons. *Astrobiology* **17**, 840-851 (2017).
7. A. J. MacDermott *et al.*, Homochirality as the signature of life: the SETH Cigar. *Planetary and Space Science* **44**, 1441-1446 (1996).
8. R. Breslow, M. S. Levine, Amplification of enantiomeric concentrations under credible prebiotic conditions. *Proceedings of the National Academy of Sciences* **103**, 12979-12980 (2006).
9. A. D. Anbar, Iron stable isotopes: beyond biosignatures. *Earth and Planetary Science Letters* **217**, 223-236 (2004).
10. M. Neveu, L. E. Hays, M. A. Voytek, M. H. New, M. D. Schulte, The Ladder of Life Detection. *Astrobiology* **18**, 1375-1402 (2018).
11. D. Minoli, Combinatorial Graph Complexity. *Atti della Accademia Nazionale dei Lincei* **59**, 651-661 (1975).
12. D. Bonchev, N. Trinajstić, Information theory, distance matrix, and molecular branching. *The Journal of Chemical Physics* **67**, 4517-4533 (1977).
13. A. T. Balaban, Topological indices based on topological distances in molecular graphs. *Pure and Applied Chemistry* **55**, 199 (1983).
14. J. R. Proudfoot, A path based approach to assessing molecular complexity. *Bioorganic & Medicinal Chemistry Letters* **27**, 2014-2017 (2017).
15. G. Rücker, C. Rücker, Walk Counts, Labyrinthicity, and Complexity of Acyclic and Cyclic Graphs and Molecules. *Journal of Chemical Information and Computer Sciences* **40**, 99-106 (2000).
16. M. Randić, D. Plavšić, On the Concept of Molecular Complexity. *Croatica Chemica Acta* **75**, 107-116 (2002).
17. Q. Zhang, C. Wu, J. Suo, Y. Zhou, L. Xu, Development of a highly selective molecular topological index. *Journal of Chemometrics* **30**, 70-74 (2016).
18. G. Rücker, C. Rücker, Substructure, Subgraph, and Walk Counts as Measures of the Complexity of Graphs and Molecules. *Journal of Chemical Information and Computer Sciences* **41**, 1457-1462 (2001).
19. M. von Korff, T. Sander, Molecular Complexity Calculated by Fractal Dimension. *Scientific Reports* **9**, 967 (2019).
20. S. H. Bertz, The first general index of molecular complexity. *Journal of the American Chemical Society* **103**, 3599-3601 (1981).
21. T. Böttcher, An Additive Definition of Molecular Complexity. *Journal of Chemical Information and Modeling* **56**, 462-470 (2016).
22. R. Barone, M. Chanon, A New and Simple Approach to Chemical Complexity. Application to the Synthesis of Natural Products. *Journal of Chemical Information and Computer Sciences* **41**, 269-272 (2001).
23. T. K. Allu, T. I. Oprea, Rapid Evaluation of Synthetic and Molecular Complexity for in Silico Chemistry. *Journal of Chemical Information and Modeling* **45**, 1237-1243 (2005).
24. C. W. Coley, L. Rogers, W. H. Green, K. F. Jensen, SCScore: Synthetic Complexity Learned from a Reaction Corpus. *Journal of Chemical Information and Modeling* **58**, 252-261 (2018).
25. J. Li, M. D. Eastgate, Current complexity: a tool for assessing the complexity of organic molecules. *Organic & Biomolecular Chemistry* **13**, 7164-7176 (2015).
26. R. P. Sheridan *et al.*, Modeling a Crowdsourced Definition of Molecular Complexity. *Journal of Chemical Information and Modeling* **54**, 1604-1616 (2014).

27. S. M. Marshall, A. R. G. Murray, L. Cronin, A probabilistic framework for identifying biosignatures using Pathway Complexity. *Philosophical Transactions of the Royal Society A: Mathematical, Physical and Engineering Sciences* **375**, 20160342 (2017).
28. S. M. Marshall, D. Moore, A. R. G. Murray, S. I. Walker, L. Cronin, Quantifying the pathways to life using assembly spaces. *Arxiv*, arXiv:1907.04649 (2019).
29. S. R. Heller, A. McNaught, I. Pletnev, S. Stein, D. Tchekhovskoi, InChI, the IUPAC International Chemical Identifier. *Journal of Cheminformatics* **7**, 23 (2015).
30. A. J. Lawson, J. Swienty-Busch, T. Géoui, D. Evans, in *The Future of the History of Chemical Information*. (American Chemical Society, 2014), vol. 1164, chap. 8, pp. 127-148.
31. C. Benecke, T. Grüner, A. Kerber, R. Laue, T. Wieland, MOLecular structure GENeration with MOLGEN, new features and future developments. *Fresenius' Journal of Analytical Chemistry* **359**, 23-32 (1997).
32. R. Adusumilli, P. Mallick, in *Proteomics: Methods and Protocols,* L. Comai, J. E. Katz, P. Mallick, Eds. (Springer New York, New York, NY, 2017), pp. 339-368.
33. S. Colón-Santos, G. J. T. Cooper, L. Cronin, Taming the Combinatorial Explosion of the Formose Reaction via Recursion within Mineral Environments. *ChemSystemsChem* **1**, e1900014 (2019).
34. G. J. T. Cooper *et al.*, Miller–Urey Spark-Discharge Experiments in the Deuterium World. *Angewandte Chemie International Edition* **56**, 8079-8082 (2017).
35. D. P. Glavin, A. S. Burton, J. E. Elsila, J. C. Aponte, J. P. Dworkin, The Search for Chiral Asymmetry as a Potential Biosignature in our Solar System. *Chemical Reviews* **120**, 4660-4689 (2020).
36. Y. Huang, J. C. Aponte, J. Zhao, R. Tarozo, C. Hallmann, Hydrogen and carbon isotopic ratios of polycyclic aromatic compounds in two CM2 carbonaceous chondrites and implications for prebiotic organic synthesis. *Earth and Planetary Science Letters* **426**, 101-108 (2015).
37. S. A. Wise, M. M. Schantz, D. L. Poster, M. J. Lopez de Alda, L. C. Sander, in *Techniques and Instrumentation in Analytical Chemistry,* D. BarcelÓ, Ed. (Elsevier, 2000), vol. 21, pp. 649-687.
38. S. A. Wise *et al.*, Two new marine sediment standard reference materials (SRMs) for the determination of organic contaminants. *Analytical and Bioanalytical Chemistry* **378**, 1251-1264 (2004).
39. E. Zaikova *et al.*, Antarctic Relic Microbial Mat Community Revealed by Metagenomics and Metatranscriptomics. *Frontiers in Ecology and Evolution* **7**, (2019).
40. A. R. Lewis *et al.*, Mid-Miocene cooling and the extinction of tundra in continental Antarctica. *Proceedings of the National Academy of Sciences* **105**, 10676-10680 (2008).
41. Wang, D. MTBLS1411: Hfq Regulates Efflux Pump Expression and Purine Metabolic Pathway to Increase the Trimethoprim Resistance in Aeromonas veronii (2019). https://www.ebi.ac.uk/metabolights/MTBLS1411/descriptors. (Accessed: 1st May 2020)
42. K. Simonyan, A. Zisserman, Very Deep Convolutional Networks for Large-Scale Image Recognition. *Arxiv*, arXiv:1409.1556 (2014).
43. W. E. Acree Jr., J. S. Chickos, in *NIST Chemistry WebBook, NIST Standard Reference Database Number 69,* P. J. Linstrom, W. G. Mallard, Eds. (National Institute of Standards and Technology, Gaithersburg MD).
44. C. Bishop, *Pattern Recognition and Machine Learning*. (Springer-Verlag New York, 2006).
