# Supplementary material for: Identifying molecules as biosignatures with assembly theory and mass spectrometry
Source: Nat Commun. 2021 May 24;12:3033. doi: 10.1038/s41467-021-23258-x (PMC8144626; doi:10.1038/s41467-021-23258-x)
Supplement: Supplementary file 2 — Source Data File [file 41467_2021_23258_MOESM2_ESM.zip › 287799_0_related_ms_5137685_qkpymq.pdf]

The present manuscript´s aim is in the use of direct injection mass spectrometry combined with a dedicated data processing to find "biosignatures". While I feel the approach is interesting in general it is not convincing in details…

The manuscript presents a new theory, exploration of chemical space, experimental validation, then proof of the technique with real samples. Our supporting information includes an extensive SI, and this precisely describes the basis for the new theory, the algorithm, the profiling of chemical space, the benchmarking, the molecular data, the environmental samples. The code is given for both the algorithm and the data processing of the MZ fragmentation data and also the data processing for each of the figures. The data submitted runs to 1.2 Gb with read me files that allows the faithful and precise reproduction of the figures.

…and does not show the breakthrough research/results I´m expecting in the journal Nature.

To be clear the manuscript presents an entirely new framework for life detection. This includes a hypothesis and a new theory, the benchmarking data, the calibration curves and then tests on samples showing that molecular complexity can be determined using mass spec fragmentation.

The paper is positioned in the field of astrobiology and the authors´s goal is to define a "proxy" for biosignatures as extracted from in situ analysis using mass spectrometry.

This is inspired by the fact that the number one tool being sent off Earth to Mars, Europa and Titan are mass spectrometers and this presents a new approach to use these instruments.

The concept of "life" or "living systems" would have merited more references and definition to enable to define the significance of what can be a "biosignature".

We have given references in the manuscript and we also describe the current state of the art which explains that there is no current universal definition of life which cause ambiguity with exploring biosignatures (e.g. phosphine on Venus). Hence the main assertion of the manuscript is that a new approach could be used that instead looks at intrinsic molecular complexity, but can intrinsic molecular complexity be measured? We show it can and hence present the first new theory, experiment, and analysis showing that molecular complexity can be quantified and measured.

As the authors compare existing terrestrial biotic and abiotic systems, isn´t their approach biased in a way from our very focused knowledge on life on Earth? What could be the bases of life elsewhere, if?

Our thesis is that complex molecules can be associated with living systems and we go further – suggesting there is a scale of molecular complexity, <u>which is an intrinsic property to all molecules</u>, and we demonstrate this measurable and defines a scale. Our approach only makes one assumption, that life in the Universe will need to evolve from atoms, molecules, and polymers. If that is the case, then our technique will find it – just based on the MA of the molecules. This is a major new direction that will allow us to search for life in the solar system using mass spec alone and to make use of the new instruments going to Titan, Mars, and now probably Venus(!).

Also the point of terrestrial impurity, being the major problem in extraterrestrial samples has not been mentioned in details – especially with the Murchison sample I would have expected many biosignatures as coming from terrestrial contaminations; this could however reflect well a real case in which biosignatures are superimposed to a major abiotic organic profile.

We thank the reviewer for this comment and assure them we also worried about contamination. However, in our extensive analysis, we did not see any evidence of significant biological contamination from Murchison; we note that NASA provided us the sample and had already checked it extensively for impurities. The NASA samples were all double blinded to see if our approach worked. We did many repeats and analyzed the data carefully. The fact that our system did not see any impurities, yet passed the double-blinded tests from

NASA, show help allay the concerns of the referee. We should note our approach was able to find evidence of 'life' in a sea water sample that tested negative using conventional techniques for finding evidence of life. The main point is that in all our data we showed no effects or interference from contamination. Indeed, we think this is a very positive aspect of our technique – that it is resistant to false positives and contamination. We should also note that it is possible to remove molecular complexity by heating the sample and this was demonstrated with the yeast experiments.

The technique was specifically designed to be cautious and hence we accept there will be some false negatives – that is complex molecules that may not be found using the technique – this is a feature of our data processing to ensure consistency and transparency in data processing across ALL the samples.

The sensitivity of our technique to biological processes is an interesting question. We have demonstrated that obvious biological samples like cultured yeast, beer and E. coli are easily detected. Further we used samples from ancient paleo-mats (30,000 and 14 Million years old) to demonstrate that some high MA molecules can be detected in long dead (but well preserved) samples. This in addition to our analysis of seawater demonstrates that our technique is sensitive to even trace evidence of life. Analyzing the sensitivity of our approach would make for an important follow-up study which will be critical for any mission design. We could of course keep trying with other samples.

The wording of molecular assembly theory was defined from the authors and only presented in one of their peer reviewed paper and one on Arxiv (but 2019).

Our theory for assembly theory is a preprint, and the theory of molecular assembly has never been published before. We apologize if the referee thinks this has been published before but note that preprints etc. are not considered as prior art. The idea for assembly theory was present before (as a concept under a different name in our 2017 paper but only to abstract objects, not molecules) but this was in general and not mathematically presented or applied to molecules. It was an abstract concept at that time. Our theory here shows how molecules can have intrinsic complexity, and this can be experimentally measured.

While the authors involved mass spectrometry, they did not detail their choice of ionization source in the main text – the use of a Nanomate is described in materials and methods. ESI is mainly use for polar molecules and blind for oxygen poor and apolar compounds; this point and the need of more universal detectors was not raised by the authors.

The reasoning is given in the SI, and we opted to keep this in the SI to help the readability of the paper. Also, we should point out that the point of this work is to show it is possible to infer MA from MS and we ensured that we used one technique to establish the framework. Work on flight ready mass specs is ongoing and there is no reason to suggest that a different ionization technique is not going to allow the same results to be obtained given that the high resolution fragmentation of complex molecules is well known.

We note the issue with polar molecules hence we opted to make the test as hard as possible by keeping to one source. Our correlation is very good despite this and we not that our 100 test molecules has a good spread of polarity. The key with this manuscript is to establish that the approach works and we should also mention we designed the technique to be resistant to false positives, but in doing so we had to accept that false negatives will be a feature. Despite this very high bar, we have demonstrated that the technique works. Again, exploring the optimal choice for a specific life detection mission would be an interesting and important follow-up study.

Also related to the used mass spectrometry, the concept of resolution and mass precision, essential when analyzing complex mixtures is missing on the main document. The authors should think about the minimal mass resolution and precision needed to "identify" the correct elementary composition (involving the various elements of life i.e. C, H, N, O, P, X(?) and their isotopes in a chosen abundance).

As the reviewer eludes to earlier in their report, it is important we don't make any assumptions about what we are looking for in terms of elemental composition. We are just concerned with the intrinsic complexity of the molecule as determined by the fragmentation. In addition, the important thing about the technique is that we don't need to identify any elementary composition – the MA maps as a function of the number of fragments. This a key point of our approach meaning it can search for highly complex and unknown molecules. We can add further details about the resolution, but we want to assure the reviewer that we use identical instrument settings for all the samples. This is a very hard requirement and hence demonstrates the exceptional robustness of our approach.

The resolution values of 200.000 of the Orbitrap instrument used in the study (as indicated in supplementary information), enables a right composition attribution in complex multi-element samples ONLY up to a mass range of m/z 300. Furthermore because the authors chose the mass range 300 to 500 this resolution is already out of the range to result in correct elementary compositions of the MS1 – this is even more true if considereing extremely complex abiotic samples (the world records in the few hundreds of formula found in a single nominal mass in Petroleomics may be a good example to illustrate this essential problem).

There is no problem here as the MS fragmentation is used to identify the number of fragments and our extensive data shows this works. Again, the technique does not require us to identify the sample, and only ensure that we are not counting fragments from isobaric species. Our data processing algorithm has a filtering step that ensures this is minimized. By requiring a product ion to be present in at least 25% of MS2 spectra assigned to a parent mass we further reduce the chance of random co-fragmentation. It is also worth noting that in our analysis of environmental samples included complex mixtures such as *E-Coli* and yeast in the biotic samples and Murchison in the abiotic samples. All samples analyzed with the same mass spectrometry method were correctly classified in terms of samples that evidenced life and those which did not.

The choice of the fragmentation/collision energy is not clear to me: in routine bioanalysis these values are always adapted to the nature of the compounds to be fragmented – in the case of one value only, one may be blinded for many structures resulting consequently in fully different MA value.

We are aware of this issue and in routine bioanalysis of a particular analyte this is true, it would be proper to fully optimize the instrumentation for the analyte of interest. However, in proteomics this is not the case due to the vast complexity of most analysis. For example, tissue proteomics which rely on long (8hr) LC-MSMS runs are not repeated with different collision energies to ensure complete fragmentation of as many peptides as possible despite peptides fragmenting at different energies. We investigated the effect of collision energies on the known molecules selected for our standard curve. As expected at higher collisions there are more MS2 product ions, however at the energy selected we erred on the side of caution with some molecules not able to fragment completely. In the environmental samples this would lead to false negatives which we have already accepted as part of our approach.

Another problem in my eyes is the direct injection of the samples (even if that would be in the highest needed resolution) because behind every signal of one particular formula one will find many isomers and the number in structures of these isomers would give various amounts of MS2 signals accordingly. In complex materials this number of isomers would be higher compared to biological samples.

Our algorithm and data processing approach did not encounter any problems even with Murchison and we are happy to show more data to demonstrate this. As described previously our post-processing algorithm was designed to handle this situation by requiring that product ions be present in at least 25% of the fragmentation spectra.

While I see the importance in profiling various samples in their origin (as for various sediments of meteorites), I do not see the importance of whisky or beer in this particular context.

Both whisky and beer are derived from fermentation which means that molecules with a high MA could be present but since the alcohol in whisky is distilled then the MA should be low and only whisky that has been stored in casks for a long period of time were expected to sequester high MA molecules from the surroundings. The data did indeed confirm these ideas and hence we think that this is an important test of our approach.

The authors show they have analyzed 100 selected organic compounds – the field of metabolomics is growing of MS2 databases that could have being used as well – this was even not mentioned (HMDB, METLIN, Global Natural Product Social Molecular Networking (GNPS) or KEEG to cite only few important of them).

We have analyzed over 2.5 M molecules for MA theoretically. We have been expanding our compound list, but we thought it important to keep the data consistent. We will be expanding this in future work to help map all of biochemical life on earth. However, the current dataset has produced statistically meaningful results with a very good correlation.

Further, we have added data from our lab which uses spectra from the NIST database and a convolutional neural network to demonstrate how the MA can be inferred from the spectra using machine learning methods - this is around 5600 extra molecules with mass spec and MA data.

Following up the various points in my review I would not consider this manuscript for the journal nature. I could imagine that in implementing several details in the work presented it could be sent to an astrobiology focused journal.

We are glad the referee thinks the work is publishable and therefore technically valid. We are happy to address any of the minor details outlined above. We hope that the referee now understands our theory and algorithm have not been presented before (beyond the non-peer reviewed preprint).
