# Supplementary material for: Identifying molecules as biosignatures with assembly theory and mass spectrometry
Source: Nat Commun. 2021 May 24;12:3033. doi: 10.1038/s41467-021-23258-x (PMC8144626; doi:10.1038/s41467-021-23258-x)
Supplement: Supplementary file 2 — Source Data File [file 41467_2021_23258_MOESM2_ESM.zip › 287799_0_data_set_5137918_qkq38v.zip!/molecular-complexity-data/molecular-complexity-data/README.html]

# This folder contains all the information and code required to recreate the figures from ‘Identifying Molecules as Biosignatures with Assembly Theory and Mass Spectrometry’ by Stuart M. Marshall, Cole Mathis, Emma Carrick, Graham Keenan, Geoffrey J. T. Cooper, Piotr S. Gromski, Heather Graham and Leroy Cronin (2020)

## Code

The majority of the analysis (conceptually) is done with a series of Jupyter notebooks that use a combination of R v3.5 and Python 3. There is also an R script to analyze the results of single molecule MS experiments and some python scripts to analyze the complex mixture MS experiments.

### Molecular Assembly Indices

Molecular assembly indices are used throughout the different data sources. All these values were computed using C++ code written locally at the University of Glasgow. MA indices can be computed using an executable provided in "molecular\_assembly\_algorithm/complexity.exe" which takes as an argument a .mol file containing the molecular structure of interest, for example we've include cubane in the same directory:

```
:\molecular_assembly_algorithm>complexity.exe cubane.mol
File Name: cubane.mol

COMPLETE - complexity 5
process took 1.00942 seconds
```

We often use InChI strings to record/track the structure of molecules. We use RDKit to convert InChI strings to .mol files in order to compute MA.

### Figure 1

To simulate the probabilistic assembly of molecules we developed code in R. The code to regenerate our results is in the directory 'Figure 1- Probabilistic Assembly', there's an R script file as well as a jupyter notebook to manipulate the simulations.

### Figures 3

The main purpose of this code is to read .mzML files and determine the number of MS2 peaks associated with each parent mass. The number of MS2 peaks is then correlated with ions of known MA to produce a Standard Curve (Figure 3C in the main text).

The R script "Figure 3 - Single Molecules/MS2\_counter\_stable.R" can be used to analyze large batches of mzML files and outputs data in a CSV containing information on the number of MS2 peaks for various different parent masses and noise filtering parameters. We've selected the parent mass closest to the exact mass + 1.0 of the molecule and we've used a threshold of 0.01 and a noise floor of 2000 for the data. All of the processed data is in "Figure 3 - Single Molcules/MS\_data\_Standard\_Curve". The fit regression is saved as an R linear model object as "Figure 3 - Single Molcules/ms2\_peaks\_lm\_rq\_combined"

### Figure 4

We've processed the data for the mixtures slight differently, due to their complexity (and therefore size of the data files) and due to the difference in noise expected from a complex mixture compared to a single compound. The scripts to process the data are saved in a zip file called "Mixtures/carrickextractors-master.zip" and they contain their own documentation and explaination. The processed data from all samples is in the file "EnviromentalSamplesUpdated\_Feb2020.csv", which is in a "tidy-long" format, where each parent mass from each sample has it's own row. That standard curve from Figure 3 is then used to predict the MA of ions in mixtures/environmental samples based on the number of MS2 peaks observed in those samples (Figure 4 in the main text).

### Dependencies

#### Python

The python components of the code make use of Pandas, numpy, and RDKit. Pandas and pubchempy can be installed via pip, installing RDKit is best done using Conda, following the instructions on the RDKit website in recommended. https://www.rdkit.org/docs/Install.html

#### R v3.5

The R components use the Tidyverse packages (https://www.tidyverse.org/), the MSnbase package for managing MS data (note this package is under development, http://lgatto.github.io/MSnbase/), quantreg for quantile regression, and finally doSNOW and foreach to manage parallel processing for batch analysis of MS data.

## Data

The data in this manuscript comes from a diverse set of sources, including Reaxys database query results, PubChem database query results, Mass Spec data from an Orbitrap Fusion Lumos Tribrid mass spectrometer in the Croninlab at the University of Glasgow, and Molecular Assembly Numbers generated using C++ code written by Stuart Marshall, and Leroy Cronin.

### Reaxys Data

We collected 25 Million molecules from Reaxys with a molecular weight between 0-1000 (inclusive). Of those 25 million molecules we computed MA indices for 2.5 million. We've stored all 25 million molecules in a *pickled* pandas dataframe saved as "Figure 2- MA in Chemical Space/all\_molecules.pickle". The molecules for which we have computed MA indices are saved also as a pickled dataframe "Figure 2- MA in Chemical Space/computed\_MA.pickle" (.pickle is serialized python data format, the package required to read this format comes standard on python 3 installations, more information here https://docs.python.org/3/library/pickle.html). In Figure 2 we've subsampled the data, that data is availible as a csv "Figure 2- MA in Chemical Space/ma\_mw\_subsampled\_by\_mw.csv"

Each row of the dataframes corresponds to a single molecule, and the columns are named 'substanceid', 'molecularweight', 'inchi', 'pa', 'molweight', 'formula'. The 'substanceid' is a unique identifier for our own use. 'molecularweight' is an exact molecular weight of the compound to 3 decimal places, while 'molweight' is an estimate of the mass to higher precision. The 'inchi' column contains the InChI string of the molecule which we use to encode the structural information of the molecule. The 'formula' column contains the Hill formula of the compound. The 'pa' column contains the MA of the molecule.

### Mass Spec data

The mass spec data collected for this project can be divided into two different types of data. Single Molecules (which we sometimes refer to Standard Curve molecules) and Environmental Samples (which we sometimes refer to as mixtures).

All data was collected using an Orbitrap Fusion Lumos Tribrid mass spectrometer produced by Thermo Fisher Scientific. The details of sample prep and analysis are contained the Supplemental Information which is availible with the manuscript. Given the number and complexity of the samples, including the .RAW Thermo files or even all of the converted .mzML files would be too cumbersome. Here we've included processed data, example .mzML files for a couple samples, and the code we've used to process the data.

The example mzML files are all in the "mzMLs" directory.

The processed data for all the single molecules and an example output are in "Figure 3 - Single Molecules"
